# Ranking sports science and medicine interventions impacting team performance: a protocol for a systematic review and meta-analysis of observational studies in elite football

**DOI:** 10.1136/bmjsem-2024-002196

**Published:** 2024-09-13

**Authors:** Tiago Fernandes, Vincenzo Rago, Marta Castañer, Oleguer Camerino

**Affiliations:** 1 National Institute of Physical Education of Catalonia, University of Lleida, Lleida, Spain; 2 Faculty of Health and Sports Sciences, Universidade Europeia, Lisbon, Portugal; 3 IRBLLEIDA (Lleida Institute for Biomedical Research), Lleida, Spain

**Keywords:** Research methods, Evidence synthesis, Multidisciplinary team, Team sports, Soccer

## Abstract

This study protocol describes a systematic method to identify, collect and rank sports science and medicine interventions most associated with optimising team performance in elite football in observational studies. While numerous interventions, such as conditioning or injury prevention programmes, protective equipment, training periodisation, tactical decision-making, supplements, medication and hydration administration, have been associated with football players and team performance enhancement, there is a need to prioritise them to save resources and increase the efficiency of applications. Nevertheless, previous literature has shown that systematic reviews in elite football often need more protocol registration and have limited procedures, synthesis and practical implications directly applicable to the field. Therefore, this protocol outlines a comprehensive process developed following the Cochrane Collaboration and Preferred Reporting Items for Systematic Reviews and Meta-Analyses statement comprising the following stages: (1) research question formulation and preliminary study, (2) eligibility criteria, (3) search strategy, (4) study selection, (5) data collection, (6) data assessment and (7) data synthesis and statistical analysis. It also presents a data quality standard process incorporating human and large language models reviewers and a detailed flow diagram for selecting suitable quantitative synthesis and ranking techniques. It includes meta-regression, pairwise, network, Bayesian or hierarchical meta-analysis options. The project associated and pre-registration of the protocol is available on the Open Science Framework (https://osf.io/tzcxq/).

**WHAT IS ALREADY KNOWN ON THIS TOPIC**

- Holistic and multidisciplinary approaches to sports science and medicine in football have been growing to study performance optimisation, creating the need to synthesise information for more effective practices.
- Peer review and registration of protocols enhance the quality, transparency and reproducibility of systematic reviews, addressing current issues in football-related systematic reviews, such as questionable methodologies and conclusions.

**WHAT THIS STUDY ADDS**

- This protocol establishes a comprehensive process for conducting systematic reviews and meta-analyses of sports science and medicine observational studies focused on team performance.
- It presents a standard process for ensuring high-quality data extraction, assessment and screening using independent blinded experts and large language models.
- A detailed decision flow chart is available to guide researchers in selecting appropriate quantitative syntheses, such as meta-regression, pairwise, network, Bayesian or hierarchical meta-analysis.

**HOW THIS STUDY MIGHT AFFECT RESEARCH, PRACTICE OR POLICY**

- This study protocol can significantly improve systematic reviews’ consistency, replicability and applicability in sports science and medicine.
- It promotes a multidisciplinary approach centred on a common objective, potentially encouraging more holistic and coordinated practices.
- Integrating advanced natural language processing and data analysis technologies establishes a precedent for reliable and quicker future systematic reviews and data sharing.

## Introduction

 Holistic and multidisciplinary approaches to sports science and medicine have demonstrated that injury prevention programmes,^1^ nutrition, medicine and hydration administration,^2^ psychological techniques,^3^ travel and equipment-related modalities,^4^ training periodisation^5 6^ and tactical decision-making^7^ can enhance athlete and team performance in football. In this regard, designing and implementing them is a critical responsibility for the multidisciplinary team, including coaches, medical specialists, nutritionists and psychologists, as it requires efficient communication, substantial resources and careful evidence-based information management to avoid inefficiencies and suboptimal practices.^8 9^ Therefore, there is a need for a systematic method to gather and prioritise that information, and a promising solution provided by research methodologies is the use of systematic review methods.^710^,^12^

Nevertheless, previous systematic reviews of observational studies in football have shown very low confidence ratings, often needing more protocol registration, neglecting to focus on team-level analyses and disregarding multidimensional relationships. In addition, most systematic reviews do not clearly define population, intervention or exposure, comparison, outcome and study design (PICOS), which could explain the absence of team performance meta-analyses or comprehensive syntheses directly applicable to coaching and team performance.^12^ Additionally, the absence of meta-analyses may be attributed to original observational studies showing several methodological discrepancies, a lack of replication and limited consistency.^7 12 13^

Although observational studies can reveal insights about the real world, they differ among themselves and from the standardisation found in experimental studies. The latter is impractical in football due to its complex and dynamic nature, whereas the former integrates these factors and attempts to understand them.^7 14^ Thus, it is crucial to standardise methods carefully, define exposures or interventions and identify confounders to ensure that the effects reflect the real-world unbiased.^13 14^

In this review, the term sports science and medicine interventions (or exposures) is delimited as team match interventions, referring to the deliberate strategies, programmes, prescriptions or actions implemented on teams or players by the disciplinary team according to Dijkstra *et al*,^8^ to influence or modify team match performance and, subsequently, their success or effectiveness.^8 14^

Nevertheless, choosing an approach to study success in football is not consensual, mainly due to the short-term, middle-term and long-term outcomes or the dynamic and complex process implicated.^15^ For instance, key performance indicators (KPIs) often describe successful performances but lack interpretability and utility.^13^ Although several studies have investigated the selection of KPIs to explain or predict mid-term or long-term goals or processes, few have examined whether or how changes in KPIs can contribute to those outcomes.^13^ For the latter type of inference, often seen in machine learning approaches, the reported information typically focuses on model accuracy rather than the directionality effects of the estimations. This lack of directionality and interpretation limits practitioners’ ability to use the findings effectively.^13 14^

The PICOS^11^ format research question of the systematic review is ‘Which interventions most impact team match performances and success in elite football according to observational studies?’. The following objectives were formulated: (1) to identify and collect interventions and confounding variables related to team match performance and success, (2) to synthesise and rank the retrieved interventions and (3) to perform a sensitivity analysis controlled by studies’ characteristics and confounders. Accordingly, the formal hypotheses are as follows^5^,^712 13 16 17^: (1) interventions related to strategy and tactics have the most significant impact on team match performances and success and (2) the effects of interventions on team match performances and success decrease when controlled for contextual variables.

## Methods

**Figure 1:**
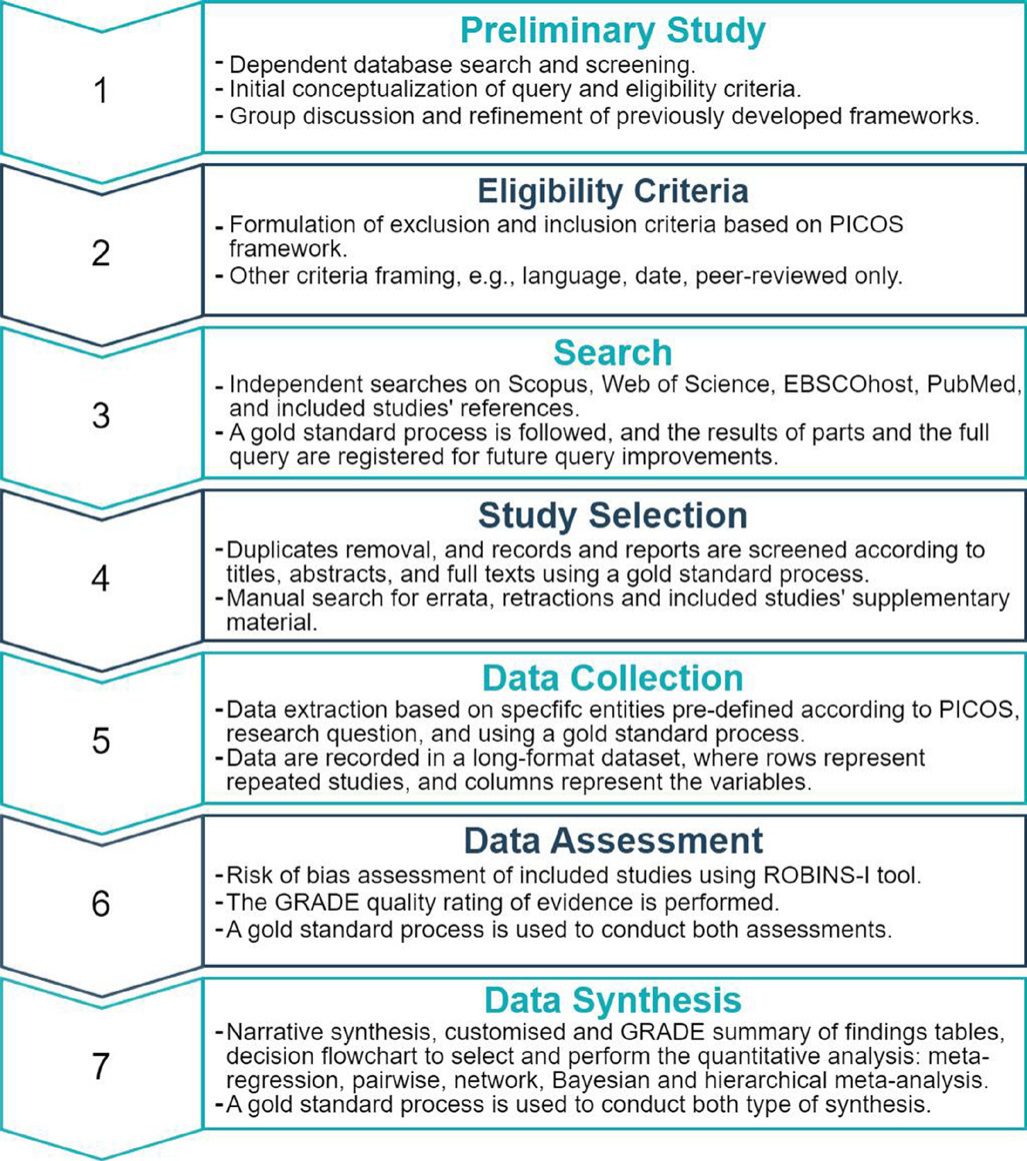
Comprehensive process of the systematic review. GRADE, Grading of Recommendations Assessment, Development and Evaluation. PICOS, Population, Intervention, Comparison, Outcome and Study Design. ROBINS-I, Risk Of Bias In Non-randomised Studies - of Interventions.

The systematic review is based on the Cochrane Collaboration framework,^11^ PRISMA 2020^18^, and the guidelines of various works specialised in systematic reviews and meta-analyses.^10^ Similar previous works are also considered.^7 12^ Simultaneously, the current protocol and systematic review are supported by the Automatic Research Synthesis (ARS) software through a semiautomatic approach (see generated protocol without human edition in online supplemental file 1. The authors double-check and edit the software output to meet the review requirements. In a cross-method, this protocol has the following stages: (1) research question formulation and preliminary study, (2) eligibility criteria, (3) search strategy, (4) study selection, (5) data collection, (6) data assessment and (7) data synthesis and statistical analysis (figure 1). The protocol is registered in the Open Science Framework (https://osf.io/tzcxq/), which the template to track tasks and enter the data from studies will be updated (online supplemental file 2), following the standard instructions provided to reviewers (online supplemental file 3). This protocol was also developed based on the Preferred Reporting Items for Systematic Review and Meta-Analysis Protocols (PRISMA-P)^19^ (see online supplemental file 4).

### Eligibility criteria

Inclusion and exclusion criteria were applied according to PICOS.^11^ They are described in the following subsections; the scale and its criteria are presented in online supplemental table 5.

#### Participants, population (P)

The participants include teams of elite adult male football players participating at a national team level or first-class division, corresponding to tiers 4 and 5 of the participant classification framework applied to team sports.^20^ The group’s age categorisation at the beginning of the season must be above 19 years.^21^ Teams with players under 19 but age categorisation above that age (eg, under-21) are included. Studies are excluded if their unit analysis is not team match observations, for example, penalties, the number of possessions or set pieces.

#### Interventions or exposures (I)

The interventions or exposures are related to team match performance (ie, match statistics) and success as dependent variables (ie, match outcomes, team ranking and the number of points). Only the interventions defined at the beginning of this review are included. They can be prematch, in-match and postmatch and from various domains, including but not limited to:

- Fitness or medical conditioning programmes, for example, FIFA 11+programme.^1^
- Nutritional, medication and hydration administration, for example, supplements and medication.^2^
- Psychological techniques: team communication and cohesion.^3^
- Strategical and tactical decisions, for example, game styles and patterns.^7^
- Travel and equipment-related interventions, for example, protective equipment.^4^
- Training periodisation, for example, weekly and competitive load.^5 6^

#### Comparisons (C)

The comparators will be optional but include the interventions’ groups, allowing for direct comparison of their impact on team performance and success between different or within the same domain.

#### Outcomes (O)

The primary outcomes are the following according to the following premises: the competition is determined by a three-point rule, in which the winning team consists of the team with the greater number of goals and points, there is uncertainty in short outcomes or match-level, and medium-term outcomes or the season’s results, and football is a zero-sum game.^15 17^ Based on these premises, the following outcomes were included: goals scored, goals conceded, goals difference, the number of winnings, the number of losses, the difference between winning and losses, categorical and dichotomous match outcomes, team final ranking, number of points and difference of number of points. In addition, the secondary outcomes will be related to team match performance, including but not limited to the physical, for example, accelerations and total distance^5^; technical, for example, dribbles, shots on goal^16^; tactical, for example, transition-state attack/defence, defence subphase^7^; and medical, for example, injury incidence.^22^

#### Study designs (S)

The systematic review includes case–control, cross-sectional and longitudinal or cohort observational studies.

#### Others (date, language, source and statistical reporting)

Articles in any language or date are considered. However, non-English papers will be translated using machine translation. Only studies published in peer-reviewed journals with quantitative statistical models or machine learning techniques will be included.

### Information sources

The information sources for the systematic review are the following electronic databases: (1) EBSCOhost (MLA International Bibliography with Full Text, Library, Information Science & Technology Abstracts, CINAHL Plus, GreenFILE, Teacher Reference Center, eBook Collection and Collection (EBSCOhost), MathSciNet via EBSCOhost, MLA Directory of Periodicals, PSICODOC, eBook Open Access), 2) Web of Science (Web of Science Core Collection, Current Contents Connect, Derwent Innovations Index, Grants Index, KCI-Korean Journal Database, MEDLINE, ProQuest, SciELO), (3) Scopus and (4) PubMed. The electronic databases will be accessed first by their Application Programming Interface integrated into ARS and manually. Additionally, reference lists of included studies and previous systematic reviews with similar topics will be considered. A search will be done with the same area terms (table 1) except for study design, which will be replaced by (“systematic review” OR “meta-analysis” OR “synthesis”). Only the systematic review of male football will be selected.

**Table 1:** Search terms used in the review by area and using the Boolean method

Area	Fields	Terms
Population	Title	(soccer OR football) AND (elite* OR professional* OR association) NOT “Australian Rules Football” NOT “Australian Football League” NOT “American Football” NOT “National Football League” NOT “Gaelic Football” NOT rugby NOT basketball NOT handball NOT volleyball NOT indoor NOT former NOT retired* NOT referee* NOT amateur* NOT academ* NOT youth NOT junior* NOT young* NOT colleg* NOT adolescent* NOT universit* NOT under-1? NOT female* NOT wom?n) AND
Intervention/comparison	Title, abstract and keyword, or topic	(intervention* OR decision* OR instruction* OR formation* OR strateg* OR substitut* OR program* OR change* OR constraint* OR method* OR practice* OR training OR coach* OR adjust* OR condition* OR protocol* OR load* OR warm-up* OR exercise* OR position* OR prevention* OR preparation* OR context* OR situation* OR half* OR halves OR match* OR game*) AND
Outcome	Title, abstract and keyword, or topic	(outcome* OR winn* OR win OR won OR lose OR loss OR losing OR victor* OR odds OR expect* OR probabili* OR result* OR success OR discriminat* OR score* OR action* OR metric* OR indicator* OR statistic* OR factor* OR rank* OR stand* OR goal* OR points OR performance* OR effect*) AND
Study design	Title, abstract and keyword, or topic	(“notational analysis” OR “performance analysis” OR “match analysis” OR “game analysis” OR observation* OR cross-sectional OR Cohort OR case-control OR longitudinal* OR analytics OR “machine learning” OR predict* OR classif*) NOT review NOT “meta-analysis” NOT synthesis NOT experimental

Note. Double quotation denotes searching for the term exactly as in parentheses. ‘?’” marks multiple characters that generate different words and require at least four characters before; * indicates that the term can have different endings.

* Indicates that the term can have different endings.

### Data quality

**Figure 2:**
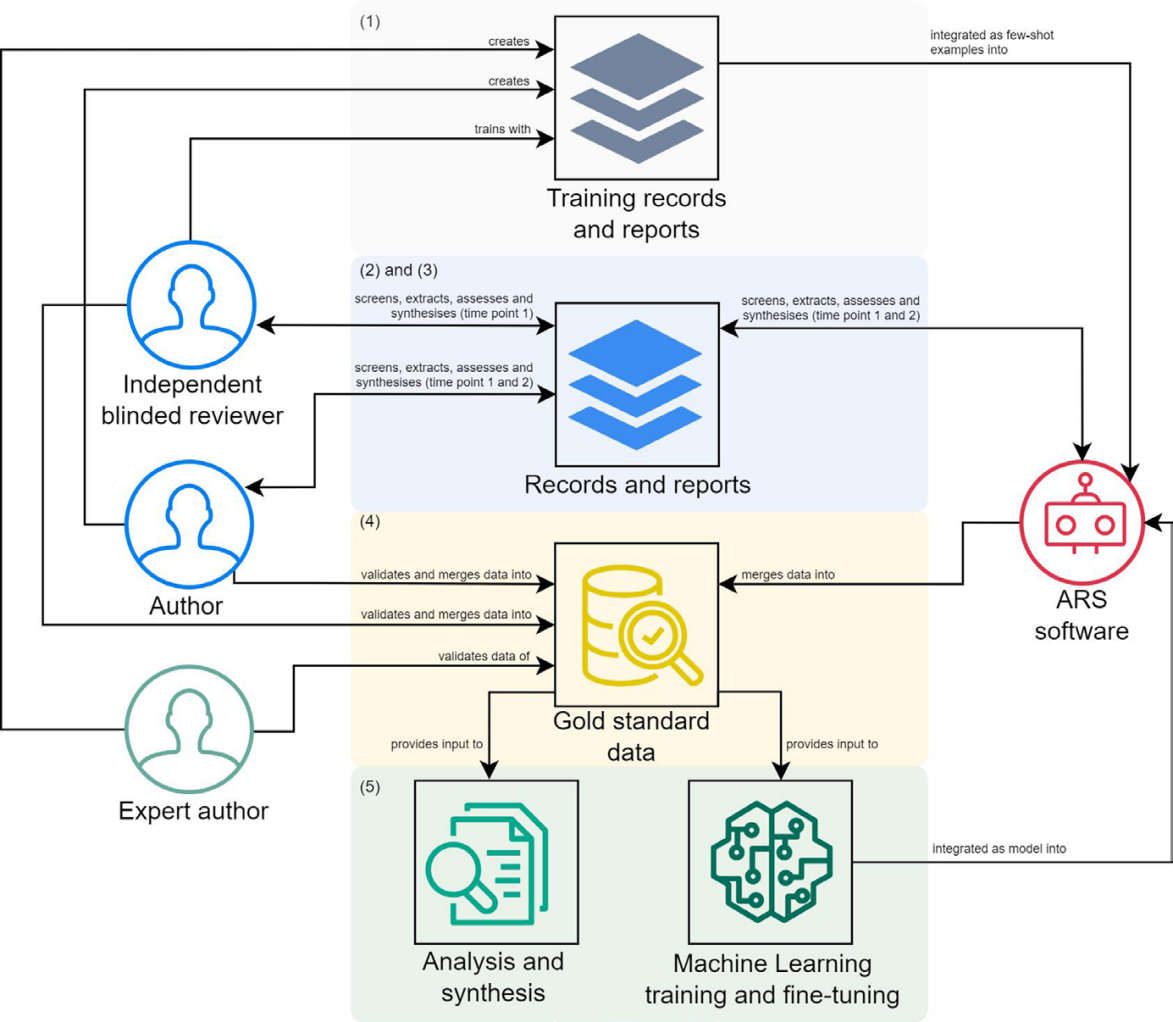
Data quality gold-standard process. ARS, Automatic Research Synthesis. Numbers (in parentheses) represent the stages of the process.

There is a standard process that the review will follow for data quality, more specifically in search, screening, data collection and assessment. This process, identified throughout the review as a gold standard, resembles human feedback and active learning approaches^23^ and consists of the following steps (figure 2):

1. The two-step training process of Acosta *et al*^24^ is adapted for each task. Online meetings are conducted for documentation and instruction familiarisation for the first step. Subsequently, three records or reports, depending on the task, related to the topic that possibly can be on the sample with different categorisations (low, intermediate and high level) and previously rated and completed by the first and second reviewers are provided to the independent expert (IE) and ARS (ie, few-shots learning). Since there is only one rater, all three were given instead of randomly selecting one, as the original authors suggested. After completion, the first and second authors provide feedback on the tasks completed by the IE for the three records or reports.
2. A first reviewer author, ARS and one IE with a PhD and peer-reviewed publications on research synthesis methods and sports, preferably specialised in football, will independently screen or collect or assess all the records and reports. The IE will be blind to the review’s research questions, hypotheses and the original studies’ authors and journals; the latter applies only when the journal is not an entity for extraction. The first reviewer author will contact the authors of primary studies to provide the full text for the non-accessible reports. A 1-week notice will be granted, followed by a reminder. The record is excluded if the authors fail to answer within 2 weeks from the first contact or provide the report.
3. Then, 2 weeks after the first screening and selection of studies, the first review author will screen, collect or assess the studies again to assess intrareliability. If intrareliability or inter-reliability is assessed as poor, slight or fair agreement^25^ the task will be repeated until at least moderate agreement is reached.
4. Afterwards, a gold standard will be created from the combined results to provide the final results list, with the second reviewer author assisting as an expert in any disagreements or uncertainties.
5. Finally, the gold standard is used for analysis and synthesis, with the potential for application in machine learning and fine-tuning, which can be implemented in ARS software but is not limited to it.

### Search strategy

The query builder was formulated based on a dependent preliminary search and screening performed by TF and OC (see online supplemental file 6). From the preliminary study, we found the studies known beforehand and concluded that hyponyms were preferred to avoid redundancy. The final query incorporates a Boolean conjunction method of four disjunction groups (table 1), and the final queries customised for each database are presented in online supplemental table 7.

Our search approach will follow the gold-standard process described in this paper. The search strategy follows a three-step approach:

1. The author TF, the IE and ARS will perform a search independently in the information sources without filters.
2. The author TF, the IE and ARS will independently perform a citation search in the included studies and relevant systematic reviews.
3. The retrieved records will be aggregated in a unique Excel file.

### Study records

#### Data management

EndNote software V.21 and Microsoft Excel (2406) will manage references for manual searching on the information sources’ websites.

#### Selection process

A three-step approach based on the PRISMA statement 2020^18^ will be followed for the screening and selection of studies:

1. Duplicates will be removed using ARS and then double-checked manually. A non-duplicated articles list with an exclusive number identification, title, authors, journal and DOI will be provided for the next stage.
2. Reports will be retrieved by EndNote V.21 and ARS software using the University of Lleida private virtual network. The author TF will constitute the first reviewer, and OC, VR or MC will be the second reviewer of the gold standard process.
3. Where applicable, a manual search will be conducted for errata, retractions and included studies' supplementary files.

#### Data collection process

For the data collection process, the author TF will constitute the first reviewer and OC, VR or MC the second reviewer of the gold-standard process to extract data from the included studies according to online supplemental file 2 and online supplemental table 8), respectively. The former is an Excel spreadsheet with precoded answers; the latter is a table with data descriptions to be extracted. Reports will only be included if information is essential for eligibility criteria.

### Data items

The data form was built using suggestions from literature and previous work.^7 10 12^ Briefly incorporates the following domains and fields, being susceptible to additions according to data collection:

- Metadata: study unique identification, year, journal.
- Population: country, competition, year of competition, number of teams’ match observations included (n) and excluded (n), the reason for exclusion, the number of teams (n), the number of players (n), the number of players’ observations (n).
- Interventions: theme, term, definition, type (1=sports science; 2=sports medicine; 3=other), method (1=conditioning and training programmes; 2=nutritional and hydration strategies; 3=psychological techniques; 4=strategical and tactical decisions; 5=training periodisation; 6=injury related; 7=equipment related; 8=other), frequency, duration, intensity (1=low intensity; 2=moderate intensity; 3=vigorous intensity; 4=high intensity; 5=submaximal intensity; 6=maximal intensity), match halves (1=pre-first half; 2=in-first half; 3=pre-second half, 4=in-second half), match period (1=prematch; 2=in-match; 3=postmatch), phase of the game (1=defensive; 2=offensive; 3=transition attack defence; 4=transition defence attack; 5=set pieces; 6=globally).
- Outcome: term, definition, unit (1=count; 2=metres; 3=m/s; 4=m/s^2^; 5=ratio; 6=percentages; 7=difference; 8=arbitrary unit; 9=probabilities; 10=other), type (1=team success; 2=team match performance; 3= team match effects), phase of the game (same categories as the one in interventions).
- Comparisons: intervention 1, intervention 2, outcome, subgroup, control variable, covariates, mean, SD, sample size, proportions and total in proportions for intervention 1 and 2; p value, effect size (ES) value, ES 95% CI, ES SE, ES reported (1=OR; 2=log odds ratio; 3=Cohen’s d; 4=Hedges’ g; 5=standardised coefficients; 6=other; 7=none) and ES type (1=crude; 2=adjusted).
- Study design: The type of study (1=case–control; 2=cross-sectional; 3=cohort, 4=longitudinal; 5=other), instrument name, instrument type (1=semiautomatic or full-automatic tracking systems; 2=global positions systems; 3=databases/websites; 4=notational or observational instruments; 5=survey; 6=other), instrument validity, instrument inter-reliability, instrument intrareliability (1=not stated; 2=unclear; 3=stated; these codes are applicable for the past two entities), data analysis approach (1=statistical modelling; 2=machine learning modelling), inferential paradigm (1=frequentist; 2=Bayesian), statistical tests/machine learning techniques’ name, preprocessing/data cleaning description, model evaluation description, machine learning problem type (1=prediction; 2=classification; 3=clustering; 4=other), statistical/machine learning analysis type (1=univariate; 2=bivariate; 3=multivariate), statistical tests/machine learning techniques methods (1=differences; 2=associations and correlations; 3=regression).

### Risk of bias in individual studies

The risk of bias in studies is assessed using ROBINS-I.^26^ The author TF will constitute the first reviewer, and OC, VR or MC will be the second reviewer of the gold-standard process to assess the risk of bias in studies using ROBINS-I. ARS will create a table and stacked horizontal graph of the assessments with the gold standard answers.

#### Confounding variables

The confounding variables include team events on matches, which correspond to the secondary outcome, teams’ characteristics and contextual variables. It will consist of participant-level characteristics, including but not limited to temperature, relative humidity, web-bulb globe temperature, altitude, type of competition, match status, crowd, game time, stadium size match location.^5 7^

### Data synthesis

#### Qualitative synthesis

A narrative synthesis will be performed, and results will be displayed on a summary of findings tables with sample characteristics, measurement instruments, predictors, contextual variables and critical results headers (online supplemental table S9). In addition, a table with variables collected and showing relationships to the outcome will be constructed to intuitively evidence the variables most studied and the impact on the outcome (see a template in online supplemental table S10).

#### Quantitative synthesis

**Figure 3:**
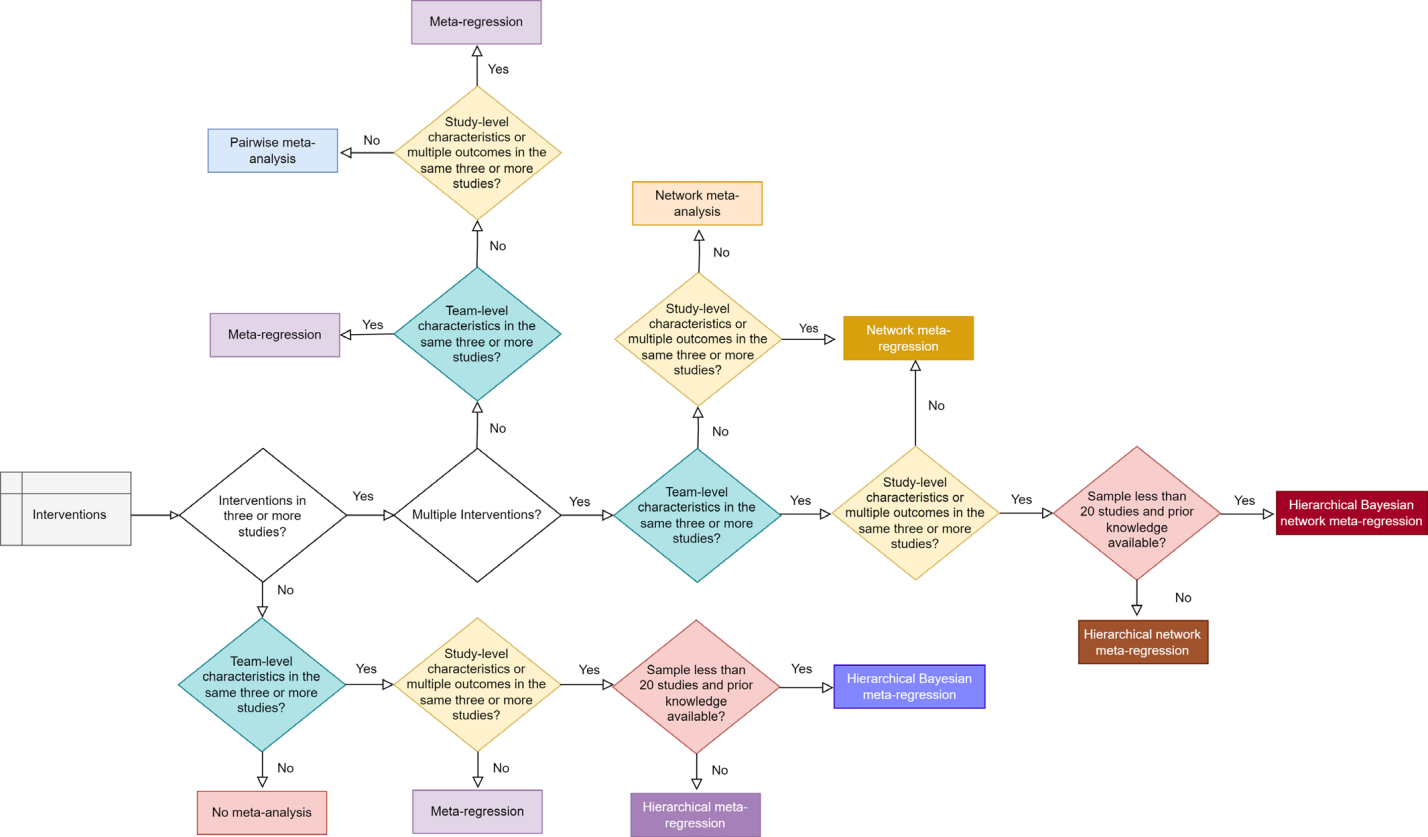
Decision flow chart of the quantitative synthesis.

Data summaries using means, SD, absolute and relative frequencies and percentages will be used to report samples and instrument content. The strategy of figure 3 will be followed to do a meta-analysis. All meta-analysis statistics will be performed using R software V.4.4.1 with meta, metafor, netmeta, R2OpenBUGS packages and OpenBUGS V.3.2.3 for Bayesian. The scripts with base code and models are available in online supplemental file 11.

The Grading of Recommendations Assessment, Development and Evaluation scheme will summarise findings and assess evidence quality in quantity synthesis accordingly. The very low, low, moderate and high-quality rates will be used to assess study limitations, indirectness, inconsistency, imprecision and publication bias.^11 18 19^

#### Types of data and calculations

ORs, 95% CIs and SEs will be extracted for binary outcomes. If missing data, the OR will be calculated using the number of events and total events per group. As for continuous outcomes, standardised beta coefficients will be extracted and transformed to Hedges’ g. Alternatively, means, SD and sample numbers will be used to calculate Hedges’ g. Finally, other formulas to convert other ES (eg, Cohen’s d) to Hedges’ g and OR will be considered.^11^ All the data will be extracted and organised in a proper sheet of online supplemental file 2.

#### Pairwise meta-analysis

A pairwise meta-analysis will be performed for at least three studies for the same intervention related to the same outcome. The summary of effects measure will be OR and Hedges’ g for dichotomous and continuous scale, respectively. Missing data will be assumed to be missed randomly, and sample stratification exposed to the same variable will be combined, where applicable. When it is not, it will be inserted, and the influence of that stratification, for example, match location, will be analysed by sensitivity analysis. Longitudinal studies’ single time points will be considered as different studies, and the OR of multinominal models will be considered if the reference consists of an intervention included in the review.

A fixed and random effects model, DerSimonian and Laird, will be conducted, and heterogeneity will be calculated through inconsistency (I²) statistics, in which a 0.10 level of significance is considered to test the null hypothesis that all studies present the exact effect estimates. The heterogeneity percentage of the variability in effect is interpreted as might be important (0%–40%) or may have moderate (30%–60%), substantial (50%–90%) or considerable (75%–100%) heterogeneity.^11^ If heterogeneity is present, a random effects model will be chosen, and subgroup analysis and meta-regression using study characteristics and risk of bias assessment will be conducted.

#### Network meta-analysis

Network meta-analysis (NMA) will follow the assumptions and interpretations of the pairwise meta-analysis regarding the number of studies, summary measure of effect, significance level and homogeneity, where applicable. The CINeMA approach will be followed to assess within-study and across-study bias, indirectness, imprecision, heterogeneity and incoherence.^27^

Some assumptions are immediately violated in observational studies due to their nature, for example, joint randomisability. Therefore, meta-regression is proposed as a sensitivity analysis to address bias (eg, ROBINS-I), analyse consistency and compare results. Despite non-randomisation, interventions will only be compared if they are similar and mutually exclusive (eg, tactical formation). This means that if one intervention exists in a group, it cannot have another. Inconsistency assessment will be done through node-splitting analysis. If any inconsistency is identified, we will explore evidence of source agreement, evaluate model fit using goodness-of-fit and diagnostics statistics (eg, Akaike Information Criterion (AIC), Bayesian Information Criterion (BIC), Deviance Information Criteria (DIC)) and compare models and visual inspection (eg, forest and network plots).

We will consider the number of studies to draw the edges for the geometry of networks, and networks for subgroups will be equated (eg, by players’ position). Potential bias of study design (ie, case, cross-sectional and cohort studies) and other types will be considered. Studies separated from the network map (ie, those that did not use similar variables) will be excluded. Treatment rankings will be estimated using P-scores.^28^

#### Meta-regression

A multiple weighted least squares regression with covariates (eg, match-statistics, study-level characteristics and subgroups) will be performed to assess the robustness of summary effects of pairwise and NMA if data are available. The covariates were listed in the outcome and confounding sections. Multilevel studies with teams, subgroups, and studies, or team outcomes and studies at levels 1, 2 and 3, respectively, will also be conducted, where applicable. A multimodel inference approach will be applied to select the predictors to include in the model. R^2^ and τ^2^ will estimate the percentage of variance of the model and between-study heterogeneity, respectively. A 95% CI, p-levels (α=0.05) and t-statistics will be reported. Bubble plots will also be plotted for visualisation.^29^

#### Bayesian models

The choice of Bayesian models will depend on the characteristics and quantity of data collected from studies. A Bayesian approach will be preferable to a frequentist approach for smaller sample sizes, potential hierarchical structures, and the strength and availability of prior information. The hierarchical Bayesian model will follow the same structure as referred to in the meta-regression sections. For the selection of prior distributions in Bayesian analyses, we anticipate a binomial likelihood and Gaussian likelihood for binary and continuous outcomes, respectively, weakly informative or uninformative prior distributions for the predictors’ effects (µ), and a Half-Cauchy prior distribution for the common heterogeneity variance (τ²).

Reporting will include 95% credible intervals and predictive intervals. Gibbs sampling Markov Chain Monte Carlo will be conducted to estimate pooled measures with 4 chains, 100 000 iterations, a 10-thinning factor and 5000 for burn-in. Convergence will be inspected visually and confirmed with the Brooks-Gelman-Rubin test. Model comparison will be done using DIC and the heterogeneity variance (τ²). The surface under the cumulative ranking score and probabilities will be calculated to rank predictors.^30^

#### Meta-biases, sensibility analysis and robustness

In addition to meta-regression, transversal to all types of meta-analysis, the three following analyses for robustness will be considered^11^:

- Egger’s test and funnel plots to assess publication bias (eg, reporting bias).
- Subgroup analysis to test heterogeneity across subgroups (eg, match location, opposition quality, players’ positions or roles).
- A sensitivity analysis will compare the primary analysis with fixed-effects models and remove small studies.

### Statistical analysis

The consistency of screening, data extraction and assessment will be assessed using stability and objectivity (ie, intracoder and intercoder reliability, respectively) using coefficient Kappa interpreted as follows^25^: poor (<0.00), slight (0.00–20), fair (0.21–0.40), moderate (0.41–0.60), substantial (0.61–0.80) and almost perfect (0.81–1.00). Textual answers will be assessed using a similarity score between texts. Intraclass correlation coefficient and 95% CI will be calculated using one-way random effects and will be interpreted as poor (<0.40), fair (0.40–0.59), good (0.60–0.74) and excellent (0.75–1.00). The ARS and R software (V.4.4.1) will be used for plotting descriptive and inferential statistics.

## supplementary material

10.1136/bmjsem-2024-002196 online supplemental file 1

10.1136/bmjsem-2024-002196 online supplemental file 2

10.1136/bmjsem-2024-002196 online supplemental file 3

10.1136/bmjsem-2024-002196 online supplemental file 4

10.1136/bmjsem-2024-002196 online supplemental file 5

10.1136/bmjsem-2024-002196 online supplemental file 6

10.1136/bmjsem-2024-002196 online supplemental file 7

10.1136/bmjsem-2024-002196 online supplemental file 8

10.1136/bmjsem-2024-002196 online supplemental file 9

10.1136/bmjsem-2024-002196 online supplemental file 10

10.1136/bmjsem-2024-002196 online supplemental file 11

## Data Availability

All data relevant to the study are included in the article or uploaded as online supplemental information.

## References

[1] Støvland VR, Amundsen R, Paulsen G (2023). Prepare to fail or failing to prepare? Acute performance after the 11+ with and without strength exercises. BMJ Open Sport Exerc Med.

[2] Oester C, Weber A, Vaso M (2019). Retrospective study of the use of medication and supplements during the 2018 FIFA World Cup Russia. BMJ Open Sport Exerc Med.

[3] Ekstrand J, Lundqvist D, Davison M (2019). Communication quality between the medical team and the head coach/manager is associated with injury burden and player availability in elite football clubs. Br J Sports Med.

[4] Azad AM, Al Juma S, Bhatti JA (2016). Modified Balance Error Scoring System (M-BESS) test scores in athletes wearing protective equipment and cleats. BMJ Open Sport Exerc Med.

[5] Rago V, Rebelo A, Krustrup P (2021). Contextual Variables and Training Load Throughout a Competitive Period in a Top-Level Male Soccer Team. J Strength Cond Res.

[6] Hernandez D, Sanchez M, Martin V (2021). Contextual variables and weekly external load in a semi-professional football team. Apunts Educ Fis Deporte.

[7] Fernandes TMG (2017). Tactical-technical performance analysis in high-performance soccer: sequential analysis study of defensive phase and transition attack-defense of FIFA World Cup 2014 semifinalists teams.

[8] Dijkstra HP, Pollock N, Chakraverty R (2014). Managing the health of the elite athlete: a new integrated performance health management and coaching model. Br J Sports Med.

[9] Abreu R, Figueiredo P, Beckert P (2021). Portuguese Football Federation consensus statement 2020: nutrition and performance in football. BMJ Open Sport Exerc Med.

[10] Cooper H (2010). Research synthesis and meta-analysis: a step-by-step approach.

[11] Higgins JPT, Green S (2008). Cochrane handbook for systematic reviews of interventions.

[12] Sarmento H, Clemente FM, Afonso J (2022). Match Analysis in Team Ball Sports: An Umbrella Review of Systematic Reviews and Meta-Analyses. Sports Med Open.

[13] Morgulev E, Lebed F (2024). Beyond key performance indicators. Ger J Exerc Sport Res.

[14] Rosenbaum PR (2002). Observational studies.

[15] van der Burg T (2023). Competitive balance and demand for European men’s football: a review of the literature. Manag Sport Leisure.

[16] Castañer M, Barreira D, Camerino O (2017). Mastery in Goal Scoring, T-Pattern Detection, and Polar Coordinate Analysis of Motor Skills Used by Lionel Messi and Cristiano Ronaldo. Front Psychol.

[17] Villa G, Lozano S (2016). Assessing the scoring efficiency of a football match. Eur J Oper Res.

[18] Page MJ, Moher D, Bossuyt PM (2021). PRISMA 2020 explanation and elaboration: updated guidance and exemplars for reporting systematic reviews. BMJ.

[19] Shamseer L, Moher D, Clarke M (2015). Preferred reporting items for systematic review and meta-analysis protocols (PRISMA-P) 2015: elaboration and explanation. BMJ.

[20] McKay AKA, Stellingwerff T, Smith ES (2022). Defining Training and Performance Caliber: A Participant Classification Framework. Int J Sports Physiol Perform.

[21] Mills A, Butt J, Maynard I (2014). Toward an understanding of optimal development environments within Elite English Soccer Academies. Sport Psychol.

[22] Jones S, Almousa S, Gibb A (2019). Injury Incidence, Prevalence and Severity in High-Level Male Youth Football: A Systematic Review. Sports Med.

[23] Tan Z, Beigi A, Wang S (2024). Large language models for data annotation: a survey. arXiv.

[24] Acosta S, Garza T, Hsu H-Y (2020). Assessing quality in systematic literature reviews: a study of novice rater training. Sage Open.

[25] Landis JR, Koch GG (1977). The measurement of observer agreement for categorical data. Biometrics.

[26] Sterne JA, Hernán MA, Reeves BC (2016). ROBINS-I: a tool for assessing risk of bias in non-randomised studies of interventions. BMJ.

[27] Cameron C, Fireman B, Hutton B (2015). Network meta-analysis incorporating randomized controlled trials and non-randomized comparative cohort studies for assessing the safety and effectiveness of medical treatments: challenges and opportunities. Syst Rev.

[28] Seide SE, Jensen K, Kieser M (2020). A comparison of Bayesian and frequentist methods in random-effects network meta-analysis of binary data. Res Synth Methods.

[29] Harrer M, Cuijpers P, Furukawa TA (2021). Doing meta-analysis with R: a hands-on guide.

[30] Hutton B, Salanti G, Caldwell DM (2015). The PRISMA extension statement for reporting of systematic reviews incorporating network meta-analyses of health care interventions: checklist and explanations. Ann Intern Med.

