# Supplementary material for: Ranking sports science and medicine interventions impacting team performance: a protocol for a systematic review and meta-analysis of observational studies in elite football
Source: BMJ Open Sport Exerc Med. 2024 Sep 13;10(3):e002196. doi: 10.1136/bmjsem-2024-002196 (PMC11404162; doi:10.1136/bmjsem-2024-002196)
Supplement: online supplemental file 1 [file bmjsem-10-3-s001.pdf]

Supplementary File S1: Protocol generated by ARS software

Which interventions most impact team match performances and success in elite football according to observational studies?

Format:

PICOS

Decomposition of research question

P (Population): elite football players
I (Intervention): various interventions
C (Comparison): no intervention or other interventions
O (Outcome): team match performances and success
S (Study Design): observational studies

Scopus Queries:

(TITLE-ABS-KEY("Association football" OR soccer) AND TITLE-ABS-KEY("elite" OR "professional") AND TITLE-ABS-KEY(team* performance OR team* success))

Pubmed Queries:

("soccer"[Title/Abstract] OR "Association football"[Title/Abstract]) AND ("elite"[Title/Abstract] OR "professional"[Title/Abstract]) AND (team performance[Title/Abstract] OR team success[Title/Abstract])

Web of Science Queries:

TS=("Association football" OR soccer) AND (TITLE-ABS-KEY("elite") OR TITLE-ABS-KEY("professional")) AND TS=(team* performance OR team* success)

Inclusion Criteria:

1. Population: elite football teams
2. Intervention: interventions affecting team match performance and success, including but not limited to coaching, training regimes, diet, sports psychology, and equipment
3. Comparison: no intervention or other interventions for comparison
4. Outcome: team match performances and success, e.g., win-loss record, goals scored, and league position
5. Study design: observational studies such as cohort, case-control, or cross-sectional studies published in peer-reviewed journals

Exclusion Criteria:

1. Studies involving non-elite football teams;
2. Interventions that do not affect team performance or success, or involve a combination of interventions;
3. Randomized controlled trials and experimental studies;
4. Outcomes other than team match performances and success;
5. Study designs other than observational studies (e.g., randomized controlled trials, cohort studies, case-control studies, cross-sectional studies).

Data Form:

Name: Generated tiagomgfernandes 1

Description:

Authors:
Year of publication:
Setting:
Methods:
total sample size:
Sample characteristics:
  P: elite football players
intervention description:
  I: various interventions
comparator:
  C: no intervention or other interventions
intervention period:
follow-up period:
theoretical basis:
measured outcomes:
  O: team match performances and success
measurement instruments and methods:
summary of findings:

Synthesis Form:

Name: Generated tiagomgfernandes 1

Description:

Summary of findings:
  Authors:
  Year:
  Objective:
  Design:
  Sample:
  Intervention:
  Comparison:
  Outcome:
    Team match performances and success:
  Results/Conclusions:

Data Assessment:

Name: Generated tiagomgfernandes 1

Description:

tool:
  Questions 1 - Bias due to confounding:
    1.1: [Is there potential for confounding of the effect of training interventions in sports studies?, Y / PY / PN / N, PN / N]
    1.2: [Was the analysis based on athletes' performance over time according to training received?, NA / Y / PY / PN / N / NI,]
    1.3: [Were training discontinuations likely related to athlete's physical condition?, NA / Y / PY / PN / N / NI,]
    1.4: [Did the authors use appropriate methods to control for fitness level and other confounding factors?, NA / Y / PY / PN / N / NI, Y / PY]
  Questions 2 - Bias in selection of participants into the study:
    2.1: [Was selection of athletes based on performance observed after the start of training?, Y / PY / PN / N / NI, PN / N]
  Questions 3 - Bias in classification of interventions:
    3.1: [Were training groups clearly defined?, Y / PY / PN / N / NI, Y / PY]
  Questions 4 - Bias due to deviations from intended interventions:
    4.1: [Were there deviations from the intended training beyond what would be expected in usual practice?, Y / PY / PN / N / NI, PN / N]
  Questions 5 - Bias due to missing data:
    5.1: [Were performance data available for all, or nearly all, athletes?, Y / PY / PN / N / NI, Y / PY]
  Questions 6 - Bias in measurement of outcomes:
    6.1: [Could the performance measure have been influenced by knowledge of the training received?, Y / PY / PN / N / NI, PN / N]
  Questions 7 - Bias in selection of reported result:
    7.1: [Is the reported effect estimate likely to be selected based on results from multiple time points?, Y / PY / PN / N / NI, PN / N]
  Overall:
    Critical: [at least one critical]
    High/Serious: [at least one high/serious and no critical]
    Moderate: [low or moderate]
    Low: [all low]
    No information: [No information is reported about selection of athletes or whether start of follow up and start of training coincide.]
  labels:
    0: No information
    1: Low
    2: Moderate
    3: High/Serious
    4: Critical
    -1: No reported
  judgement:
    Critical: 3
    High/Serious: 2
    Moderate: 1
    Low: 0
    No information: -1
    No reported: -2

Journal Template Form:

Name: Generated tiagomgfernandes 1

Description:

Submission Guidelines:

- Article Type: Systematic Review on Sports Sciences
- Word Count Limit: 8000
- Abstract Length:
  - Standard: 200
  - Extended: 350
- Tables and Figures: Up to 10 tables and 15 figures
- References: Up to 150
- Text Formatting:
  - Font: Normal, plain (e.g., 12-point Arial)
  - Page Numbering: Automatic
  - File Format: [Word, LaTeX]
- Supplementary Information: Accepted in formats including training datasets and competition videos
- Reporting Guidelines: Follow PRISMA adapted for sports research
- Digital Features: [Animated abstracts showing sports techniques, Video abstracts of sports performance analysis, Slide decks on sports studies, Audio slides on athlete interviews, Instructional videos on training methods, Infographics on sports statistics, Podcasts on sports science, Animations of gameplay strategies]
- Peer Review: External and confidential, with sports experts
- Open Access Options: Available with specific sports science journals

Checklist Form:

Name: Generated tiagomgfernandes 1

Description:

- Title:
  - Essential Elements: [Identify the report as a systematic review on sports performance enhancement, Report an informative title with key information about the main objective or question related to sports sciences]
- Methods:
  - Search Strategy:
    - Essential Elements: [Provide the full search strategy as run in each sports database, Describe any methods used to synthesize data collected from sports studies]
  - Information Sources:
    - Essential Elements: [Specify the date when each sports database was last searched, Describe any limits applied to the search strategy in sports contexts]
  - Eligibility Criteria:
    - Essential Elements: [Specify all sports disciplines characteristics for inclusion, Specify eligibility criteria with regard to athlete performance metrics]
- Results:
  - Study Selection:
    - Essential Elements: [Report the number of records identified, excluded, screened, and included in sports performance research]
  - Study Characteristics:
    - Essential Elements: [Cite each included study on sports, Present key characteristics of each sports study]
- Abstract:
  - Essential Elements: [Report an abstract addressing each item in the PRISMA 2020 for Abstracts checklist focusing on sports analytics]
- Discussion:
  - Limitations:
    - Essential Elements: [Discuss any limitations of the evidence included in sports science, Discuss implications of the results for sports practice and policy]
- Introduction:

Rationale:

Essential Elements: [Describe the current state of knowledge in sports training and its uncertainties, Explain the importance of the review in identifying effective training methodologies]

Objectives:

Essential Elements: [Provide an explicit statement of all objectives or questions related to sports performance]

Other Information:

Competing Interests:

Essential Elements: [Disclose any of the authors' relationships or activities in the sports industry]

Acknowledgements:

The automatic research synthesis (ARS) software was used to generate this protocol.
