# Supplementary material for: Ranking sports science and medicine interventions impacting team performance: a protocol for a systematic review and meta-analysis of observational studies in elite football
Source: BMJ Open Sport Exerc Med. 2024 Sep 13;10(3):e002196. doi: 10.1136/bmjsem-2024-002196 (PMC11404162; doi:10.1136/bmjsem-2024-002196)
Supplement: online supplemental file 2 [file bmjsem-10-3-s002.pdf]

Supplementary File S2
Template for task tracking, searches, screening, data collection and assessment forms

Instructions

- Please substitute '?' for the entry data on each sheet.
- For more detailed instructions please consult the Supplementary File S3 at repository

Reviewer identification

**ReviewerID**	?
**Time point**	?

Task tracking for the Systematic Review

Sheet number	Task	Track
1	Searches history	To Do
2	Searches results	To Do
3	Duplicates	To Do
4	Screening titles	To Do
5	Screening abstracts	To Do
6	Screening full-texts	To Do
2,3,4,5,6	Citation search and screening of included studies	To Do
2,3,4,5,6	Citation search and screening of systematic reviews	To Do
7	Data collection: metadata	To Do
8	Data collection: population	To Do
9	Data collection: interventions	To Do
10	Data collection: outcomes	To Do
11	Data collection: comparisons	To Do
12	Data collection: study design	To Do
13	Data assessment	To Do

Configuration

Sheet name	Description
**Legend**	It contains abbreviations' full forms
**Config**	It includes the drop-down menu items of categorical variables

Help and documentation

Database	Search Links	Help links
**Scopus**	https://www.scopus.com/search/form.uri?display=advanced	https://schema.elsevier.com/dtds/document/bkapi/search/SCOPUSSearchTips.htm
**Pubmed**	https://pubmed.ncbi.nlm.nih.gov/advanced/	https://pubmed.ncbi.nlm.nih.gov/help/#truncating-search-terms

**Web of Science**	https://www.webofscience.com/wos/woscc/advanced-search	https://images.webofknowledge.com/images/help/WOS/hs_wildcards.html
**Ebscohost**	https://search.ebscohost.com/	https://connect.ebsco.com/s/article/Searching-with-Wildcards-in-EDS-and-EBSCOhost?language=en_US https://support.ebsco.com/help/?int=ehost&lang=en&feature_id=Databases&TOC_ID=Always&SI=0&BU=0&GU=1&PS=0&ver=live&dbs=a9hjnh,a9h
**ROBINS-I**	NA	https://www.riskofbias.info/welcome/home/current-version-of-robins-i/robins-i-tool-2016
**Protocol Repository**	NA	https://osf.io/tzcxq

Supplementary File S2
Template for task tracking, searches, screening, data collection and assessment forms

	A	B	C	D	E	F	G	H	I
1	**Number**	**SearchType**	**Database**	**Collections**	**All**	**Filter**	**Query**	**Results_R?_T?**	**Date_R?_T?**
2	#1(Population)	Articles	Pubmed	Pubmed	Title	No	(soccer[Title] OR football[Title]) AND (elite*[Title] OR professional*[Title] OR association[Title]) NOT "Australian Rules Football"[Title] NOT "Australian Football League"[Title] NOT "American Football"[Title] NOT "National Football League"[Title] NOT "Gaelic Football"[Title] NOT rugby[Title] NOT basketball[Title] NOT handball[Title] NOT volleyball[Title] NOT indoor[Title] NOT former[Title] NOT retired*[Title] NOT referee*[Title] NOT amateur*[Title] NOT academ*[Title] NOT youth[Title] NOT junior*[Title] NOT young*[Title] NOT colleg*[Title] NOT adolescent*[Title] NOT universit*[Title] NOT under-1?[Title] NOT female*[Title] NOT wom?n[Title]	?	?
3	#2(Intervention, Comparison)	Articles	Pubmed	Pubmed	Title-Abstract-Keywords	No	(intervention*[Title/Abstract] OR decision*[Title/Abstract] OR instruction*[Title/Abstract] OR formation*[Title/Abstract] OR strateg*[Title/Abstract] OR substitut*[Title/Abstract] OR program*[Title/Abstract] OR change*[Title/Abstract] OR constraint*[Title/Abstract] OR method*[Title/Abstract] OR practice*[Title/Abstract] OR training[Title/Abstract] OR coach*[Title/Abstract] OR adjust*[Title/Abstract] OR condition*[Title/Abstract] OR protocol*[Title/Abstract] OR load*[Title/Abstract] OR warm-up*[Title/Abstract] OR exercise*[Title/Abstract] OR position*[Title/Abstract] OR prevention*[Title/Abstract] OR preparation*[Title/Abstract] OR context*[Title/Abstract] OR situation*[Title/Abstract] OR half*[Title/Abstract] OR halves[Title/Abstract] OR match*[Title/Abstract] OR game*[Title/Abstract])	?	?
4	#3(Outcome)	Articles	Pubmed	Pubmed	Title-Abstract-Keywords	No	(outcome*[Title/Abstract] OR winn*[Title/Abstract] OR win[Title/Abstract] OR won[Title/Abstract] OR lose[Title/Abstract] OR loss[Title/Abstract] OR losing[Title/Abstract] OR victor*[Title/Abstract] OR odds[Title/Abstract] OR expect*[Title/Abstract] OR probabili*[Title/Abstract] OR result*[Title/Abstract] OR success[Title/Abstract] OR discriminat*[Title/Abstract] OR score*[Title/Abstract] OR action*[Title/Abstract] OR metric*[Title/Abstract] OR indicator*[Title/Abstract] OR statistic*[Title/Abstract] OR factor*[Title/Abstract] OR rank*[Title/Abstract] OR stand*[Title/Abstract] OR goal*[Title/Abstract] OR points[Title/Abstract] OR performance*[Title/Abstract] OR effect*[Title/Abstract])	?	?
5	#4(Study Design)	Articles	Pubmed	Pubmed	Title-Abstract-Keywords	No	(("notational analysis"[Title/Abstract] OR "performance analysis"[Title/Abstract] OR "match analysis"[Title/Abstract] OR "game analysis"[Title/Abstract] OR observation*[Title/Abstract] OR cross-sectional[Title/Abstract] OR cohort[Title/Abstract] OR case-control[Title/Abstract] OR longitudinal*[Title/Abstract] OR analytics[Title/Abstract] OR "machine learning"[Title/Abstract] OR predict*[Title/Abstract] OR classif*[Title/Abstract]) NOT review[Title/Abstract] NOT "meta-analysis"[Title/Abstract] NOT synthesis[Title/Abstract] NOT experimental[Title/Abstract])	?	?
6	#5	Articles	Pubmed	Pubmed	Combined	No	#1 AND #2 AND #3 AND #4	?	?
7	#6	Systematic Review	Pubmed	Pubmed	Title-Abstract-Keywords	No	(“systematic review”[Title/Abstract] OR “meta-analysis”[Title/Abstract] OR “synthesis”[Title/Abstract])	?	?
8	#7	Systematic Review	Pubmed	Pubmed	Combined	No	#1 AND #2 AND #3 AND #6	?	?
9	#1(Population)	Articles	Scopus	Scopus	Title	No	TITLE((soccer OR football) AND (elite* OR professional* OR association) AND NOT "Australian Rules Football" AND NOT “Australian Football League” AND NOT “American Football” AND NOT "National Football League" AND NOT "Gaelic Football" AND NOT rugby AND NOT basketball AND NOT handball AND NOT volleyball AND NOT indoor AND NOT former AND NOT retired* AND NOT referee* AND NOT amateur* AND NOT academ* AND NOT youth AND NOT junior* AND NOT young* AND NOT colleg* AND NOT adolescent* AND NOT universit* AND NOT under-1? AND NOT female* AND NOT wom?n)	?	?
10	#2(Intervention, Comparison)	Articles	Scopus	Scopus	Title-Abstract-Keywords	No	TITLE-ABS-KEY(intervention* OR decision* OR instruction* OR formation* OR strateg* OR substitut* OR program* OR change* OR constraint* OR method* OR practice* OR training OR coach* OR adjust* OR condition* OR protocol* OR load* OR warm-up* OR exercise* OR position* OR prevention* OR preparation* OR context* OR situation* OR half* OR halves OR match* OR game*)	?	?
11	#3(Outcome)	Articles	Scopus	Scopus	Title-Abstract-Keywords	No	TITLE-ABS-KEY(outcome* OR winn* OR win OR won OR lose OR loss OR losing OR victor* OR odds OR expect* OR probabili* OR result* OR success OR discriminat* OR score* OR action* OR metric* OR indicator* OR statistic* OR factor* OR rank* OR stand* OR goal* OR points OR performance* OR effect*)	?	?
12	#4(Study Design)	Articles	Scopus	Scopus	Title-Abstract-Keywords	No	TITLE-ABS-KEY(("notational analysis" OR "performance analysis" OR “match analysis” OR "game analysis" OR observation* OR cross-sectional OR cohort OR case-control OR longitudinal* OR analytics OR “machine learning” OR predict* OR classif*) AND NOT review AND NOT “meta-analysis” AND NOT synthesis AND NOT experimental)	?	?
13	#5	Articles	Scopus	Scopus	Combined	No	#1 AND #2 AND #3 AND #4	?	?
14	#6	Systematic Review	Scopus	Scopus	Title-Abstract-Keywords	No	TITLE-ABS-KEY(“systematic review” OR “meta-analysis” OR “synthesis”)	?	?
15	#7	Systematic Review	Scopus	Scopus	Combined	No	#1 AND #2 AND #3 AND #6	?	?
16	#1(Population)	Articles	Web of Science	Web of Science Core Collection;Current Contents Connect;Derwent Innovations Index;Grants Index;KCI-Korean Journal Database;MEDLINE;ProQuest;SciELO	Title	No	TI=((soccer OR football) AND (elite* OR professional* OR association) NOT "Australian Rules Football" NOT “Australian Football League” NOT “American Football” NOT "National Football League" NOT "Gaelic Football" NOT rugby NOT basketball NOT handball NOT volleyball NOT indoor NOT former NOT retired* NOT referee* NOT amateur* NOT academ* NOT youth NOT junior* NOT young* NOT colleg* NOT adolescent* NOT universit* NOT under-1? NOT female* NOT wom?n)	?	?

Supplementary File S2
Template for task tracking, searches, screening, data collection and assessment forms

	A	B	C	D	E	F	G	H	I
1	**Number**	**SearchType**	**Database**	**Collections**	**All**	**Filter**	**Query**	**Results_R?_T?**	**Date_R?_T?**
17	#2(Intervention, Comparison)	Articles	Web of Science	Web of Science Core Collection;Current Contents Connect;Derwent Innovations Index;Grants Index;KCI-Korean Journal Database;MEDLINE;ProQuest;SciELO	Title-Abstract-Keywords	No	TS=((intervention* OR decision* OR instruction* OR formation* OR strateg* OR substitut* OR program* OR change* OR constraint* OR method* OR practice* OR training OR coach* OR adjust* OR condition* OR protocol* OR load* OR warm-up* OR exercise* OR position* OR prevention* OR preparation* OR context* OR situation* OR half* OR halves OR match* OR game*))	?	?
18	#3(Outcome)	Articles	Web of Science	Web of Science Core Collection;Current Contents Connect;Derwent Innovations Index;Grants Index;KCI-Korean Journal Database;MEDLINE;ProQuest;SciELO	Title-Abstract-Keywords	No	TS=((outcome* OR winn* OR win OR won OR lose OR loss OR losing OR victor* OR odds OR expect* OR probabili* OR result* OR success OR discriminat* OR score* OR action* OR metric* OR indicator* OR statistic* OR factor* OR rank* OR stand* OR goal* OR points OR performance* OR effect*))	?	?
19	#4(Study Design)	Articles	Web of Science	Web of Science Core Collection;Current Contents Connect;Derwent Innovations Index;Grants Index;KCI-Korean Journal Database;MEDLINE;ProQuest;SciELO	Title-Abstract-Keywords	No	TS=(("notational analysis" OR "performance analysis" OR “match analysis” OR "game analysis" OR observation* OR cross-sectional OR cohort OR case-control OR longitudinal* OR analytics OR “machine learning” OR predict* OR classif*) NOT review NOT “meta-analysis” NOT synthesis NOT experimental)	?	?
20	#5	Articles	Web of Science	Web of Science Core Collection;Current Contents Connect;Derwent Innovations Index;Grants Index;KCI-Korean Journal Database;MEDLINE;ProQuest;SciELO	Combined	No	#1 AND #2 AND #3 AND #4	?	?
21	#6	Systematic Review	Web of Science	Web of Science Core Collection;Current Contents Connect;Derwent Innovations Index;Grants Index;KCI-Korean Journal Database;MEDLINE;ProQuest;SciELO	Title-Abstract-Keywords	No	TS=(“systematic review” OR “meta-analysis” OR “synthesis”)	?	?
22	#7	Systematic Review	Web of Science	Web of Science Core Collection;Current Contents Connect;Derwent Innovations Index;Grants Index;KCI-Korean Journal Database;MEDLINE;ProQuest;SciELO	Combined	No	#1 AND #2 AND #3 AND #6	?	?
23	S1(Population)	Articles	EBSCOhost	MLA International Bibliography with Full Text, Library, Information Science & Technology Abstracts, CINAHL Plus, GreenFILE, Teacher Reference Center, eBook Collection (EBSCOhost), MathSciNet via EBSCOhost, MLA Directory of Periodicals, PSICODOC, eBook Open Access (OA) Collection (EBSCOhost)	Title	No	TI((soccer OR football) AND (elite* OR professional* OR association) NOT "Australian Rules Football" NOT “Australian Football League” NOT “American Football” NOT "National Football League" NOT "Gaelic Football" NOT rugby NOT basketball NOT handball NOT volleyball NOT indoor NOT former NOT retired* NOT referee* NOT amateur* NOT academ* NOT youth NOT junior* NOT young* NOT colleg* NOT adolescent* NOT universit* NOT under-1? NOT female* NOT wom?n)	?	?
24	S2(Intervention, Comparison)	Articles	EBSCOhost	MLA International Bibliography with Full Text, Library, Information Science & Technology Abstracts, CINAHL Plus, GreenFILE, Teacher Reference Center, eBook Collection (EBSCOhost), MathSciNet via EBSCOhost, MLA Directory of Periodicals, PSICODOC, eBook Open Access (OA) Collection (EBSCOhost)	Title-Abstract-Keywords	No	intervention* OR decision* OR instruction* OR formation* OR strateg* OR substitut* OR program* OR change* OR constraint* OR method* OR practice* OR training OR coach* OR adjust* OR condition* OR protocol* OR load* OR warm-up* OR exercise* OR position* OR prevention* OR preparation* OR context* OR situation* OR half* OR halves OR match* OR game*	?	?
25	S3(Outcome)	Articles	EBSCOhost	MLA International Bibliography with Full Text, Library, Information Science & Technology Abstracts, CINAHL Plus, GreenFILE, Teacher Reference Center, eBook Collection (EBSCOhost), MathSciNet via EBSCOhost, MLA Directory of Periodicals, PSICODOC, eBook Open Access (OA) Collection (EBSCOhost)	Title-Abstract-Keywords	No	outcome* OR winn* OR win OR won OR lose OR loss OR losing OR victor* OR odds OR expect* OR probabili* OR result* OR success OR discriminat* OR score* OR action* OR metric* OR indicator* OR statistic* OR factor* OR rank* OR stand* OR goal* OR points OR performance* OR effect*	?	?
26	S4(Study Design)	Articles	EBSCOhost	MLA International Bibliography with Full Text, Library, Information Science & Technology Abstracts, CINAHL Plus, GreenFILE, Teacher Reference Center, eBook Collection (EBSCOhost), MathSciNet via EBSCOhost, MLA Directory of Periodicals, PSICODOC, eBook Open Access (OA) Collection (EBSCOhost)	Title-Abstract-Keywords	No	("notational analysis" OR "performance analysis" OR “match analysis” OR "game analysis" OR observation* OR cross-sectional OR cohort OR case-control OR longitudinal* OR analytics OR “machine learning” OR predict* OR classif*) NOT review NOT “meta-analysis” NOT synthesis NOT experimental)	?	?
27	S5	Articles	EBSCOhost	MLA International Bibliography with Full Text, Library, Information Science & Technology Abstracts, CINAHL Plus, GreenFILE, Teacher Reference Center, eBook Collection (EBSCOhost), MathSciNet via EBSCOhost, MLA Directory of Periodicals, PSICODOC, eBook Open Access (OA) Collection (EBSCOhost)	Combined	No	S1 AND S2 AND S3 AND S4	?	?

	A	B	C	D	E	F	G	H	I
1	**Number**	**SearchType**	**Database**	**Collections**	**All**	**Filter**	**Query**	**Results_R?_T?**	**Date_R?_T?**
28	S5	Articles	EBSCOhost	MLA International Bibliography with Full Text, Library, Information Science & Technology Abstracts, CINAHL Plus, GreenFILE, Teacher Reference Center, eBook Collection (EBSCOhost), MathSciNet via EBSCOhost, MLA Directory of Periodicals, PSICODOC, eBook Open Access (OA) Collection (EBSCOhost)	Combined	Yes	S1 AND S2 AND S3 AND S4	?	?
29	S6	Systematic Review	EBSCOhost	MLA International Bibliography with Full Text, Library, Information Science & Technology Abstracts, CINAHL Plus, GreenFILE, Teacher Reference Center, eBook Collection (EBSCOhost), MathSciNet via EBSCOhost, MLA Directory of Periodicals, PSICODOC, eBook Open Access (OA) Collection (EBSCOhost)	Title-Abstract-Keywords	No	“systematic review” OR “meta-analysis” OR “synthesis”	?	?
30	S7	Systematic Review	EBSCOhost	MLA International Bibliography with Full Text, Library, Information Science & Technology Abstracts, CINAHL Plus, GreenFILE, Teacher Reference Center, eBook Collection (EBSCOhost), MathSciNet via EBSCOhost, MLA Directory of Periodicals, PSICODOC, eBook Open Access (OA) Collection (EBSCOhost)	Combined	No	S1 AND S2 AND S3 AND S6	?	?
31	S7	Systematic Review	EBSCOhost	MLA International Bibliography with Full Text, Library, Information Science & Technology Abstracts, CINAHL Plus, GreenFILE, Teacher Reference Center, eBook Collection (EBSCOhost), MathSciNet via EBSCOhost, MLA Directory of Periodicals, PSICODOC, eBook Open Access (OA) Collection (EBSCOhost)	Combined	Yes	S1 AND S2 AND S3 AND S6	?	?
32								?	?

Supplementary File S2
Template for task tracking, searches, screening, data collection and assessment form

	A	B	C	D	E	F	G	H	I	J	K	L	M	N	O
1	**Number**	**SearchType**	**SourceID**	**PubmedID**	**Title**	**Abstract**	**Authors**	**Journal**	**Year**	**DOI**	**DocumentType**	**Source**	**SpecificDatabase**	**SearchNumber**	**Date**
2	?	?	?	?	?	?	?	?	?	?	?	?	?	?	?
3	?	?	?	?	?	?	?	?	?	?	?	?	?	?	?

	A	B	C	D	E	F	G	H	I
1	**ID**	**SearchType**	**Title**	**Pubmed_R?_T?**	**Scopus_R?_T?**	**WoS_R?_T?**	**Ebscohost_R?_T?**	**Search_date_R?_T?**	**Included_Citations_R?_T?**
2	?	?	?	?	?	?	?	?	?
3	?	?	?	?	?	?	?	?	?

Supplementary File S2
Template for task tracking, searches, screening, data collection and assessment forms

	A	B	C	D	E	F	G	H	I
1	**ID**	**SearchType**	**Title**	**T_Peer-Reviewed_R?_T?**	**T_IC1_R?_T?**	**T_IC2_R?_T?**	**T_IC3_R?_T?**	**T_IC4_R?_T?**	**T_IC5_R?_T?**
2	?	?	?	?	?	?	?	?	?
3	?	?	?	?	?	?	?	?	?

	J	K	L	M	N	O	P
1	**T_IC6_R?_T?**	**T_IC7_R?_T?**	**T_IC8_R?_T?**	**T_IC9_R?_T?**	**T_Result_R?_T?**	**T_First_Reason_Exclusion_R?_T?**	**T_Justification_R?_T?**
2	?	?	?	?			?
3	?	?	?	?			?

Cell: E1
Note: 1-The research application area is football or soccer. (P)

Cell: F1
Note: 2-The study design is an observational type. (S)

Cell: G1
Note: 3-The sample of the study is adults.(P)

Cell: H1
Note: 4-The sample's genre is described as male. (P)

Cell: I1
Note: 5-The sample is elite or world-class.(P)

Cell: J1
Note: 6-The unit of analysis is teams or team observations (P)

Cell: K1
Note: 7-The data is collected from official competitive matches (P)

Cell: L1
Note: 8-The research question includes team match interventions as an independent or predictor variable.(I, C)

Cell: M1
Note: 9-The research question includes a match performance or a competition success measure as a dependent or outcome variable. (O)

Supplementary File S2
Template for task tracking, searches, screening, data collection and assessment forms

	A	B	C	D	E	F	G	H	I	J
1	**ID**	**SearchType**	**Abstract**	**A_Peer-Reviewed_R?_T?**	**A_IC1_R?_T?**	**A_IC2_R?_T?**	**A_IC3_R?_T?**	**A_IC4_R?_T?**	**A_IC5_R?_T?**	**A_IC6_R?_T?**
2	?	?	?	?	?	?	?	?	?	?
3	?	?	?	?	?	?	?	?	?	?

	K	L	M	N	O	P
1	**A_IC7_R?_T?**	**A_IC8_R?_T?**	**A_IC9_R?_T?**	**A_Result_R?_T?**	**A_First_Reason_Exclusion_R?_T?**	**A_Justification_R?_T?**
2	?	?	?			?
3	?	?	?			?

Cell: E1
Note: 1-The research application area is football or soccer. (P)

Cell: F1
Note: 2-The study design is an observational type. (S)

Cell: G1
Note: 3-The sample of the study is adults.(P)

Cell: H1
Note: 4-The sample's genre is described as male. (P)

Cell: I1
Note: 5-The sample is elite or world-class.(P)

Cell: J1
Note: 6-The unit of analysis is teams or team observations (P)

Cell: K1
Note: 7-The data is collected from official competitive matches (P)

Cell: L1
Note: 8-The research question includes team match interventions as an independent or predictor variable.(I, C)

Cell: M1
Note: 9-The research question includes a match performance or a competition success measure as a dependent or outcome variable. (O)

	A	B	C	D	E	F	G	H	I
1	**ID**	**SearchType**	**Full-text**	**F_Peer-Reviewed_R?_T?**	**F_IC1_R?_T?**	**F_IC2_R?_T?**	**F_IC3_R?_T?**	**F_IC4_R?_T?**	**F_IC5_R?_T?**
2	?	?	?	?	?	?	?	?	?
3	?	?	?	?	?	?	?	?	?

	J	K	L	M	N	O	P
1	**F_IC6_R?_T?**	**F_IC7_R?_T?**	**F_IC8_R?_T?**	**F_IC9_R?_T?**	**F_Result_R?_T?**	**F_First_Reason_Exclusion_R?_T?**	**F_Justification_R?_T?**
2	?	?	?	?			?
3	?	?	?	?			?

Cell: E1
Note: 1-The research application area is football or soccer. (P)

Cell: F1
Note: 2-The study design is an observational type. (S)

Cell: G1
Note: 3-The sample of the study is adults.(P)

Cell: H1
Note: 4-The sample's genre is described as male. (P)

Cell: I1
Note: 5-The sample is elite or world-class.(P)

Cell: J1
Note: 6-The unit of analysis is teams or team observations (P)

Cell: K1
Note: 7-The data is collected from official competitive matches (P)

Cell: L1
Note: 8-The research question includes team match interventions as an independent or predictor variable.(I, C)

Cell: M1
Note: 9-The research question includes a match performance or a competition success measure as a dependent or outcome variable. (O)

	A	B	C
1	**ID**	**M_Year_R?_T?**	**M_Journal_R?_T?**
2	?	?	?
3	?	?	?

	A	B	C	D	E
1	**ID**	**P_Country_R?_T?**	**P_Competition_R?_T?**	**P_YearOfCompetition_R?_T?**	**P_NumberOfTeamsMatchObservations_R?_T?**
2	?	?	?	?	?
3	?	?	?	?	?

	F	G	H
1	**P_NumberOfTeamsMatchObservationsExcluded_R?_T?**	**P_ReasonOfExclusion_R?_T?**	**P_NumberOfTeams_R?_T?**
2	?	?	?
3	?	?	?

	I	J
1	**P_NumberOfPlayers_R?_T?**	**P_NumberOfPlayersObservations_R?_T?**
2	?	?
3	?	?

Cell: A1

Note: - Multiple the ids using rows as many as competitions, seasons and others appear in the study.

Supplementary File S2
Template for task tracking, searches, screening, data collection and assessment forms

	A	B	C	D	E	F	G
1	**ID**	**I_Theme_R?_T?**	**I_Term_R?_T?**	**I_Definition_R?_T?**	**I_Type_R?_T?**	**I_Method_R?_T?**	**I_Frequency_R?_T?**
2	?	?	?	?	?	?	?
3	?	?	?	?	?	?	?

	H	I	J	K	L
1	**I_Duration_R?_T?**	**I_Intensity_R?_T?**	**I_MatchHalves_R?_T?**	**I_MatchPeriod_R?_T?**	**I_PhaseOfTheGame_R?_T?**
2	?	?	?	?	?
3	?	?	?	?	?

Cell: A1

Note: - Multiple the ids using rows as many as interventions appear in the study.

	A	B	C	D	E	F
1	**ID**	**O_Term_R?_T?**	**O_Definition_R?_T?**	**O_Unit_R?_T?**	**O_Type_R?_T?**	**O_PhaseOfTheGame_R?_T?**
2	?		?	?	?	?
3	?		?	?	?	?

Cell: A1

Note: - Multiple the ids using rows as many as outcomes appear in the study.

	A	B	C	D	E
1	**ID**	**C_Intervention1__R?_T?**	**C_Intervention2__R?_T?**	**C_Outcome_R?_T?**	**C_Subgroup_R?_T?**
2	?	?	?	?	?
3	?	?	?	?	?

Supplementary File S2
Template for task tracking, searches, screening, data collection and assessment forms

	F	G	H	I	J
1	**C_ControlVariables_R?_T?**	**C_Covariates_R?_T?**	**C_Mean1_R?_T?**	**C_SD1_R?_T?**	**C_Size1_R?_T?**
2	?	?	?	?	?
3	?	?	?	?	?

	K	L	M	N	O	P
1	**C_Proportion1_R?_T?**	**C_Total1_R?_T?**	**C_Mean2_R?_T?**	**C_SD2_R?_T?**	**C_Size2_R?_T?**	**C_Proportion2_R?_T?**
2	?	?	?	?	?	?
3	?	?	?	?	?	?

	Q	R	S	T	U
1	**C_Total2_R?_T?**	**C_ESValue_R?_T?**	**C_Lower95CI_R?_T?**	**C_Upper95CI_R?_T?**	**C_SE_R?_T?**
2	?	?	?	?	?
3	?	?	?	?	?

	V	W
1	**C_ReportedESFromStudy_R?_T?**	**C_TypeOfESReported_R?_T?**
2	?	?
3	?	?

Cell: A1
Note: - Multiple the ids using rows as many as interventions comparisons to same and different outcomes and subgroups appear in the study.

Supplementary File S2
Template for task tracking, searches, screening, data collection and assessment forms

	A	B	C	D	E
1	**ID**	**S_StudyDesignType_R?_T?**	**S_InstrumentName_R?_T?**	**S_InstrumentType_R?_T?**	**S_InstrumentValidity_R?_T?**
2	?	?	?	?	?
3	?	?	?	?	?

	F	G	H
1	**S_InstrumentInterRealibility_R?_T?**	**S_InstrumentIntraRealibility_R?_T?**	**S_DataAnalysisApproaches_R?_T?**
2	?	?	?
3	?	?	?

	I	J	K
1	**S_InferentialParadigm_R?_T?**	**S_SMLTName_R?_T?**	**S_PreProcessingDataCleaningDescription_R?_T?**
2	?	?	
3	?	?	

	L	M	N	O
1	**S_ModelEvaluationDescription_R?_T?**	**S_MLProblemType_R?_T?**	**S_SMLTAnalysisType_R?_T?**	**S_SMLTMethods_R?_T?**
2		?	?	?
3		?	?	?

Cell: A1

Note: - Multiple the ids using rows as many as tests, instruments and others appear in the study.

Supplementary File S2
Template for task tracking, searches, screening, data collection and assessment forms

	A	B	C	D	E	F	G
1	**ID**	**RoBi_1.1_R?_T?**	**RoBi_1.2_R?_T?**	**RoBi_1.3_R?_T?**	**RoBi_1.4_R?_T?**	**RoBi_1.5_R?_T?**	**RoBi_1.6_R?_T?**
2		?	?	?	?	?	?
3		?	?	?	?	?	?

Supplementary File S2
Template for task tracking, searches, screening, data collection and assessment forms

	H	I	J	K	L	M
1	**RoBi_1.7_R?_T?**	**RoBi_1.8_R?_T?**	**RoBi_1_R?_T?**	**RoBi_2.1_R?_T?**	**RoBi_2.2_R?_T?**	**RoBi_2.3._R?_T?**
2	?	?	?	?	?	?
3	?	?	?	?	?	?

Supplementary File S2
Template for task tracking, searches, screening, data collection and assessment forms

	N	O	P	Q	R	S
1	**RoBi_2.4._R?_T?**	**RoBi_2.5._R?_T?**	**RoBi_2_R?_T?**	**RoBi_3.1_R?_T?**	**RoBi_3.2_R?_T?**	**RoBi_3.3._R?_T?**
2	?	?	?	?	?	?
3	?	?	?	?	?	?

Supplementary File S2
Template for task tracking, searches, screening, data collection and assessment forms

	T	U	V	W	X	Y
1	**RoBi_3_R?_T?**	**RoBi_4.1._R?_T?**	**RoBi_4.2._R?_T?**	**RoBi_4.3._R?_T?**	**RoBi_4.4._R?_T?**	**RoBi_4.5._R?_T?**
2	?	?	?	?	?	?
3	?	?	?	?	?	?

	Z	AA	AB	AC	AD	AE
1	**RoBi_4.6._R?_T?**	**RoBi_4_R?_T?**	**RoBi_5.1._R?_T?**	**RoBi_5.2._R?_T?**	**RoBi_5.3._R?_T?**	**RoBi_5.4._R?_T?**
2	?	?	?	?	?	?
3	?	?	?	?	?	?

	AF	AG	AH	AI	AJ	AK
1	**RoBi_5.5._R?_T?**	**RoBi_5_R?_T?**	**RoBi_6.1._R?_T?**	**RoBi_6.2._R?_T?**	**RoBi_6.3._R?_T?**	**RoBi_6.4._R?_T?**
2	?	?	?	?	?	?
3	?	?	?	?	?	?

	AL	AM	AN	AO	AP	AQ
1	**RoBi_6_R?_T?**	**RoBi_7.1._R?_T?**	**RoBi_7.2._R?_T?**	**RoBi_7.3._R?_T?**	**RoBi_7_R?_T?**	**RoBi_Overall_R?_T?**
2	?	?	?	?	?	?
3	?	?	?	?	?	?

Cell: B1
Note: Is there potential for confounding of the effect of intervention in this study?

Cell: C1
Note: Was the analysis based on splitting participants' follow up time according to intervention received?

Cell: D1
Note: Were intervention discontinuations or switches likely to be related to factors that are prognostic for the outcome?

Cell: E1
Note: Did the authors use an appropriate analysis method that controlled for all the important confounding domains?

Cell: F1
Note: If Y/PY to 1.4: Were confounding domains that were controlled for measured validly and reliably by the variables available in this study?

Cell: G1
Note: Did the authors control for any post-intervention variables that could have been affected by the intervention?

Cell: H1
Note: Did the authors use an appropriate analysis method that adjusted for all the important confounding domains and for time-varying confounding?

Cell: I1
Note: If Y/PY to 1.7: Were confounding domains that were adjusted for measured validly and reliably by the variables available in this study?

Cell: J1
Note: Bias due to confounding

Cell: K1
Note: Was selection of participants into the study (or into the analysis) based on participant characteristics observed after the start of intervention?

Cell: L1
Note: Were the post-intervention variables that influenced selection likely?

Cell: M1
Note: If Y/PY to 2.2: Were the post-intervention variables that influenced selection likely to be influenced by the outcome or a cause of the outcome?

Cell: N1
Note: Do start of follow-up and start of intervention coincide for most participants?

Cell: O1
Note: If Y/PY to 2.2 and 2.3, or N/PN to 2.4: Were adjustment techniques used that are likely to correct for the presence of selection biases?

Cell: P1
Note: Bias in selection of participants into the study

Cell: Q1
Note: Were intervention groups clearly defined?

Cell: R1
Note: Was the information used to define intervention groups recorded at the start of the intervention?

Cell: S1
Note: Could classification of intervention status have been affected by knowledge of the outcome or risk of the outcome?

Cell: T1
Note: Bias in classification of interventions

Cell: U1
Note: Were there deviations from the intended intervention beyond what would be expected in usual practice?

Cell: V1
Note: If Y/PY to 4.1: Were these deviations from intended intervention unbalanced between groups and likely to have affected the outcome?

Cell: W1
Note: Were important co-interventions balanced across intervention groups?

Cell: X1
Note: Was the intervention implemented successfully for most participants?

Cell: Y1
Note: Did study participants adhere to the assigned intervention regimen?

Cell: Z1
Note: If N/PN to 4.3, 4.4 or 4.5: Was an appropriate analysis used to estimate the effect of starting and adhering to the intervention?

Cell: AA1
Note: Bias due to deviations from intended interventions

Cell: AB1
Note: Were outcome data available for all, or nearly all, participants?

Cell: AC1
Note: Were participants excluded due to missing data on intervention status?

Cell: AD1
Note: Were participants excluded due to missing data on other variables needed for the analysis?

Cell: AE1
Note: If PN/N to 5.1, or Y/PY to 5.2 or 5.3: Are the proportion of participants and reasons for missing data similar across interventions?

Cell: AF1
Note: If PN/N to 5.1, or Y/PY to 5.2 or 5.3: Is there evidence that results were robust to the presence of missing data?

Cell: AG1
Note: Bias due to missing data

Cell: AH1
Note: Could the outcome measure have been influenced by knowledge of the intervention received?

Cell: AI1
Note: Were outcome assessors aware of the intervention received by study participants?

Cell: AJ1
Note: Were the methods of outcome assessment comparable across intervention groups?

Cell: AK1
Note: Were any systematic errors in measurement of the outcome related to intervention received?

Cell: AL1
Note: Bias in measurement of outcomes

Cell: AM1
Note: Is the reported effect estimate likely to be selected, on the basis of the results, from multiple outcome measurements within the outcome domain?

Cell: AN1
Note: Is the reported effect estimate likely to be selected, on the basis of the results, from multiple analyses of the intervention-outcome relationship?

Cell: AO1
Note: Is the reported effect estimate likely to be selected, on the basis of the results, from different subgroups?

Cell: AP1
Note: Bias in selection of the reported outcomes

Cell: AQ1
Note: Overall

Supplementary File S2
Template for task tracking, searches, screening, data collection and assessment forms

Abbreviation	Description
A	Abstract screening
C	Comparisons
CI	Confidence intervals
ES	Effect Size
F	Full-text screening
GS	Gold Standard
I	Intervention
IC1	1-The research application area is football or soccer. (P)
IC2	2-The study design is an observational type. (S)
IC3	3-The sample of the study is adults. (P)
IC4	4-The sample's genre is described as male. (P)
IC5	5-The sample is elite or world-class. (P)
IC6	6- The unit of analysis is teams or team observations (P)
IC7	7- The data is collected from official competitive matches (P)
IC8	8-The research question includes team match interventions as an independent or predictor variable. (I,C)
IC9	9-The research question includes a match performance or a competition success measure as a dependent or outcome variable. (O)
M	Metadata
ML	Machine Learning
SMLT	Statistical/Machine Learning Tests/Techniques
NA	Not applicable
NI	No information
O	Outcome
P	Population
R?_T?	Reviewer number and times of collection, e.g., R1_T1 means Reviewer 1 Time Point 1
RoBi_1.0.	Bias due to confounding
RoBi_1.1.	Is there potential for confounding of the effect of intervention in this study?
RoBi_1.2.	Was the analysis based on splitting participants' follow up time according to intervention received?
RoBi_1.3.	Were intervention discontinuations or switches likely to be related to factors that are prognostic for the outcome?
RoBi_1.4.	Did the authors use an appropriate analysis method that controlled for all the important confounding domains?
RoBi_1.5.	If Y/PY to 1.4: Were confounding domains that were controlled for measured validly and reliably by the variables available in this study?
RoBi_1.6.	Did the authors control for any post-intervention variables that could have been affected by the intervention?
RoBi_1.7.	Did the authors use an appropriate analysis method that adjusted for all the important confounding domains and for time-varying confounding?
RoBi_1.8.	If Y/PY to 1.7: Were confounding domains that were adjusted for measured validly and reliably by the variables available in this study?
RoBi_2.0.	Bias in selection of participants into the study
RoBi_2.1.	Was selection of participants into the study (or into the analysis) based on participant characteristics observed after the start of intervention?
RoBi_2.2.	Were the post-intervention variables that influenced selection likely?
RoBi_2.3.	If Y/PY to 2.2: Were the post-intervention variables that influenced selection likely to be influenced by the outcome or a cause of the outcome?
RoBi_2.4.	Do start of follow-up and start of intervention coincide for most participants?
RoBi_2.5.	If Y/PY to 2.2 and 2.3, or N/PN to 2.4: Were adjustment techniques used that are likely to correct for the presence of selection biases?
RoBi_3.0.	Bias in classification of interventions
RoBi_3.1.	Were intervention groups clearly defined?
RoBi_3.2.	Was the information used to define intervention groups recorded at the start of the intervention?
RoBi_3.3.	Could classification of intervention status have been affected by knowledge of the outcome or risk of the outcome?
RoBi_4.0.	Bias due to deviations from intended interventions
RoBi_4.1.	Were there deviations from the intended intervention beyond what would be expected in usual practice?
RoBi_4.2.	If Y/PY to 4.1: Were these deviations from intended intervention unbalanced between groups and likely to have affected the outcome?
RoBi_4.3.	Were important co-interventions balanced across intervention groups?
RoBi_4.4.	Was the intervention implemented successfully for most participants?
RoBi_4.5.	Did study participants adhere to the assigned intervention regimen?
RoBi_4.6.	If N/PN to 4.3, 4.4 or 4.5: Was an appropriate analysis used to estimate the effect of starting and adhering to the intervention?
RoBi_5.0.	Bias due to missing data
RoBi_5.1.	Were outcome data available for all, or nearly all, participants?
RoBi_5.2.	Were participants excluded due to missing data on intervention status?
RoBi_5.3.	Were participants excluded due to missing data on other variables needed for the analysis?
RoBi_5.4.	If PN/N to 5.1, or Y/PY to 5.2 or 5.3: Are the proportion of participants and reasons for missing data similar across interventions?
RoBi_5.5.	If PN/N to 5.1, or Y/PY to 5.2 or 5.3: Is there evidence that results were robust to the presence of missing data?
RoBi_6.0.	Bias in measurement of outcomes
RoBi_6.1.	Could the outcome measure have been influenced by knowledge of the intervention received?
RoBi_6.2.	Were outcome assessors aware of the intervention received by study participants?
RoBi_6.3.	Were the methods of outcome assessment comparable across intervention groups?
RoBi_6.4.	Were any systematic errors in measurement of the outcome related to intervention received?
RoBi_7.0.	Bias in selection of the reported outcomes
RoBi_7.1.	Is the reported effect estimate likely to be selected, on the basis of the results, from multiple outcome measurements within the outcome domain?
RoBi_7.2.	Is the reported effect estimate likely to be selected, on the basis of the results, from multiple analyses of the intervention-outcome relationship?
RoBi_7.3.	Is the reported effect estimate likely to be selected, on the basis of the results, from different subgroups?
RoBi_Overall	Overall
S	Study design
SD	Standard Deviation
SE	Standard Error
T	Title screening

Supplementary File S2
Template for task tracking, searches, screening, data collection and assessment forms

yes/no	Result	Inclusion criteria	Exclusion criteria	Status
0-No	0=Excluded	1-The research application area is football or soccer. (P)	1-The research application area is not football or soccer. (P)	Done
1-Yes	1=Included	2-The study design is an observational type. (S)	2-The study design is not an observational type. (S)	In progress
NA	Next stage	3-The sample of the study is adults. (P)	3-The sample of the study is not adults. (P)	To Do
?	NA	4-The sample's genre is described as male. (P)	4-The sample's genre is not described as male. (P)	
	?	5-The sample is elite or world-class. (P)	5-The sample is not elite or world-class. (P)	
		6- The unit of analysis is teams or team observations (P)	6- The unit of analysis is not teams or team observations (P)	
		7- The data is collected from official competitive matches (P)	7- The data is not collected from official competitive matches (P)	
		8-The research question includes team match interventions as an independent or predictor variable. (I,C)	8-The research question does not include team match interventions as an independent or predictor variable. (I,C)	
		9-The research question includes a match performance or a competition success measure as a dependent or outcome variable. (O)	9-The research question does not include a match performance or a competition success measure as a dependent or outcome variable. (O)	
		Not peer-review	Not peer-review	
		Not available	Not available	
		NA	NA	
		?	?	

Supplementary File S2
Template for task tracking, searches, screening, data collection and assessment forms

Source	Robins-1-a	Robins-1-b	Robins-1-c	Robins-final	Intervention method	Intervention Intensity	Period halves	Period
Pubmed	Yes	Yes	Yes	Low	1 = Conditioning and training programs	1 = Low Intensity	1 = Pre-first half	1 = Pre-match
Web of Science	Probable Yes	Probable Yes	Probable Yes	Moderate	2 = Nutritional and hydration strategies	2 = Moderate Intensity	2 = In-first half	2 = In-match
Ebscohost	Probable No	Probable No	Probable No	Serious	3 = Psychological techniques	3 = Vigorous Intensity	3 = Pre-second half	3 = Pos-match
Scopus	No	No	No	Critical	4 = Strategical and tactical decisions	4 = High Intensity	4 = In-second half	NA
Included studies	No information	No information	?	NI	5 = Training periodisation	5 = Submaximal Intensity	NA	?
Systematic reviews	NA	?		?	6 = Injury-related	6 = Maximal Intensity	?	
	?				7 = Equipment-related	NA		
					8 = Other	?		
					NA			
					?			

Supplementary File S2
Template for task tracking, searches, screening, data collection and assessment forms

Phase of the game	Effect size reported	Odds ratio type	Type of outcome	Study design	Instrument type	Instrument validity	Data analysis approaches
1 = Defensive	1 = Odds ratio	1 = Crude	1 = Team success	1 = Case-control	1 = Semi- or full-automatic tracking systems	1 = Not stated	1 = Statistical modelling
2 = Offensive	2 = Log odds ratio	2 = Adjusted	2 = Team match performance	2 = Cross-sectional	2 = Global positions systems	2 = Unclear	2 = Machine learning modelling
3 = Transition attack-defense	3 = Cohen’s d	NA	3 = Team match effects	3 = Cohort	3 = Databases/websites	3 = Stated	NA
4 = Transition defense-attack	4 = Hedges’ g	?	NA	4 = Longitudinal	4 = Notational or observational instruments	NA	?
5 = Set-pieces	5 = Standardised coefficients		?	5 = Other	5 = Survey	?	
6 = Globally	6 = Other			NA	6 = Other		
NA	7=None			?	NA		
?	NA				?		
	?						

Supplementary File S2
Template for task tracking, searches, screening, data collection and assessment forms

Inferential paradigm	Statistical tests analysis type	Statistical tests methods	Intervention Type	Machine Learning Problems	Units	SearchType
1 = Frequentist	1 = Univariate	1 = Differences	1 = Sports Science	1 = Prediction	1 = Count	Articles
2 = Bayesian	2 = Bivariate	2 = Associations and correlations	2 = Sports Medicine	2 = Classification	2 = Meters	Systematic Reviews
NA	3 = Multivariate	3 = Regression	3 = Both	3 = Clustering	3 = Meters/Second	NA
?	NA	NA	4 = Other	4 = Other	4 = Meters/Second2	?
	?	?	NA	NA	5 = Ratio	
			?	?	6 = Percentages	
					7 = Difference	
					8 = Arbitrary Unit	
					9 = Probabilities	
					10 = Other	
					NA	
					NI	
					?	
