# Supplementary material for: Ranking sports science and medicine interventions impacting team performance: a protocol for a systematic review and meta-analysis of observational studies in elite football
Source: BMJ Open Sport Exerc Med. 2024 Sep 13;10(3):e002196. doi: 10.1136/bmjsem-2024-002196 (PMC11404162; doi:10.1136/bmjsem-2024-002196)
Supplement: online supplemental file 3 [file bmjsem-10-3-s003.pdf]

Supplementary File S3:
Instructions for Systematic Reviewers

August 2024

1 Material . . . 2

2 Reviewer Identification . . . 3

3 Training . . . 4

4 Search Task . . . 5

5 Duplicates Task . . . 9

6 Screening Task . . . 10

7 Data Collection Task . . . 12

8 Data Assessment Task . . . 13

9 Cleaning and Checking . . . 14

1 Material

These instructions require the following material:

- Supplementary File S2: Template for task tracking, searches, data collection and assessment forms.
- Supplementary Table S5: Inclusion and exclusion criteria.
- Supplementary Table S8: Data form fields, scale, description and codes.

2 Reviewer Identification

Insert your assigned number and time point into the 'Intro' sheet. In each column header of the first row in each sheet, there is the code 'R?_T?'. Replace the first '?' character with your assignment number and the second '?' character with the time point number of the task.

For example: if you are assigned as Reviewer 2 and it is your first time doing the task, then the column header 'R?_T?' would be 'R2_T1'.

You can use the replace option in Excel:

1. Press Ctrl+F to open 'Find and Replace'.
2. Select 'Replace' and write 'R?T?' in 'Find what' and 'R1T1' in 'Replace with' if you are Reviewer 1 and it is your first time Doing the tasks.
3. Select the 'Workbook' option in the 'Within' drop-down menu and click 'Replace All'.

For all following tasks, your goal is to replace all cells containing '?' with the real data.

Important: Please, save the file with name 'Supplementary File S2' as 'R?_T?', and substitute '?' as appropriate.

3 Training

1. A training procedure will precede each subsequent task. In the first step of this training process, familiarise yourself with the provided documentation and instructions during online meetings.

2. Next, assess the records or reports or other materials, e.g., queries related to the topic provided to you.

3. After completing your assessments, submit them to the first and second authors of the gold standard process. They will provide feedback on your evaluations, helping you refine your understanding and application of the criteria.

4. After reviewing your feedback, the authors will determine if you are ready to proceed with the task.

4 Search Task

1. Complete the required training, including familiarisation with documentation and search of a query in each database before proceeding with this task.

2. Connect to the virtual private network (VPN) of the University of Lleida. Note: It is assumed that VPN configurations are already set. If not, please consult the relevant documentation or support at `https://bid.udl.cat/ca/ajuda/com-accedir-als-recursos-electronics-des-de-fora-de-la-udl/`.

3. Access the PubMed electronic database: `https://pubmed.ncbi.nlm.nih.gov/advanced/`.

4. Copy query number #1 from the '1SearchesHistory' sheet of Supplementary File S2 into the PubMed query box, and click 'Add to history' in the search drop-down menu.

5. In '1SearchesHistory' sheet of Supplementary File S2, enter the number and the date into the corresponding cells under the headers 'Results' and 'Date'.

6. Repeat steps 4 to 5 for the remaining queries of articles 'SearchType' option on PubMed.

7. Click on 'Download' to save the history file locally. Save the file with the name in all lowercase in the following format:

   - {database_name}_{task}_history_{n}of{N}_R{?}T{?}_{Date}

   Here, 'database_name' is the name of the database, 'task' is the name of the current task, 'n' is the current page number, 'N' is the total number of pages, '?' represents the reviewer ID and time point, and 'Date' is the date. Example: pubmed_search_history_1of1_R1T1_01-07-2024.

8. Click on the number below the header Results to access the records, then click the Save option. In the Selection dropdown, choose All results on this page. For Format, select CSV and click 'Create file'. Save the file with the name in all lowercase in the following format:

   - {database_name}_{task}_results_{n}of{N}_R{?}T{?}_{Date}

   Here, 'database_name' is the name of the database, 'task' is the name of the current task, 'n' is the current page number, 'N' is the total number of pages, '?' represents the reviewer ID and time point, and 'Date' is the date. Example: pubmed_search_results_1of1_R1T1_01-07-2024. Do the same for 'nbib' using the option 'Send to', citation manager, and select 'All' to download the file so the software can import the records.

9. Repeat steps 4 to 8 for the systematic review 'SearchType' option queries.

10. Access the Scopus electronic database: `https://www.scopus.com/search/form.uri?display=advanced`, and repeat steps 4-6 for articles 'SearchType' option queries. Insert queries 1-4 in 'Advanced Search', select the number of queries, and combine them in 'Basic Search' for the 5.

11. There is no option to download the history in Scopus as Excel, so go to 'Basic Search', right-click, print, and select 'Save as PDF'. Save in the same format as in step 7.

12. To retrieve the results, go to 'Basic Search' and click on the number of results of the final query. Then, at the top of the records on the results page, click on the 'Export' drop-down menu and select 'CSV'. In the pop-up window, select 'Documents' and input 1 - to the results' number of the search, and click 'Export'. Use the same format as step 8, and do the same for the 'ris' format.

13. Repeat steps 10 to 12 for the systematic review 'SearchType' option queries.

14. Access the EBSCOhost electronic database: `https://search.ebscohost.com/`. Select 'EBSCOhost Web', select all, and continue. Then, select 'Search History' below the input boxes, and repeat steps 4-6 for article 'SearchType' option queries in EBSCOhost's first search box. Ensure no options are specified in the drop-down menu on the right of the input box.

15. There is no option to download the history in EBSCOhost as Excel, so click on 'Print Search History' at the top of the search history to open a window with a formatted search history. Right-click, print, and select 'Save as PDF'. Save in the same format as in step 7.

16. To retrieve the results, go to search history and click 'View Results' on the number of the results of the final query. On the results page, select 'Page Options' at the top right corner and choose 50 in the drop-down menu for 'Results per page'.

17. On the right of the 'Page Options', click 'Share' to open the drop-down menu, and select 'Add to Folder: Results (1-50)'. Afterwards, go to the bottom of the page and click 'Next' to page 2. Repeat this step for all pages.

18. Note that the final page results may not correspond to the total results in the search history. This is because EBSCOhost removes duplicates by default. Therefore, write the final results without duplicates into query S5 with filter in the '1SearchesHistory' sheet.

19. After selecting all the records, go to the top right corner of the page and click on the folder. In the folder, click 'Export' on the suitable menu options. In the 'Export Manager', select 'Download CSV' and click 'Save' to download the file to your computer. Use the same format as step 8, and do the same for the 'ris' format.

20. Repeat steps 14 to 19 for the systematic review ‘SearchType’ option queries.

21. Access the Web of Science electronic database: `https://www.webofscience.com/wos/woscc/advanced-search`, and at the top, in ‘Search in’, click on the drop-down menu and select ‘All databases’. Repeat steps 4-6 using articles ‘SearchType’ option queries for Web of Science, inserting the queries in the query preview, clicking on the search drop-down menu, and adding them to history.

22. To download the search history as Excel, click on the drop-down menu ‘Export’, select the Excel option, choose ‘All sets in this session’ and save the file on your computer. Save in the same format as in step 7.

23. To retrieve the results, go to history and click on the number of the final query results. On the results page, click the Export drop-down menu at the top of the records and select CSV. In the pop-up window, choose Records from, input the range from 1 to the total number of records, and click Export to save the file locally. Use the same naming format as in the previous step, and repeat the process for the ‘ris’ format. If the total number of records exceeds the limit (e.g., 1000), you must create multiple files until all records are saved.

24. Repeat steps 21 to 23 for the systematic review ‘SearchType’ option queries.

25. Using the exported results files, consolidate all records from the different files into the sheet titled ‘2SearchesResults’. For each record, fill in the following columns:

    - Number: Assign a unique, ordered number to each record.
    - SearchType: Indicate whether the record is an article or a systematic review.
    - SourceID: Provide a unique identifier for the database (e.g., ‘PMID’ for PubMed, ‘EID’ for Scopus, ‘Unique ID’ for Web of Science, or ‘an’ for EBSCOHost).
    - Title: Enter the title of the document.
    - Abstract: Include the abstract of the document.
    - Authors: List the authors of the document.
    - Journal: Specify the journal where the document was published.
    - Year: Indicate the publication year.
    - DOI: Provide the Digital Object Identifier (DOI) if available.
    - DocumentType: Specify the type of document (e.g., article, book, conference paper).

- Source: State the source of the document (e.g., PubMed, included studies).
- SpecificDatabase: Name the specific database or collection (e.g., collection name).
- SearchNumber: Indicate the number corresponding to the search iteration.
- Date: Record the date when the search was conducted.

26. After completing all searches, please send all the history search files and Excel files to the first author of the gold standard process for assessment, and proceed to initiate the next task upon approval.

5 Duplicates Task

1. Complete the required training, including familiarisation with documentation and assessment of three duplicates with this task.

2. An Excel file with '3Duplicates' sheet containing records from EBSCOhost, PubMed, Scopus, Web of Science, included studies, and systematic reviews references will be available using the Search Task files. A '1' in the database column indicates one record from the respective file provided. A '?' means that it needs to be checked if the record is also available in other database history search files. If there is more than one record in the same database, you put the number of times that record is repeated. If it is repeated two times, you write '2'.

3. To check the duplicates, use the function in Excel to highlight the duplicates. For this, select the Title column, then navigate to 'Conditional Formatting', 'Highlight Cells Rules', and 'Duplicate Values'.

4. Reorder the records based on the Title column. If duplicates are found (indicated by highlighted cells), fill in the number of times in all records in the respective database columns for the duplicates to indicate the presence of the record in multiple databases.

5. Double-check for any missing entries to ensure the database presence is correctly indicated. For example, records indicating the same study but with titles in another language, titles that differ by a period at the end or other slight modifications on the title.

6. Copy the records for a new temporary sheet to check for errors, if any, in the future. Then, select the column records, go to 'Data', select 'Remove Duplicates', and select the 'Title' column. This will delete all the duplicate records and keep their unique values.

7. To confirm that the numbers registered in each record for any database, you will select the cells with the numbers of duplicates for each database (i.e., D2:G{number of rows}), and at the bottom of Excel, you will confirm that sum number correspond to the number of rows of the rows before removing duplicates. You can use the temporary sheet to prove that value, and if the number does not correspond, correct the number of rows for each database. If it corresponds, delete the temporary sheet, and the task is completed.

   Note: If you did not create a temporary sheet, you can check using '2SearchResults' sheet.

8. After task completion, please forward all related files to the primary reviewer of the gold standard process for assessment, and proceed to initiate the next task upon approval.

6 Screening Task

1. Complete the required training, including familiarisation with documentation and screening three pre-screened records and reports, before proceeding with this task.

2. Copy the IDs and Title cells from '3Duplicates' to '4ScreeningTitles' sheet for records. Make sure that there is one record only for study. .

3. Keep the Supplementary Table S5 close for reference throughout the task. Please do not consult records information to not compromise blinding. If you have any questions, contact the first and second reviewers of the gold standard process.

4. Using the Title, check Criterion 1 and assess whether it meets the criterion (yes or no) based on the document from step 3. Be cautious that the criteria in the header are inclusion criteria to avoid confusion with double negatives. However, it should be seen as an exclusion criterion, i.e., if one criterion is not met, it is excluded. Yet, you have to access all criteria for other purposes.

5. If Criterion 1 is assessed as 'no', provide a justification or an in-text sentence that explains the 'no' answer. If quoting directly from the article, use quotation marks to distinguish between the text and your own words. Use 'NA' if the criterion or justification is not applicable. As default, if a record is not an observational study or does not have a sample, all the remaining criteria are assessed as 'NA'.

6. The 'Result' and 'First Reason Exclusion' cells of '4ScreeningTitles' contain formulas to facilitate your work. Double-check these and, at the end, copy and paste them as text to remove the formulas.

7. Repeat steps 4-6 for the remaining criteria in ascending order. If the 'SearchType' of the record is a systematic review, you only check for male (criterion 4) and soccer (criterion 1) inclusion; the other fills with 'NA'.

8. After assessing all criteria, send the list of records (IDs and Title columns only) that are advancing to the abstracts screening stage to the first author of the gold standard process so that the abstracts can be made available to you without compromising blinding.

9. After receiving the records with abstracts, copy the records for '5ScreeningAbstracts' and repeat steps 4-7 for the screening phase using abstracts.

10. After assessing all criteria for abstracts, send the list of records (IDs and Title columns only) advancing from abstract screening to full-text screening to the first author of the gold standard process so that the full-texts can be made available to you without compromising blinding.

11. Once you receive the records and the full-text files blinded from the first author, copy the records to the '6ScreeningFullText' sheet and assess the reports using the full-texts, i.e., repeat steps 4-7.

12. After assessing all criteria for full-texts, send the list of records (IDs and Title columns only) of the included studies and systematic reviews to the first author of the gold standard process so that the records of included studies and systematic review can be made available to you without compromising blinding.

13. Once you receive the records from the first author, copy them to the '3Duplicates' sheet, repeat duplicates and screening task for the new records (i.e., copying and sending the records between sheets and to the first author) until there are no more included studies.

14. After task completion, please forward all related files to the primary reviewer of the gold standard process for assessment, and proceed to initiate the next task upon approval.

7 Data Collection Task

1. Complete the required training, including familiarisation with documentation and extract data of three pre-extracted reports, before proceeding with this task.

2. Copy the ID cells of the included studies from the '6ScreeningFullTexts' sheet to the '7DataMetadata', '8DataPopulation', '9DataIntervention', '10DataOutcome', '11DataComparisons', '12DataStudyDesign' sheets.

3. Keep Supplementary Table S8 close for reference throughout the task. Please, do not consult reports' information to not compromise blinding. If you have any questions, contact the first and second reviewers.

4. For each included study, extract the necessary information from the full-text. Fill in each field based on the column headers in the '7DataMetadata' sheet.

5. Insert categorical data using the drop-down menu by selecting the appropriate cell. For list data structures, insert values separated by commas without spaces or/and respecting the format in Supplementary Table S8, e.g., Apples,Oranges,Grapes.

6. Create multiple rows for the same study as needed for any header that contains more than a single entity, except for lists. Write ´NA' if there is no comparator, subgroup or other data.

7. Repeat steps 4-6 for '8DataPopulation', '9DataIntervention', '10DataOutcome', '11DataComparisons', '12DataStudyDesign' sheets, following the specified order.

8. After task completion, please forward all related files to the primary reviewer of the gold standard process for assessment and proceed to initiate the next task upon approval.

8 Data Assessment Task

1. Download the tool and documentation for ROBINS-I from `https://www.riskofbias.info/welcome/home/current-version-of-robins-i/robins-i-tool-2016` or the GRADE handbook `https://gdt.gradepro.org/app/handbook/handbook.html` depending on the tool for use in the task. This documentation will also be available in the repository. Keep this documentation close for reference throughout the task.

2. Complete the required training, including familiarisation with documentation and assess three pre-extracted reports, before proceeding with this task.

3. For ROBINS-I, copy the ID columns' cells of all records from the '7DataMetadata' sheet to the '13Assessment' sheet.

4. Using the full-texts from the Screening task, assess each study for all headers starting with 'RoBi'. Use the drop-down menu by selecting the appropriate cell. Each number corresponds to a question in the tool from step 1.

5. After task completion, please forward all related files to the primary reviewer of the gold standard process for assessment and proceed to initiate the next task upon approval or confirmation that the work is finished.

6. For GRADE, use the following app: `https://gdt.gradepro.org/app/#projects`. Create an account and log in.

7. In the app, create a project with a name of your choice. Then, under the 'Comparisons' section on the left, you will create diagnostic questions using Interventions 1 and 2 in comparisons from the '11DataComparisons' sheet that includes more than 3 studies for the same outcome. Follow the handbook and use the interactive user interface to create outcomes and filling the appropriate data by clicking on each cell. You can refer to the video tutorials if you still have any doubts: `https://www.youtube.com/watch?v=iSmbOiIoF_g&list=PLxFw8aTtvq-c90anp9-RN0jD7Gnq_xPxj`.

8. For each question created, export the table using the button at the top right corner, send them all to the first author of the gold standard assessment process, and initiate the next task upon approval or confirmation that the work is finished.

9 Cleaning and Checking

At the end, make sure to clean the following:

1. Formulas: Convert formulas to text by copying and pasting them as text. Ensure all formulas are properly converted.
2. Conditional Formatting: Make sure all conditional formatting rules are deleted. Go to 'Conditional Formatting', 'Clear rules' and 'Clear Rules from Entire Sheet'.
3. '?' Characters: Remove any '?' characters used as placeholders for reviewers to fill in data. Delete rows containing '?' characters, which indicates incomplete data that needs assessment and completion. If you use the function 'Replace', use '?' to find instead of '?', as the latter will replace any text.
