# Supplementary material for: Ranking sports science and medicine interventions impacting team performance: a protocol for a systematic review and meta-analysis of observational studies in elite football
Source: BMJ Open Sport Exerc Med. 2024 Sep 13;10(3):e002196. doi: 10.1136/bmjsem-2024-002196 (PMC11404162; doi:10.1136/bmjsem-2024-002196)
Supplement: online supplemental file 5 [file bmjsem-10-3-s005.pdf]

Supplementary Table S5. Inclusion and exclusion criteria.

Inclusion criteria[a]	Scale[b]	Description
1-The research application area is football or soccer. (P)	Yes	A record or report containing soccer or football terms unrelated to rugby or American football – e.g., European or Association Football.
	No	A record or report that does not contain the term soccer. It can contain football terms related to rugby or American football, including Australian Football or National Football League.
2-The study design is an observational type. (S)	Yes	A record or report with research designs without randomisation, where data can be previously collected. For more details, see Rosenbaum [1]
	No	A record or report with experimental research designs, i.e., with randomisation – e.g., randomised controlled trials.
3-The sample of the study is adults. (P)	Yes	The study participants are integrated into a team's categorisation of 19 years or older - e.g., U21.
	No	The study participants are not integrated into a team's categorisation 19 years or older - e.g., U16.
4-The sample's genre is described as male. (P)	Yes	The study participants' genres are characterised as men.
	No	The study participants' genres are not described as men – e.g., female or women.
5-The sample is elite or world-class. (P)	Yes	The study's participants correspond to Tier 4-5 of the Participant Classification Framework [2] — e.g., FIFA international competitions.
	No	The study's participants correspond to Tier 1-3 of the Participant Classification Framework [2] — e.g., domestic second divisions.
6- The unit of analysis is teams or team observations (P)	Yes	A record or report that conducts statistical analysis using variables measured from a group of players playing together in organised and regulated game matches.
	No	A record or report that conducts statistical analysis using variables measured from single-unit groups – e.g., players or referees.
7- The data is collected from official competitive matches (P)	Yes	A record or report that registers measurements from a structured game from a tournament or league regulated by FIFA or its members – e.g., Primeira Liga from the Portuguese Football Federation.
	No	A record or report does not register measures from the games defined in this criterion's 'Yes' scale – e.g., friendly matches and matches on training.

Note. Supplementary Table S5 continued on the next page.

Supplementary Table S5 (Continued)

**Inclusion criteria**[a]	**Scale**[b]	**Description**
8-The research question includes team match interventions as an independent or predictor variable. (I, C)	Yes	A record or report conducts statistical analysis using team match intervention input variables, referring to the deliberate strategies, prescriptions, or actions implemented on teams or players by agents that do not participate directly in the team playing, e.g., coaches, to influence or modify team match performance and, subsequently, their success or effectiveness.
	No	A record or report does not conduct statistical analysis using team match intervention input variables defined in this criterion's 'Yes' scale – e.g., contextual variables, constraints, or technologies that cannot be controlled or are standard to matches, such as match location, pitch size, or video assistant referee (VAR), are marked as 'No'.
9-The research question includes a match performance or a competition success measure as a dependent or outcome variable. (O)	Yes	A record or report conducts statistical analysis using output variables such as goals scored, goals conceded, goals difference, number of winnings, number of losses, the difference between winning and losses, match outcome, team final ranking, number of points and difference of number of points, or other related variables. It also includes variables that measure team match effects such as time motion, match or injury statistics.
	No	A record or report does not conduct statistical analysis using the output variables defined in this criterion's 'Yes' scale.

Note. The numbering before each criterion corresponds to the order in which the criteria are checked.

[a] Exclusion criteria are the negative counterparts of the inclusion criteria;

[b] The scale codification is No (=0), Yes (=1), and the standard answer for all of "Not applicable/No information"(=NA), which is used when the provided information does not fit into any of the scale categories, i.e., 'Yes' or 'No';

(P): Population; (I, C): Intervention, Comparison; (O): Outcome; (S): Study design.

[1] Rosenbaum PR. Observational study: definition and examples. Wiley StatsRef: Statistics Reference Online. 2014. https://doi.org/10.1002/9781118445112.stat06714

[2] McKay AKA, Stellingwerff T, Smith ES, Martin DT, Mujika I, Goosey-Tolfrey VL, et al. Defining training and performance caliber: a participant classification framework. Int J Sports Physiol Perform. 2022;17(2):317-31. https://doi.org/10.1123/ijspp.2021-0451
