# Supplementary material for: Ranking sports science and medicine interventions impacting team performance: a protocol for a systematic review and meta-analysis of observational studies in elite football
Source: BMJ Open Sport Exerc Med. 2024 Sep 13;10(3):e002196. doi: 10.1136/bmjsem-2024-002196 (PMC11404162; doi:10.1136/bmjsem-2024-002196)
Supplement: online supplemental file 6 [file bmjsem-10-3-s006.pdf]

Supplementary File S6
Preliminary queries and search results

Number	Database	Collections	All/TAK	Duplicates	Query_Pubmed_All	Results	Date
#1(Population)	Pubmed	Pubmed	All	No	(soccer OR football)	29,104	30-Mar-2024
#2(Intervention)	Pubmed	Pubmed	All	No	(tactical OR strategy OR "performance indicators" OR "key performance indicators" OR metric)	826,905	30-Mar-2024
#3(Comparison)	Pubmed	Pubmed	All	No	(physical OR technical OR injury OR conditioning OR psychological)	3,322,464	30-Mar-2024
#4(Outcome)	Pubmed	Pubmed	All	No	(winning OR "match outcome" OR win)	18,223	30-Mar-2024
#5(Study Design)	Pubmed	Pubmed	All	No	(“notational analysis” OR “performance analysis” OR “match analysis” OR "observational")	349,617	30-Mar-2024
#6	Pubmed	Pubmed	All	No	#1 AND #2	1,500	30-Mar-2024
#7	Pubmed	Pubmed	All	No	#1 AND #2 AND #3	1,072	30-Mar-2024
#8	Pubmed	Pubmed	All	No	#1 AND #2 AND #3 AND #4	60	30-Mar-2024
#9	Pubmed	Pubmed	All	No	#1 AND #2 AND #3 AND #4 AND #5	26	30-Mar-2024
#10	Pubmed	Pubmed	All	No	(tactical* OR strategy* OR "performance indicator" OR "key performance indicators" OR metric* OR variables* OR statistic* OR indicator*)	4,367,396	30-Mar-2024
#11	Pubmed	Pubmed	All	No	(physical* OR technical* OR injury* OR conditioning* OR psychological* OR loading* OR time-motion*)	356,502	30-Mar-2024
#12	Pubmed	Pubmed	All	No	#1 AND #10 AND #11 AND #4 AND #5	48	30-Mar-2024
#17	Pubmed	Pubmed	All	No	((soccer OR football) AND (elite* OR top OR high*) AND (adult OR male) NOT female NOT Youth NOT Young NOT "Australian Football" NOT "National Football League" NOT "Gaelic Football")	2,347	30-Mar-2024
#18	Pubmed	Pubmed	All	No	(tactic* OR strateg* OR collective OR attack* OR defend* OR defens* OR offens* OR "patterns of play" OR coordinat* OR style OR interaction* OR intra* OR inter* OR coupling* OR organize?action OR tactical-technical)	16,292,391	30-Mar-2024
#19	Pubmed	Pubmed	All	No	(techni* OR skill* OR technical-tactical OR motor* OR goal* OR pass* OR assist* OR tackl* OR behavio?ral OR head* OR shot* OR ball* OR entr* OR task* OR possession OR zone* OR position* OR space OR "time?space" OR spational?temporal OR physic* OR load* OR conditioning OR injur* OR physiolog* OR strength OR running OR athletic* OR sprint* OR acceleration* OR jump* OR speed* OR work?rate OR wellness OR biomechanic* OR neuromuscular OR fitness OR acute OR chronic OR intensit* OR train* OR movement* OR "activity profile*" OR distance* OR anthropometric* OR recover* OR agility OR psycholog* OR percept* OR action* OR decision*making OR attention OR mental OR emotion* OR role* OR cogniti* OR ecologic* OR visual OR verbal OR emergen* OR complex OR constraint* OR affordances OR enviroment* OR spectator* OR crowd OR situation* OR context*)	24,287,652	30-Mar-2024
#20	Pubmed	Pubmed	All	No	(outcome OR winn* OR win OR odds OR "odds?ratio" OR expected OR probability)	5,705,831	30-Mar-2024
#21	Pubmed	Pubmed	All	No	(“notational analysis” OR "composite variable" OR “performance analysis” OR “match analysis” OR "game analysis" OR "match statistic*" OR "observational" OR time?motion OR network* OR "performance indicator*" OR "key performance indicator*" OR metric* OR index* OR indicator* OR pattern* OR "sequential analysis" OR measure* OR factor* OR latent* OR ratio* OR match?play OR "match activiti*" OR "multi?dimension*" OR profile OR "multi?factorial" OR coefficient* OR score* OR cluster*)	14,667,998	30-Mar-2024
#22	Pubmed	Pubmed	All	No	#17 AND #18 AND #19 AND #20 AND #21 = ((soccer OR football) AND (elite* OR top OR high*) AND (adult OR male) NOT female NOT Youth NOT Young NOT "Australian Football" NOT "National Football League" NOT "Gaelic Football"AND(tactic* OR strateg* OR collective OR attack* OR defend* OR defens* OR offens* OR "patterns of play" OR coordinat* OR style OR interaction* OR intra* OR inter* OR coupling* OR organize?action OR tactical-technical)AND(techni* OR skill* OR technical-tactical OR motor* OR goal* OR pass* OR assist* OR tackl* OR behavio?ral OR head* OR shot* OR ball* OR entr* OR task* OR possession OR zone* OR position* OR space OR "time?space" OR spational?temporal OR physic* OR load* OR conditioning OR injur* OR physiolog* OR strength OR running OR athletic* OR sprint* OR acceleration* OR jump* OR speed* OR work?rate OR wellness OR biomechanic* OR neuromuscular OR fitness OR acute OR chronic OR intensit* OR train* OR movement* OR "activity profile*" OR distance* OR anthropometric* OR recover* OR agility OR psycholog* OR percept* OR action* OR decision*making OR attention OR mental OR emotion* OR role* OR cogniti* OR ecologic* OR visual OR verbal OR emergen* OR complex OR constraint* OR affordances OR enviroment* OR spectator* OR crowd OR situation* OR context*) AND(outcome OR winn* OR win OR odds OR "odds?ratio" OR expected OR probability)AND(“notational analysis” OR "composite variable" OR “performance analysis” OR “match analysis” OR "game analysis" OR "match statistic*" OR "observational" OR time?motion OR network* OR "performance indicator*" OR "key performance indicator*" OR metric* OR index* OR indicator* OR pattern* OR "sequential analysis" OR measure* OR factor* OR latent* OR ratio* OR match?play OR "match activiti*" OR "multi?dimension*" OR profile OR "multi?factorial" OR coefficient* OR score* OR cluster*))	399	30-Mar-2024
#23(Population)	Pubmed	Pubmed	Title-Abstract-Keywords	No	(soccer[Title/Abstract] OR football[Title/Abstract])	22,849	30-Mar-2024
#24(Intervention)	Pubmed	Pubmed	Title-Abstract-Keywords	No	(tactical[Title/Abstract] OR strategy[Title/Abstract] OR "performance indicators"[Title/Abstract] OR "key performance indicators"[Title/Abstract] OR metric[Title/Abstract])	816,253	30-Mar-2024
#25(Comparison)	Pubmed	Pubmed	Title-Abstract-Keywords	No	(physical[Title/Abstract] OR technical[Title/Abstract] OR injury[Title/Abstract] OR conditioning[Title/Abstract] OR psychological[Title/Abstract])	2,255,485	30-Mar-2024
#26(Outcome)	Pubmed	Pubmed	Title-Abstract-Keywords	No	(winning[Title/Abstract] OR "match outcome"[Title/Abstract] OR win[Title/Abstract])	15,052	30-Mar-2024
#27(Study Design)	Pubmed	Pubmed	Title-Abstract-Keywords	No	(“notational analysis”[Title/Abstract] OR “performance analysis”[Title/Abstract] OR “match analysis”[Title/Abstract] OR "observational"[Title/Abstract])	292,085	30-Mar-2024
#28	Pubmed	Pubmed	Title-Abstract-Keywords	No	#23 AND #24	1,273	30-Mar-2024
#29	Pubmed	Pubmed	Title-Abstract-Keywords	No	#23 AND #24 AND #25	717	30-Mar-2024
#30	Pubmed	Pubmed	Title-Abstract-Keywords	No	#23 AND #24 AND #25 AND #26	36	30-Mar-2024
#31	Pubmed	Pubmed	Title-Abstract-Keywords	No	#23 AND #24 AND #25 AND #26 AND #27	12	30-Mar-2024
#32	Pubmed	Pubmed	Title-Abstract-Keywords	No	(tactical*[Title/Abstract] OR strategy*[Title/Abstract] OR "performance indicator"[Title/Abstract] OR "key performance indicators"[Title/Abstract] OR metric*[Title/Abstract] OR variables*[Title/Abstract] OR statistic*[Title/Abstract] OR indicator* [Title/Abstract])	3,140,084	30-Mar-2024
#33	Pubmed	Pubmed	Title-Abstract-Keywords	No	(physical*[Title/Abstract] OR technical*[Title/Abstract] OR injury*[Title/Abstract] OR conditioning*[Title/Abstract] OR psychological*[Title/Abstract] OR loading*[Title/Abstract] OR time-motion*[Title/Abstract])	2,510,700	30-Mar-2024
#34	Pubmed	Pubmed	Title-Abstract-Keywords	No	#23 AND #32 AND #33 AND #26 AND #27	26	30-Mar-2024
#39	Pubmed	Pubmed	Title-Abstract-Keywords	No	((soccer[Title/Abstract] OR football[Title/Abstract]) AND (elite*[Title/Abstract] OR top[Title/Abstract] OR high*[Title/Abstract]) AND (adult[Title/Abstract] OR male[Title/Abstract]) NOT female[Title/Abstract] NOT Youth[Title/Abstract] NOT Young[Title/Abstract] NOT "Australian Football"[Title/Abstract] NOT "National Football League"[Title/Abstract] NOT "Gaelic Football"[Title/Abstract])	1,953	30-Mar-2024

Number	Database	Collections	All/TAK	Duplicates	Query_Pubmed_All	Results	Date
#40	Pubmed	Pubmed	Title-Abstract-Keywords	No	(tactic*[Title/Abstract] OR strateg*[Title/Abstract] OR collective[Title/Abstract] OR attack*[Title/Abstract] OR defend*[Title/Abstract] OR defens*[Title/Abstract] OR offens*[Title/Abstract] OR "patterns of play"[Title/Abstract] OR coordinat*[Title/Abstract] OR style[Title/Abstract] OR interaction*[Title/Abstract] OR intra*[Title/Abstract] OR inter*[Title/Abstract] OR coupling*[Title/Abstract] OR organize?action[Title/Abstract] OR tactical-technical[Title/Abstract])	12,185,570	30-Mar-2024
#41	Pubmed	Pubmed	Title-Abstract-Keywords	No	(techni*[Title/Abstract] OR skill*[Title/Abstract] OR technical-tactical[Title/Abstract] OR motor*[Title/Abstract] OR goal*[Title/Abstract] OR pass*[Title/Abstract] OR assist*[Title/Abstract] OR tackl*[Title/Abstract] OR behavio?ral[Title/Abstract] OR head*[Title/Abstract] OR shot*[Title/Abstract] OR ball*[Title/Abstract] OR entr*[Title/Abstract] OR task*[Title/Abstract] OR possession[Title/Abstract] OR zone*[Title/Abstract] OR position*[Title/Abstract] OR space[Title/Abstract] OR "time?space"[Title/Abstract] OR spational?temporal[Title/Abstract] OR physic*[Title/Abstract] OR load*[Title/Abstract] OR conditioning[Title/Abstract] OR injur*[Title/Abstract] OR physiolog*[Title/Abstract] OR strength[Title/Abstract] OR running[Title/Abstract] OR athletic*[Title/Abstract] OR sprint*[Title/Abstract] OR acceleration[Title/Abstract] OR jump*[Title/Abstract] OR speed[Title/Abstract] OR work?rate[Title/Abstract] OR wellness[Title/Abstract] OR biomechanic*[Title/Abstract] OR neuromuscular[Title/Abstract] OR fitness[Title/Abstract] OR acute[Title/Abstract] OR chronic[Title/Abstract] OR intensit*[Title/Abstract] OR train*[Title/Abstract] OR movement*[Title/Abstract] OR "activity profile*"[Title/Abstract] OR distance*[Title/Abstract] OR anthropometric*[Title/Abstract] OR recover*[Title/Abstract] OR agility[Title/Abstract] OR psycholog*[Title/Abstract] OR percept*[Title/Abstract] OR action*[Title/Abstract] OR decision*making[Title/Abstract] OR attention[Title/Abstract] OR mental[Title/Abstract] OR emotion*[Title/Abstract] OR role*[Title/Abstract] OR cogniti*[Title/Abstract] OR ecologic*[Title/Abstract] OR visual[Title/Abstract] OR verbal[Title/Abstract] OR emergen*[Title/Abstract] OR complex[Title/Abstract] OR constraint*[Title/Abstract] OR affordances[Title/Abstract] OR enviroment*[Title/Abstract] OR spectator*[Title/Abstract] OR crowd[Title/Abstract] OR situation*[Title/Abstract] OR context*[Title/Abstract])	17,554,581	30-Mar-2024
#42	Pubmed	Pubmed	Title-Abstract-Keywords	No	(outcome[Title/Abstract] OR winn*[Title/Abstract] OR win[Title/Abstract] OR odds[Title/Abstract] OR "odds?ratio"[Title/Abstract] OR expected[Title/Abstract] OR probability[Title/Abstract])	2,378,431	30-Mar-2024
#43	Pubmed	Pubmed	Title-Abstract-Keywords	No	(“notational analysis”[Title/Abstract] OR "composite variable"[Title/Abstract] OR “performance analysis”[Title/Abstract] OR “match analysis”[Title/Abstract] OR "game analysis"[Title/Abstract] OR "match statistic*"[Title/Abstract] OR "observational"[Title/Abstract] OR time*motion[Title/Abstract] OR network[Title/Abstract] OR "performance indicator*"[Title/Abstract] OR "key performance indicator*"[Title/Abstract] OR metric*[Title/Abstract] OR index*[Title/Abstract] OR indicator*[Title/Abstract] OR pattern*[Title/Abstract] OR "sequential analysis"[Title/Abstract] OR measure*[Title/Abstract] OR factor*[Title/Abstract] OR latent*[Title/Abstract] OR ratio*[Title/Abstract] OR match?play[Title/Abstract] OR "match activiti*"[Title/Abstract] OR "multi?dimension*"[Title/Abstract] OR profile[Title/Abstract] OR "multi?factorial"[Title/Abstract] OR coefficient*[Title/Abstract] OR score*[Title/Abstract] OR cluster*[Title/Abstract])	12,248,630	30-Mar-2024
#44	Pubmed	Pubmed	Title-Abstract-Keywords	No	#39 AND #40 AND #41 AND #42 AND #43	182	30-Mar-2024
#1(Population)	Scopus	Scopus	All	No	(soccer OR football)	229,210	30-Mar-2024
#2(Intervention)	Scopus	Scopus	All	No	(tactical OR strategy OR "performance indicators" OR "key performance indicators" OR metric)	13,267,910	30-Mar-2024
#3(Comparison)	Scopus	Scopus	All	No	(physical OR technical OR injury OR conditioning OR psychological)	28,618,918	30-Mar-2024
#4(Outcome)	Scopus	Scopus	All	No	(winning OR "match outcome" OR win)	13,394,985	30-Mar-2024
#5(Study Design)	Scopus	Scopus	All	No	(“notational analysis” OR “performance analysis” OR “match analysis” OR "observational")	2,354,593	30-Mar-2024
#6	Scopus	Scopus	All	No	#1 AND #2	82,213	30-Mar-2024
#7	Scopus	Scopus	All	No	#1 AND #2 AND #3	63,715	30-Mar-2024
#8	Scopus	Scopus	All	No	#1 AND #2 AND #3 AND #4	25,306	30-Mar-2024
#9	Scopus	Scopus	All	No	#1 AND #2 AND #3 AND #4 AND #5	5,535	30-Mar-2024
#10	Scopus	Scopus	All	No	(tactical* OR strategy* OR "performance indicator" OR "key performance indicators" OR metric* OR variables* OR statistic* OR indicator*)	26,410,731	30-Mar-2024
#11	Scopus	Scopus	All	No	(physical* OR technical* OR injury* OR conditioning* OR psychological* OR loading* OR time-motion*)	29,941,885	30-Mar-2024
#12	Scopus	Scopus	All	No	#1 AND #10 AND #11 AND #4 AND #5	2,932	30-Mar-2024
#17	Scopus	Scopus	All	No	(soccer OR football) AND (elite* OR top OR high*) AND (adult OR male) AND NOT female AND NOT Youth AND NOT Young AND NOT "Australian Football" AND NOT "National Football League" AND NOT "Gaelic Football"	10,122	30-Mar-2024
#18	Scopus	Scopus	All	No	(tactic* OR strateg* OR collective OR attack* OR defend* OR defens* OR offens* OR "patterns of play" OR coordinat* OR style OR interaction* OR intra* OR inter* OR coupling* OR organize?action OR tactical-technical)	65,314,407	30-Mar-2024
#19	Scopus	Scopus	All	No	(techni* OR skill* OR technical-tactical OR motor* OR goal* OR pass* OR assist* OR tackl* OR behavio?ral OR head* OR shot* OR ball* OR entr* OR task* OR possession OR zone* OR position* OR space OR "time?space" OR spational?temporal OR physic* OR load* OR conditioning OR injur* OR physiolog* OR strength OR running OR athletic* OR sprint* OR acceleration* OR jump* OR speed* OR work?rate OR wellness OR biomechanic* OR neuromuscular OR fitness OR acute OR chronic OR intensit* OR train* OR movement* OR "activity profile*" OR distance* OR anthropometric* OR recover* OR agility OR psycholog* OR percept* OR action* OR decision*making OR attention OR mental OR emotion* OR role* OR cogniti* OR ecologic* OR visual OR verbal OR emergen* OR complex OR constraint* OR affordances OR enviroment* OR spectator* OR crowd OR situation* OR context*)	74,257,881	30-Mar-2024
#20	Scopus	Scopus	All	No	(outcome OR winn* OR win OR odds OR "odds?ratio" OR expected OR probability)	14,697,058	30-Mar-2024
#21	Scopus	Scopus	All	No	(“notational analysis” OR "composite variable" OR “performance analysis” OR “match analysis” OR "game analysis" OR "match statistic*" OR "observational" OR time?motion OR network* OR "performance indicator*" OR "key performance indicator*" OR metric* OR index* OR indicator* OR pattern* OR "sequential analysis" OR measure* OR factor* OR latent* OR ratio* OR match?play OR "match activiti*" OR "multi?dimension*" OR profile OR "multi?factorial" OR coefficient* OR score* OR cluster*)	58,653,925	30-Mar-2024

Number	Database	Collections	All/TAK	Duplicates	Query_Pubmed_All	Results	Date
#22	Scopus	Scopus	All	No	#17 AND #18 AND #19 AND #20 AND #21 = ((soccer OR football) AND (elite* OR top OR high*) AND (adult OR male) NOT female NOT Youth NOT Young NOT "Australian Football" NOT "National Football League" NOT "Gaelic Football"AND(tactic* OR strateg* OR collective OR attack* OR defend* OR defens* OR offens* OR "patterns of play" OR coordinat* OR style OR interaction* OR intra* OR inter* OR coupling* OR organize?action OR tactical-technical)AND(techni* OR skill* OR technical-tactical OR motor* OR goal* OR pass* OR assist* OR tackl* OR behavio?ral OR head* OR shot* OR ball* OR entr* OR task* OR possession OR zone* OR position* OR space OR "time?space" OR spational?temporal OR physic* OR load* OR conditioning OR injur* OR physiolog* OR strength OR running OR athletic* OR sprint* OR acceleration* OR jump* OR speed* OR work?rate OR wellness OR biomechanic* OR neuromuscular OR fitness OR acute OR chronic OR intensit* OR train* OR movement* OR "activity profile*" OR distance* OR anthropometric* OR recover* OR agility OR psycholog* OR percept* OR action* OR decision*making OR attention OR mental OR emotion* OR role* OR cogniti* OR ecologic* OR visual OR verbal OR emergen* OR complex OR constraint* OR affordances OR enviroment* OR spectator* OR crowd OR situation* OR context*) AND(outcome OR winn* OR win OR odds OR "odds?ratio" OR expected OR probability)AND("notational analysis" OR "composite variable" OR "performance analysis" OR "match analysis" OR "game analysis" OR "match statistic*" OR "observational" OR time?motion OR network* OR "performance indicator*" OR "key performance indicator*" OR metric* OR index* OR indicator* OR pattern* OR "sequential analysis" OR measure* OR factor* OR latent* OR ratio* OR match?play OR "match activiti*" OR "multi?dimension*" OR profile OR "multi?factorial" OR coefficient* OR score* OR cluster*))	10,152	30-Mar-2024
#23(Population)	Scopus	Scopus	Title-Abstract-Keywords	No	TITLE-ABS-KEY((soccer) OR (football))	66,741	30-Mar-2024
#24(Intervention)	Scopus	Scopus	Title-Abstract-Keywords	No	TITLE-ABS-KEY((tactical) OR (strategy) OR ("performance indicators") OR ("key performance indicators") OR (metric))	4,592,348	30-Mar-2024
#25(Comparison)	Scopus	Scopus	Title-Abstract-Keywords	No	TITLE-ABS-KEY((physical) OR (technical) OR (injury) OR (conditioning) OR (psychological))	8,224,461	30-Mar-2024
#26(Outcome)	Scopus	Scopus	Title-Abstract-Keywords	No	TITLE-ABS-KEY((winning) OR ("match outcome") OR (win))	93,650	30-Mar-2024
#27(Study Design)	Scopus	Scopus	Title-Abstract-Keywords	No	TITLE-ABS-KEY((“notational analysis”) OR (“performance analysis”) OR (“match analysis”) OR ("observational"))	660,595	30-Mar-2024
#28	Scopus	Scopus	Title-Abstract-Keywords	No	#23 AND #24	8,071	30-Mar-2024
#29	Scopus	Scopus	Title-Abstract-Keywords	No	#23 AND #24 AND #25	3,465	30-Mar-2024
#30	Scopus	Scopus	Title-Abstract-Keywords	No	#23 AND #24 AND #25 AND #26	139	30-Mar-2024
#31	Scopus	Scopus	Title-Abstract-Keywords	No	#23 AND #24 AND #25 AND #26 AND #27	35	30-Mar-2024
#32	Scopus	Scopus	Title-Abstract-Keywords	No	TITLE-ABS-KEY((tactical*) OR (strategy*) OR ("performance indicator") OR ("key performance indicators") OR (metric*) OR (variables*) OR (statistic*) OR (indicator*))	10,973,043	30-Mar-2024
#33	Scopus	Scopus	Title-Abstract-Keywords	No	TITLE-ABS-KEY((physical*) OR (technical*) OR (injury*) OR (conditioning*) OR (psychological*) OR (loading*) OR (time-motion*))	9,275,336	30-Mar-2024
#34	Scopus	Scopus	Title-Abstract-Keywords	No	#23 AND #32 AND #33 AND #26 AND #27	62	30-Mar-2024
#39	Scopus	Scopus	Title-Abstract-Keywords	No	TITLE-ABS-KEY((soccer OR football) AND (elite* OR top OR high*) AND (adult OR male) AND NOT female AND NOT Youth AND NOT Young AND NOT "Australian Football" AND NOT "National Football League" AND NOT "Gaelic Football")	4,252	30-Mar-2024
#40	Scopus	Scopus	Title-Abstract-Keywords	No	TITLE-ABS-KEY((tactic* OR strateg* OR collective OR attack* OR defend* OR defens* OR offens* OR "patterns of play" OR coordinat* OR style OR interaction* OR intra* OR inter* OR coupling* OR organize?action OR tactical-technical))	33,555,182	30-Mar-2024
#41	Scopus	Scopus	Title-Abstract-Keywords	No	TITLE-ABS-KEY(techni* OR skill* OR technical-tactical OR motor* OR goal* OR pass* OR assist* OR tackl* OR behavio?ral OR head* OR shot* OR ball* OR entr* OR task* OR possession OR zone* OR position* OR space OR "time?space" OR spational?temporal OR physic* OR load* OR conditioning OR injur* OR physiolog* OR strength OR running OR athletic* OR sprint* OR acceleration* OR jump* OR speed* OR work?rate OR wellness OR biomechanic* OR neuromuscular OR fitness OR acute OR chronic OR intensit* OR train* OR movement* OR "activity profile*" OR distance* OR anthropometric* OR recover* OR agility OR psycholog* OR percept* OR action* OR decision*making OR attention OR mental OR emotion* OR role* OR cogniti* OR ecologic* OR visual OR verbal OR emergen* OR complex OR constraint* OR affordances OR enviroment* OR spectator* OR crowd OR situation* OR context*)	52,937,050	30-Mar-2024
#42	Scopus	Scopus	Title-Abstract-Keywords	No	TITLE-ABS-KEY((outcome OR winn* OR win OR odds OR "odds?ratio" OR expected OR probability))	7,756,204	30-Mar-2024
#43	Scopus	Scopus	Title-Abstract-Keywords	No	TITLE-ABS-KEY("notational analysis" OR "composite variable" OR "performance analysis" OR "match analysis" OR "game analysis" OR "match statistic*" OR "observational" OR time?motion OR network* OR "performance indicator*" OR "key performance indicator*" OR metric* OR index* OR indicator* OR pattern* OR "sequential analysis" OR measure* OR factor* OR latent* OR ratio* OR match?play OR "match activiti*" OR "multi?dimension*" OR profile OR "multi?factorial" OR coefficient* OR score* OR cluster*)	36,159,157	30-Mar-2024
#44	Scopus	Scopus	Title-Abstract-Keywords	No	#39 AND #40 AND #41 AND #42 AND #43	387	30-Mar-2024
#1(Population)	WoS	Web of Science Core Collection;Current Contents Connect;Derwent Innovations Index;Grants Index;KCI-Korean Journal Database;MEDLINE;ProQuest;SciELO	All	No	ALL=(soccer OR football)	62,068	9-Apr-2024
#2(Intervention)	WoS	Web of Science Core Collection;Current Contents Connect;Derwent Innovations Index;Grants Index;KCI-Korean Journal Database;MEDLINE;ProQuest;SciELO	All	No	ALL=("tactical" OR "strategy" OR "performance indicators" OR "key performance indicators" OR "metric")	2,108,361	9-Apr-2024
#3(Comparison)	WoS	Web of Science Core Collection;Current Contents Connect;Derwent Innovations Index;Grants Index;KCI-Korean Journal Database;MEDLINE;ProQuest;SciELO	All	No	ALL=("physical" OR "technical" OR "injury" OR "conditioning" OR "psychological")	7,039,164	9-Apr-2024
#4(Outcome)	WoS	Web of Science Core Collection;Current Contents Connect;Derwent Innovations Index;Grants Index;KCI-Korean Journal Database;MEDLINE;ProQuest;SciELO	All	No	ALL=("winning" OR "match outcome" OR win*)	2,367,365	9-Apr-2024

Number	Database	Collections	All/TAK	Duplicates	Query_Pubmed_All	Results	Date
#5(Study Design)	WoS	Web of Science Core Collection;Current Contents Connect;Derwent Innovations Index;Grants Index;KCI-Korean Journal Database;MEDLINE;ProQuest;SciELO	All	No	ALL=(“notational analysis” OR “performance analysis” OR “match analysis” OR "observational")	477,100	9-Apr-2024
#6	WoS	Web of Science Core Collection;Current Contents Connect;Derwent Innovations Index;Grants Index;KCI-Korean Journal Database;MEDLINE;ProQuest;SciELO	All	No	#1 AND #2	4,303	9-Apr-2024
#7	WoS	Web of Science Core Collection;Current Contents Connect;Derwent Innovations Index;Grants Index;KCI-Korean Journal Database;MEDLINE;ProQuest;SciELO	All	No	#1 AND #2 AND #3	1,987	9-Apr-2024
#8	WoS	Web of Science Core Collection;Current Contents Connect;Derwent Innovations Index;Grants Index;KCI-Korean Journal Database;MEDLINE;ProQuest;SciELO	All	No	#1 AND #2 AND #3 AND #4	176	9-Apr-2024
#9	WoS	Web of Science Core Collection;Current Contents Connect;Derwent Innovations Index;Grants Index;KCI-Korean Journal Database;MEDLINE;ProQuest;SciELO	All	No	#1 AND #2 AND #3 AND #4 AND #5	62	9-Apr-2024
#10	WoS	Web of Science Core Collection;Current Contents Connect;Derwent Innovations Index;Grants Index;KCI-Korean Journal Database;MEDLINE;ProQuest;SciELO	All	No	ALL=(tactical* OR strategy* OR "performance indicator" OR "key performance indicators" OR metric* OR variables* OR statistic* OR indicator*)	6,608,345	9-Apr-2024
#11	WoS	Web of Science Core Collection;Current Contents Connect;Derwent Innovations Index;Grants Index;KCI-Korean Journal Database;MEDLINE;ProQuest;SciELO	All	No	ALL=(physical* OR technical* OR injury* OR conditioning* OR psychological* OR loading* OR time-motion*)	8,157,767	9-Apr-2024
#12	WoS	Web of Science Core Collection;Current Contents Connect;Derwent Innovations Index;Grants Index;KCI-Korean Journal Database;MEDLINE;ProQuest;SciELO	All	No	#1 AND #10 AND #11 AND #4 AND #5	126	9-Apr-2024
#17	WoS	Web of Science Core Collection;Current Contents Connect;Derwent Innovations Index;Grants Index;KCI-Korean Journal Database;MEDLINE;ProQuest;SciELO	All	No	ALL=(soccer OR football NOT "Australian Football" NOT "National Football League" NOT "Gaelic Football") AND ALL=(elite OR top* OR high*) AND ALL=(adult OR male NOT female NOT Youth NOT Young)	4,145	9-Apr-2024
#18	WoS	Web of Science Core Collection;Current Contents Connect;Derwent Innovations Index;Grants Index;KCI-Korean Journal Database;MEDLINE;ProQuest;SciELO	All	No	ALL=((tactic* OR strateg* OR collective OR attack* OR defend* OR defens* OR offens* OR "patterns of play" OR coordinat* OR style OR interaction* OR intra* OR inter* OR coupling* OR organize?action OR tactical-technical))	33,078,046	9-Apr-2024
#19	WoS	Web of Science Core Collection;Current Contents Connect;Derwent Innovations Index;Grants Index;KCI-Korean Journal Database;MEDLINE;ProQuest;SciELO	All	No	ALL=(techni* OR skill* OR technical-tactical OR motor* OR goal* OR pass* OR assist* OR tackl* OR behavio?ral OR head* OR shot* OR ball* OR entr* OR task* OR possession OR zone* OR position* OR space OR "time?space" OR spational?temporal OR physic* OR load* OR conditioning OR injur* OR physiolog* OR strength OR running OR athletic* OR sprint* OR acceleration* OR jump* OR speed* OR work?rate OR wellness OR biomechanic* OR neuromuscular OR fitness OR acute OR chronic OR intensit* OR train* OR movement* OR "activity profile*" OR distance* OR anthropometric* OR recover* OR agility OR psycholog* OR percept* OR action* OR decision*making OR attention OR mental OR emotion* OR role* OR cogniti* OR ecologic* OR visual OR verbal OR emergen* OR complex OR constraint* OR affordances OR enviroment* OR spectator* OR crowd OR situation* OR context*)	44,469,118	9-Apr-2024
#20	WoS	Web of Science Core Collection;Current Contents Connect;Derwent Innovations Index;Grants Index;KCI-Korean Journal Database;MEDLINE;ProQuest;SciELO	All	No	ALL=((outcome OR winn* OR win OR odds OR "odds?ratio" OR expected OR probability))	5,611,364	9-Apr-2024
#21	WoS	Web of Science Core Collection;Current Contents Connect;Derwent Innovations Index;Grants Index;KCI-Korean Journal Database;MEDLINE;ProQuest;SciELO	All	No	ALL=("notational analysis" OR "composite variable" OR “performance analysis” OR “match analysis” OR "game analysis" OR "match statistic*" OR "observational" OR time?motion OR network* OR "performance indicator*" OR "key performance indicator*" OR metric* OR index* OR indicator* OR pattern* OR "sequential analysis" OR measure* OR factor* OR latent* OR ratio* OR match?play OR "match activiti*" OR "multi?dimension*" OR profile OR "multi?factorial" OR coefficient* OR score* OR cluster*)	26,750,845	9-Apr-2024
#22	WoS	Web of Science Core Collection;Current Contents Connect;Derwent Innovations Index;Grants Index;KCI-Korean Journal Database;MEDLINE;ProQuest;SciELO	All	No	#17 AND #18 AND #19 AND #20 AND #21	Error	9-Apr-2024
#23(Population)	WoS	Web of Science Core Collection;Current Contents Connect;Derwent Innovations Index;Grants Index;KCI-Korean Journal Database;MEDLINE;ProQuest;SciELO	Title-Abstract-Keywords	No	TS=((soccer) OR (football))	58,254	9-Apr-2024
#24(Intervention)	WoS	Web of Science Core Collection;Current Contents Connect;Derwent Innovations Index;Grants Index;KCI-Korean Journal Database;MEDLINE;ProQuest;SciELO	Title-Abstract-Keywords	No	TS=((tactical) OR (strategy) OR ("performance indicators") OR ("key performance indicators") OR (metric))	3,693,532	9-Apr-2024
#25(Comparison)	WoS	Web of Science Core Collection;Current Contents Connect;Derwent Innovations Index;Grants Index;KCI-Korean Journal Database;MEDLINE;ProQuest;SciELO	Title-Abstract-Keywords	No	TS=((physical) OR (technical) OR (injury) OR (conditioning) OR (psychological))	9,577,788	9-Apr-2024
#26(Outcome)	WoS	Web of Science Core Collection;Current Contents Connect;Derwent Innovations Index;Grants Index;KCI-Korean Journal Database;MEDLINE;ProQuest;SciELO	Title-Abstract-Keywords	No	TS=((winning) OR ("match outcome") OR (win))	81,015	9-Apr-2024
#27(Study Design)	WoS	Web of Science Core Collection;Current Contents Connect;Derwent Innovations Index;Grants Index;KCI-Korean Journal Database;MEDLINE;ProQuest;SciELO	Title-Abstract-Keywords	No	TS=((“notational analysis”) OR (“performance analysis”) OR (“match analysis”) OR ("observational"))	469,030	9-Apr-2024
#28	WoS	Web of Science Core Collection;Current Contents Connect;Derwent Innovations Index;Grants Index;KCI-Korean Journal Database;MEDLINE;ProQuest;SciELO	Title-Abstract-Keywords	No	#23 AND #24	7,635	9-Apr-2024
#29	WoS	Web of Science Core Collection;Current Contents Connect;Derwent Innovations Index;Grants Index;KCI-Korean Journal Database;MEDLINE;ProQuest;SciELO	Title-Abstract-Keywords	No	#23 AND #24 AND #25	4,137	9-Apr-2024
#30	WoS	Web of Science Core Collection;Current Contents Connect;Derwent Innovations Index;Grants Index;KCI-Korean Journal Database;MEDLINE;ProQuest;SciELO	Title-Abstract-Keywords	No	#23 AND #24 AND #25 AND #26	157	9-Apr-2024
#31	WoS	Web of Science Core Collection;Current Contents Connect;Derwent Innovations Index;Grants Index;KCI-Korean Journal Database;MEDLINE;ProQuest;SciELO	Title-Abstract-Keywords	No	#23 AND #24 AND #25 AND #26 AND #27	51	9-Apr-2024

Supplementary File S6
Preliminary queries and search results

Number	Database	Collections	All/TAK	Duplicates	Query_Pubmed_All	Results	Date
#32	WoS	Web of Science Core Collection;Current Contents Connect;Derwent Innovations Index;Grants Index;KCI-Korean Journal Database;MEDLINE;ProQuest;SciELO	Title-Abstract-Keywords	No	TS=((tactical*) OR (strategy*) OR ("performance indicator") OR ("key performance indicators") OR (metric*) OR (variables*) OR (statistic*) OR (indicator*))	6,177,818	9-Apr-2024
#33	WoS	Web of Science Core Collection;Current Contents Connect;Derwent Innovations Index;Grants Index;KCI-Korean Journal Database;MEDLINE;ProQuest;SciELO	Title-Abstract-Keywords	No	TS=((physical*) OR (technical*) OR (injury*) OR (conditioning*) OR (psychological*) OR (loading*) OR (time-motion*))	4,896,758	9-Apr-2024
#34	WoS	Web of Science Core Collection;Current Contents Connect;Derwent Innovations Index;Grants Index;KCI-Korean Journal Database;MEDLINE;ProQuest;SciELO	Title-Abstract-Keywords	No	#23 AND #32 AND #33 AND #26 AND #27	73	9-Apr-2024
#39	WoS	Web of Science Core Collection;Current Contents Connect;Derwent Innovations Index;Grants Index;KCI-Korean Journal Database;MEDLINE;ProQuest;SciELO	Title-Abstract-Keywords	No	TS=(soccer OR football NOT "Australian Football" NOT "National Football League" NOT "Gaelic Football") AND TS=(elite OR top* OR high*) AND TS=(adult OR male NOT female NOT Youth NOT Young)	3,833	9-Apr-2024
#40	WoS	Web of Science Core Collection;Current Contents Connect;Derwent Innovations Index;Grants Index;KCI-Korean Journal Database;MEDLINE;ProQuest;SciELO	Title-Abstract-Keywords	No	TS=((tactic* OR strateg* OR collective OR attack* OR defend* OR defens* OR offens* OR "patterns of play" OR coordinat* OR style OR interaction* OR intra* OR inter* OR coupling* OR organize?action OR tactical-technical))	23,819,571	9-Apr-2024
#41	WoS	Web of Science Core Collection;Current Contents Connect;Derwent Innovations Index;Grants Index;KCI-Korean Journal Database;MEDLINE;ProQuest;SciELO	Title-Abstract-Keywords	No	TS=(techni* OR skill* OR technical-tactical OR motor* OR goal* OR pass* OR assist* OR tackl* OR behavio?ral OR head* OR shot* OR ball* OR entr* OR task* OR possession OR zone* OR position* OR space OR "time?space" OR spational?temporal OR physic* OR load* OR conditioning OR injur* OR physiolog* OR strength OR running OR athletic* OR sprint* OR acceleration* OR jump* OR speed* OR work?rate OR wellness OR biomechanic* OR neuromuscular OR fitness OR acute OR chronic OR intensit* OR train* OR movement* OR "activity profile*" OR distance* OR anthropometric* OR recover* OR agility OR psycholog* OR percept* OR action* OR decision*making OR attention OR mental OR emotion* OR role* OR cogniti* OR ecologic* OR visual OR verbal OR emergen* OR complex OR constraint* OR affordances OR enviroment* OR spectator* OR crowd OR situation* OR context*)	37,969,749	9-Apr-2024
#42	WoS	Web of Science Core Collection;Current Contents Connect;Derwent Innovations Index;Grants Index;KCI-Korean Journal Database;MEDLINE;ProQuest;SciELO	Title-Abstract-Keywords	No	TS=((outcome OR winn* OR win OR odds OR "odds?ratio" OR expected OR probability))	5,406,448	9-Apr-2024
#43	WoS	Web of Science Core Collection;Current Contents Connect;Derwent Innovations Index;Grants Index;KCI-Korean Journal Database;MEDLINE;ProQuest;SciELO	Title-Abstract-Keywords	No	TS=("notational analysis" OR "composite variable" OR "performance analysis" OR "match analysis" OR "game analysis" OR "match statistic*" OR "observational" OR time?motion OR network* OR "performance indicator*" OR "key performance indicator*" OR metric* OR index* OR indicator* OR pattern* OR "sequential analysis" OR measure* OR factor* OR latent* OR ratio* OR match?play OR "match activiti*" OR "multi?dimension*" OR profile OR "multi?factorial" OR coefficient* OR score* OR cluster*)	25,433,386	9-Apr-2024
#44	WoS	Web of Science Core Collection;Current Contents Connect;Derwent Innovations Index;Grants Index;KCI-Korean Journal Database;MEDLINE;ProQuest;SciELO	Title-Abstract-Keywords	No	#39 AND #40 AND #41 AND #42 AND #43	448	9-Apr-2024
S1(Population)	Ebscohost	eBook Collection; eBook Open Access (OA) Collection; PSICODOC; MLA Directory of Periodicals; MathSciNet via EBSCOhost; Teacher Reference Center	All	No	TX((soccer OR football))	58,421	9-Apr-2024
S2(Intervention)	Ebscohost	eBook Collection; eBook Open Access (OA) Collection; PSICODOC; MLA Directory of Periodicals; MathSciNet via EBSCOhost; Teacher Reference Center	All	No	TX(("tactical" OR "strategy" OR "performance indicators" OR "key performance indicators" OR "metric"))	580,013	9-Apr-2024
S3(Comparison)	Ebscohost	eBook Collection; eBook Open Access (OA) Collection; PSICODOC; MLA Directory of Periodicals; MathSciNet via EBSCOhost; Teacher Reference Center	All	No	TX(("physical" OR "technical" OR "injury" OR "conditioning" OR "psychological"))	2,077,115	9-Apr-2024
S4(Outcome)	Ebscohost	eBook Collection; eBook Open Access (OA) Collection; PSICODOC; MLA Directory of Periodicals; MathSciNet via EBSCOhost; Teacher Reference Center	All	No	TX(("winning" OR "match outcome" OR win*))	926,233	9-Apr-2024
S5(Study Design)	Ebscohost	eBook Collection; eBook Open Access (OA) Collection; PSICODOC; MLA Directory of Periodicals; MathSciNet via EBSCOhost; Teacher Reference Center	All	No	TX((“notational analysis” OR “performance analysis” OR “match analysis” OR "observational"))	145,824	9-Apr-2024
S6	Ebscohost	eBook Collection; eBook Open Access (OA) Collection; PSICODOC; MLA Directory of Periodicals; MathSciNet via EBSCOhost; Teacher Reference Center	All	No	S1 AND S2	10,893	9-Apr-2024
S7	Ebscohost	eBook Collection; eBook Open Access (OA) Collection; PSICODOC; MLA Directory of Periodicals; MathSciNet via EBSCOhost; Teacher Reference Center	All	No	S1 AND S2 AND S3	9,041	9-Apr-2024
S8	Ebscohost	eBook Collection; eBook Open Access (OA) Collection; PSICODOC; MLA Directory of Periodicals; MathSciNet via EBSCOhost; Teacher Reference Center	All	No	S1 AND S2 AND S3 AND S4	7,710	9-Apr-2024
S9	Ebscohost	eBook Collection; eBook Open Access (OA) Collection; PSICODOC; MLA Directory of Periodicals; MathSciNet via EBSCOhost; Teacher Reference Center	All	No	S1 AND S2 AND S3 AND S4 AND S5	1,000	9-Apr-2024
S10	Ebscohost	eBook Collection; eBook Open Access (OA) Collection; PSICODOC; MLA Directory of Periodicals; MathSciNet via EBSCOhost; Teacher Reference Center	All	No	TX((tactical* OR strategy* OR "performance indicator" OR "key performance indicators" OR metric* OR variables* OR statistic* OR indicator*))	3,298,830	9-Apr-2024
S11	Ebscohost	eBook Collection; eBook Open Access (OA) Collection; PSICODOC; MLA Directory of Periodicals; MathSciNet via EBSCOhost; Teacher Reference Center	All	No	TX((physical* OR technical* OR injury* OR conditioning* OR psychological* OR loading* OR time-motion*))	2,167,468	9-Apr-2024
S12	Ebscohost	eBook Collection; eBook Open Access (OA) Collection; PSICODOC; MLA Directory of Periodicals; MathSciNet via EBSCOhost; Teacher Reference Center	All	No	S1 AND S10 AND S11 AND S4 AND S5	1,090	9-Apr-2024
S17	Ebscohost	eBook Collection; eBook Open Access (OA) Collection; PSICODOC; MLA Directory of Periodicals; MathSciNet via EBSCOhost; Teacher Reference Center	All	No	TX ((soccer OR football NOT "Australian Football" NOT "National Football League" NOT "Gaelic Football")) AND TX ((elite OR top* OR high*)) AND TX ((adult OR male NOT female NOT Youth NOT Young))	13,364	9-Apr-2024
S18	Ebscohost	eBook Collection; eBook Open Access (OA) Collection; PSICODOC; MLA Directory of Periodicals; MathSciNet via EBSCOhost; Teacher Reference Center	All	No	TX ((tactic* OR strateg* OR collective OR attack* OR defend* OR defens* OR offens* OR "patterns of play" OR coordinat* OR style OR interaction* OR intra* OR inter* OR coupling* OR organize?action OR tactical-technical))	7,522,549	9-Apr-2024

Number	Database	Collections	All/TAK	Duplicates	Query_Pubmed_All	Results	Date
S19	Ebscohost	eBook Collection; eBook Open Access (OA) Collection; PSICODOC; MLA Directory of Periodicals; MathSciNet via EBSCOhost; Teacher Reference Center	All	No	TX (techni* OR skill* OR technical-tactical OR motor* OR goal* OR pass* OR assist* OR tackl* OR behavio?ral OR head* OR shot* OR ball* OR entr* OR task* OR possession OR zone* OR position* OR space OR "time?space" OR spational?temporal OR physic* OR load* OR conditioning OR injur* OR physiolog* OR strength OR running OR athletic* OR sprint* OR acceleration* OR jump* OR speed* OR work?rate OR wellness OR biomechanic* OR neuromuscular OR fitness OR acute OR chronic OR intensit* OR train* OR movement* OR "activity profile*" OR distance* OR anthropometric* OR recover* OR agility OR psycholog* OR percept* OR action* OR decision*making OR attention OR mental OR emotion* OR role* OR cogniti* OR ecologic* OR visual OR verbal OR emergen* OR complex OR constraint* OR affordances OR enviroment* OR spectator* OR crowd OR situation* OR context*)	10,630,209	9-Apr-2024
S20	Ebscohost	eBook Collection; eBook Open Access (OA) Collection; PSICODOC; MLA Directory of Periodicals; MathSciNet via EBSCOhost; Teacher Reference Center	All	No	TX ((outcome OR winn* OR win OR odds OR "odds?ratio" OR expected OR probability))	2,595,700	9-Apr-2024
S21	Ebscohost	eBook Collection; eBook Open Access (OA) Collection; PSICODOC; MLA Directory of Periodicals; MathSciNet via EBSCOhost; Teacher Reference Center	All	No	TX ("notational analysis" OR "composite variable" OR “performance analysis” OR “match analysis” OR "game analysis" OR "match statistic*" OR "observational" OR time?motion OR network* OR "performance indicator*" OR "key performance indicator*" OR metric* OR index* OR indicator* OR pattern* OR "sequential analysis" OR measure* OR factor* OR latent* OR ratio* OR match?play OR "match activiti*" OR "multi?dimension*" OR profile OR "multi?factorial" OR coefficient* OR score* OR cluster*)	6,089,484	9-Apr-2024
S22	Ebscohost	eBook Collection; eBook Open Access (OA) Collection; PSICODOC; MLA Directory of Periodicals; MathSciNet via EBSCOhost; Teacher Reference Center	All	No	#17 AND #18 AND #19 AND #20 AND #21	8,876	9-Apr-2024
S23(Population)	Ebscohost	eBook Collection; eBook Open Access (OA) Collection; PSICODOC; MLA Directory of Periodicals; MathSciNet via EBSCOhost; Teacher Reference Center	Title-Abstract-Keywords	No	(soccer OR football)	33,535	9-Apr-2024
S24(Intervention)	Ebscohost	eBook Collection; eBook Open Access (OA) Collection; PSICODOC; MLA Directory of Periodicals; MathSciNet via EBSCOhost; Teacher Reference Center	Title-Abstract-Keywords	No	("tactical" OR "strategy" OR "performance indicators" OR "key performance indicators" OR "metric")	425,979	9-Apr-2024
S25(Comparison)	Ebscohost	eBook Collection; eBook Open Access (OA) Collection; PSICODOC; MLA Directory of Periodicals; MathSciNet via EBSCOhost; Teacher Reference Center	Title-Abstract-Keywords	No	("physical" OR "technical" OR "injury" OR "conditioning" OR "psychological")	1,558,410	9-Apr-2024
S26(Outcome)	Ebscohost	eBook Collection; eBook Open Access (OA) Collection; PSICODOC; MLA Directory of Periodicals; MathSciNet via EBSCOhost; Teacher Reference Center	Title-Abstract-Keywords	No	("winning" OR "match outcome" OR win*)	420,156	9-Apr-2024
S27(Study Design)	Ebscohost	eBook Collection; eBook Open Access (OA) Collection; PSICODOC; MLA Directory of Periodicals; MathSciNet via EBSCOhost; Teacher Reference Center	Title-Abstract-Keywords	No	(“notational analysis” OR “performance analysis” OR “match analysis” OR "observational")	132,969	9-Apr-2024
S28	Ebscohost	eBook Collection; eBook Open Access (OA) Collection; PSICODOC; MLA Directory of Periodicals; MathSciNet via EBSCOhost; Teacher Reference Center	Title-Abstract-Keywords	No	S23 AND S24	935	9-Apr-2024
S29	Ebscohost	eBook Collection; eBook Open Access (OA) Collection; PSICODOC; MLA Directory of Periodicals; MathSciNet via EBSCOhost; Teacher Reference Center	Title-Abstract-Keywords	No	S23 AND S24 AND S5	461	9-Apr-2024
S30	Ebscohost	eBook Collection; eBook Open Access (OA) Collection; PSICODOC; MLA Directory of Periodicals; MathSciNet via EBSCOhost; Teacher Reference Center	Title-Abstract-Keywords	No	S23 AND S24 AND S25 AND S26	29	9-Apr-2024
S31	Ebscohost	eBook Collection; eBook Open Access (OA) Collection; PSICODOC; MLA Directory of Periodicals; MathSciNet via EBSCOhost; Teacher Reference Center	Title-Abstract-Keywords	No	S23 AND S24 AND S25 AND S27	5	9-Apr-2024
S32	Ebscohost	eBook Collection; eBook Open Access (OA) Collection; PSICODOC; MLA Directory of Periodicals; MathSciNet via EBSCOhost; Teacher Reference Center	Title-Abstract-Keywords	No	(tactical* OR strategy* OR "performance indicator" OR "key performance indicators" OR metric* OR variables* OR statistic* OR indicator*)	2,976,442	9-Apr-2024
S33	Ebscohost	eBook Collection; eBook Open Access (OA) Collection; PSICODOC; MLA Directory of Periodicals; MathSciNet via EBSCOhost; Teacher Reference Center	Title-Abstract-Keywords	No	(physical* OR technical* OR injury* OR conditioning* OR psychological* OR loading* OR time-motion*)	1,627,490	9-Apr-2024
S34	Ebscohost	eBook Collection; eBook Open Access (OA) Collection; PSICODOC; MLA Directory of Periodicals; MathSciNet via EBSCOhost; Teacher Reference Center	Title-Abstract-Keywords	No	S23 AND S32 AND S33 AND S26 AND S27	11	9-Apr-2024
S39	Ebscohost	eBook Collection; eBook Open Access (OA) Collection; PSICODOC; MLA Directory of Periodicals; MathSciNet via EBSCOhost; Teacher Reference Center	Title-Abstract-Keywords	No	(soccer OR football NOT "Australian Football" NOT "National Football League" NOT "Gaelic Football") AND (elite* OR top OR high*) AND (adult OR male NOT female NOT Youth NOT Young)	3,296	9-Apr-2024
S40	Ebscohost	eBook Collection; eBook Open Access (OA) Collection; PSICODOC; MLA Directory of Periodicals; MathSciNet via EBSCOhost; Teacher Reference Center	Title-Abstract-Keywords	No	(tactic* OR strateg* OR collective OR attack* OR defend* OR defens* OR offens* OR "patterns of play" OR coordinat* OR style OR interaction* OR intra* OR inter* OR coupling* OR organize?action OR tactical-technical)	6,170,544	9-Apr-2024
S41	Ebscohost	eBook Collection; eBook Open Access (OA) Collection; PSICODOC; MLA Directory of Periodicals; MathSciNet via EBSCOhost; Teacher Reference Center	Title-Abstract-Keywords	No	(techni* OR skill* OR technical-tactical OR motor* OR goal* OR pass* OR assist* OR tackl* OR behavio?ral OR head* OR shot* OR ball* OR entr* OR task* OR possession OR zone* OR position* OR space OR "time?space" OR spational?temporal OR physic* OR load* OR conditioning OR injur* OR physiolog* OR strength OR running OR athletic* OR sprint* OR acceleration* OR jump* OR speed* OR work?rate OR wellness OR biomechanic* OR neuromuscular OR fitness OR acute OR chronic OR intensit* OR train* OR movement* OR ""activity profile*"" OR distance* OR anthropometric* OR recover* OR agility OR psycholog* OR percept* OR action* OR decision*making OR attention OR mental OR emotion* OR role* OR cogniti* OR ecologic* OR visual OR verbal OR emergen* OR complex OR constraint* OR affordances OR enviroment* OR spectator* OR crowd OR situation* OR context*)	9,330,309	9-Apr-2024
S42	Ebscohost	eBook Collection; eBook Open Access (OA) Collection; PSICODOC; MLA Directory of Periodicals; MathSciNet via EBSCOhost; Teacher Reference Center	Title-Abstract-Keywords	No	(outcome OR winn* OR win OR odds OR "odds?ratio" OR expected OR probability)	2,176,801	9-Apr-2024
S43	Ebscohost	eBook Collection; eBook Open Access (OA) Collection; PSICODOC; MLA Directory of Periodicals; MathSciNet via EBSCOhost; Teacher Reference Center	Title-Abstract-Keywords	No	(“notational analysis” OR "composite variable" OR “performance analysis” OR “match analysis” OR "game analysis" OR "match statistic*" OR "observational" OR time?motion OR network* OR "performance indicator*" OR "key performance indicator*" OR metric* OR index* OR indicator* OR pattern* OR "sequential analysis" OR measure* OR factor* OR latent* OR ratio* OR match?play OR "match activiti*" OR "multi?dimension*" OR profile OR "multi?factorial" OR coefficient* OR score* OR cluster*)	5,452,509	9-Apr-2024

Supplementary File S6
Preliminary queries and search results

Number	Database	Collections	All/TAK	Duplicates	Query_Pubmed_All	Results	Date
S44	Ebscohost	eBook Collection; eBook Open Access (OA) Collection; PSICODOC; MLA Directory of Periodicals; MathSciNet via EBSCOhost; Teacher Reference Center	Title-Abstract-Keywords	No	S39 AND S40 AND S41 AND S42 AND S43	585	9-Apr-2024
S44	Ebscohost	eBook Collection; eBook Open Access (OA) Collection; PSICODOC; MLA Directory of Periodicals; MathSciNet via EBSCOhost; Teacher Reference Center	Title-Abstract-Keywords	Yes	S39 AND S40 AND S41 AND S42 AND S43	580	9-Apr-2024
#1	Pubmed	Pubmed	Title	No	(soccer[Title] OR football[Title]) NOT "Australian Rules Football"[Title] NOT "Australian Football League"[Title] NOT "American Football"[Title] NOT "National Football League"[Title] NOT "Gaelic Football"[Title] NOT rugby[Title] NOT basketball[Title] NOT handball[Title] NOT volleyball[Title] NOT indoor[Title]	13,083	5-Jul-2024
#2	Pubmed	Pubmed	Title	No	(elite*[Title] OR professional*[Title] OR association[Title]) NOT former[Title] NOT retired*[Title] NOT referee*[Title] NOT amateur*[Title] NOT academ*[Title]	412,554	5-Jul-2024
#3	Pubmed	Pubmed	Title	No	male*[Title] NOT female*[Title] NOT wom?n[Title]	130,970	5-Jul-2024
#4	Pubmed	Pubmed	Title	No	adult*[Title] NOT academ*[Title] NOT youth[Title] NOT junior*[Title] NOT young*[Title] NOT colleg*[Title] NOT adolescent*[Title] NOT universit*[Title] NOT under-1?[Title] NOT female*[Title] NOT wom?n[Title]	416,441	5-Jul-2024
#5	Pubmed	Pubmed	Title	No	#1 AND #2	3,385	5-Jul-2024
#6	Pubmed	Pubmed	Title	No	#1 AND #3	844	5-Jul-2024
#7	Pubmed	Pubmed	Title	No	#1 AND #4	73	5-Jul-2024
#8	Pubmed	Pubmed	Title	No	#1 AND #2 AND #3	316	5-Jul-2024
#9	Pubmed	Pubmed	Title	No	#1 AND #2 AND #4	16	5-Jul-2024
#10	Pubmed	Pubmed	Title	No	#1 AND #3 AND #4	16	5-Jul-2024
#11	Pubmed	Pubmed	Title	No	#1 AND #2 AND #3 AND #4	4	5-Jul-2024
#12(Population)	Pubmed	Pubmed	Title	No	(soccer[Title] OR football[Title]) AND (elite*[Title] OR professional*[Title] OR association[Title]) NOT "Australian Rules Football"[Title] NOT "Australian Football League"[Title] NOT "American Football"[Title] NOT "National Football League"[Title] NOT "Gaelic Football"[Title] NOT rugby[Title] NOT basketball[Title] NOT handball[Title] NOT volleyball[Title] NOT indoor[Title] NOT former[Title] NOT retired*[Title] NOT referee*[Title] NOT amateur*[Title] NOT academ*[Title] NOT youth[Title] NOT junior*[Title] NOT young*[Title] NOT colleg*[Title] NOT adolescent*[Title] NOT universit*[Title] NOT under-1?[Title] NOT female*[Title] NOT wom?n[Title]	2,537	5-Jul-2024
#13(Intervention, Comparison)	Pubmed	Pubmed	Title	No	(intervention*[Title] OR decision*[Title] OR instruction*[Title] OR formation*[Title] OR strateg*[Title] OR substitut*[Title] OR program*[Title] OR change*[Title] OR constraint*[Title] OR method*[Title] OR practice*[Title] OR training[Title] OR coach*[Title] OR adjust*[Title] OR condition*[Title] OR protocol*[Title] OR load*[Title] OR warm-up*[Title] OR exercise*[Title] OR position*[Title] OR prevention*[Title] OR preparation*[Title] OR context*[Title] OR situation*[Title] OR half*[Title] OR halves[Title] OR match*[Title] OR game*[Title])	3,541,046	5-Jul-2024
#14(Outcome)	Pubmed	Pubmed	Title	No	(outcome*[Title] OR winn*[Title] OR win[Title] OR won[Title] OR lose[Title] OR loss[Title] OR losing[Title] OR victor*[Title] OR odds[Title] OR expect*[Title] OR probabili*[Title] OR result*[Title] OR success[Title] OR discriminat*[Title] OR score*[Title] OR action*[Title] OR metric*[Title] OR indicator*[Title] OR statistic*[Title] OR factor*[Title] OR rank*[Title] OR stand*[Title] OR goal*[Title] OR points[Title] OR performance*[Title] OR effect*[Title])	4,968,345	5-Jul-2024
#15(Study Design)	Pubmed	Pubmed	Title	No	("notational analysis"[Title] OR "performance analysis"[Title] OR "match analysis"[Title] OR "game analysis"[Title] OR observation*[Title] OR cross-sectional[Title] OR cohort[Title] OR case-control[Title] OR longitudinal*[Title] OR analytics[Title] OR "machine learning"[Title] OR predict*[Title] OR classif*[Title]) NOT review[Title] NOT "meta-analysis"[Title] NOT synthesis[Title] NOT experimental[Title]	1,141,266	5-Jul-2024
#16	Pubmed	Pubmed	Title	No	#12 AND #13	1,072	5-Jul-2024
#17	Pubmed	Pubmed	Title	No	#12 AND #14	741	5-Jul-2024
#18	Pubmed	Pubmed	Title	No	#12 AND #15	177	5-Jul-2024
#19	Pubmed	Pubmed	Title	No	#12 AND #13 AND #14	416	5-Jul-2024
#20	Pubmed	Pubmed	Title	No	#12 AND #13 AND #15	57	5-Jul-2024
#21	Pubmed	Pubmed	Title	No	#12 AND #14 AND #15	42	5-Jul-2024
#22	Pubmed	Pubmed	Title	No	#12 AND #13 AND #14 AND #15	17	5-Jul-2024
#23	Pubmed	Pubmed	Title-Abstract-Keywords	No	(soccer[Title/Abstract] OR football[Title/Abstract]) NOT "Australian Rules Football"[Title/Abstract] NOT "Australian Football League"[Title/Abstract] NOT "American Football"[Title/Abstract] NOT "National Football League"[Title/Abstract] NOT "Gaelic Football"[Title/Abstract] NOT rugby[Title/Abstract] NOT basketball[Title/Abstract] NOT handball[Title/Abstract] NOT volleyball[Title/Abstract] NOT indoor[Title/Abstract]	18,261	5-Jul-2024
#24	Pubmed	Pubmed	Title-Abstract-Keywords	No	(elite*[Title/Abstract] OR professional*[Title/Abstract] OR association[Title/Abstract]) NOT former[Title/Abstract] NOT retired*[Title/Abstract] NOT referee*[Title/Abstract] NOT amateur*[Title/Abstract] NOT academ*[Title/Abstract]	1,902,618	5-Jul-2024
#25	Pubmed	Pubmed	Title-Abstract-Keywords	No	male*[Title/Abstract] NOT female*[Title/Abstract] NOT wom?n[Title/Abstract]	873,455	5-Jul-2024
#26	Pubmed	Pubmed	Title-Abstract-Keywords	No	adult*[Title/Abstract] NOT academ*[Title/Abstract] NOT youth[Title/Abstract] NOT junior*[Title/Abstract] NOT young*[Title/Abstract] NOT colleg*[Title/Abstract] NOT adolescent*[Title/Abstract] NOT universit*[Title/Abstract] NOT under-1?[Title/Abstract] NOT female*[Title/Abstract] NOT wom?n[Title/Abstract]	1,179,699	5-Jul-2024
#27	Pubmed	Pubmed	Title-Abstract-Keywords	No	#23 AND #24	6,079	5-Jul-2024
#28	Pubmed	Pubmed	Title-Abstract-Keywords	No	#23 AND #25	4,040	5-Jul-2024
#29	Pubmed	Pubmed	Title-Abstract-Keywords	No	#23 AND #26	328	5-Jul-2024
#30	Pubmed	Pubmed	Title-Abstract-Keywords	No	#23 AND #24 AND #25	1,579	5-Jul-2024
#31	Pubmed	Pubmed	Title-Abstract-Keywords	No	#23 AND #24 AND #26	94	5-Jul-2024
#32	Pubmed	Pubmed	Title-Abstract-Keywords	No	#23 AND #25 AND #26	123	5-Jul-2024
#33	Pubmed	Pubmed	Title-Abstract-Keywords	No	#23 AND #24 AND #25 AND #26	43	5-Jul-2024

Supplementary File S6
Preliminary queries and search results

Number	Database	Collections	All/TAK	Duplicates	Query_Pubmed_All	Results	Date
#34(Population)	Pubmed	Pubmed	Title-Abstract-Keywords	No	(soccer[Title/Abstract] OR football[Title/Abstract]) AND (elite*[Title/Abstract] OR professional*[Title/Abstract] OR association[Title/Abstract]) NOT "Australian Rules Football"[Title/Abstract] NOT "Australian Football League"[Title/Abstract] NOT "American Football"[Title/Abstract] NOT "National Football League"[Title/Abstract] NOT "Gaelic Football"[Title/Abstract] NOT rugby[Title/Abstract] NOT basketball[Title/Abstract] NOT handball[Title/Abstract] NOT volleyball[Title/Abstract] NOT indoor[Title/Abstract] NOT former[Title/Abstract] NOT retired*[Title/Abstract] NOT referee*[Title/Abstract] NOT amateur*[Title/Abstract] NOT academ*[Title/Abstract] NOT youth[Title/Abstract] NOT junior*[Title/Abstract] NOT young*[Title/Abstract] NOT colleg*[Title/Abstract] NOT adolescent*[Title/Abstract] NOT universit*[Title/Abstract] NOT under-1?[Title/Abstract] NOT female*[Title/Abstract] NOT wom?n[Title/Abstract]	3,794	5-Jul-2024
#35(Intervention, Comparison)	Pubmed	Pubmed	Title-Abstract-Keywords	No	(intervention*[Title/Abstract] OR decision*[Title/Abstract] OR instruction*[Title/Abstract] OR formation*[Title/Abstract] OR strateg*[Title/Abstract] OR substitut*[Title/Abstract] OR program*[Title/Abstract] OR change*[Title/Abstract] OR constraint*[Title/Abstract] OR method*[Title/Abstract] OR practice*[Title/Abstract] OR training[Title/Abstract] OR coach*[Title/Abstract] OR adjust*[Title/Abstract] OR condition*[Title/Abstract] OR protocol*[Title/Abstract] OR load*[Title/Abstract] OR warm-up*[Title/Abstract] OR exercise*[Title/Abstract] OR position*[Title/Abstract] OR prevention*[Title/Abstract] OR preparation*[Title/Abstract] OR context*[Title/Abstract] OR situation*[Title/Abstract] OR half*[Title/Abstract] OR halves[Title/Abstract] OR match*[Title/Abstract] OR game*[Title/Abstract])	18,245,066	5-Jul-2024
#36(Outcome)	Pubmed	Pubmed	Title-Abstract-Keywords	No	(outcome*[Title/Abstract] OR winn*[Title/Abstract] OR win[Title/Abstract] OR won[Title/Abstract] OR lose[Title/Abstract] OR loss[Title/Abstract] OR losing[Title/Abstract] OR victor*[Title/Abstract] OR odds[Title/Abstract] OR expect*[Title/Abstract] OR probabili*[Title/Abstract] OR result*[Title/Abstract] OR success[Title/Abstract] OR discriminat*[Title/Abstract] OR score*[Title/Abstract] OR action*[Title/Abstract] OR metric*[Title/Abstract] OR indicator*[Title/Abstract] OR statistic*[Title/Abstract] OR factor*[Title/Abstract] OR rank*[Title/Abstract] OR stand*[Title/Abstract] OR goal*[Title/Abstract] OR points[Title/Abstract] OR performance*[Title/Abstract] OR effect*[Title/Abstract])	21,132,487	5-Jul-2024
#37(Study Design)	Pubmed	Pubmed	Title-Abstract-Keywords	No	(("notational analysis"[Title/Abstract] OR "performance analysis"[Title/Abstract] OR "match analysis"[Title/Abstract] OR "game analysis"[Title/Abstract] OR observation*[Title/Abstract] OR cross-sectional[Title/Abstract] OR cohort[Title/Abstract] OR case-control[Title/Abstract] OR longitudinal*[Title/Abstract] OR analytics[Title/Abstract] OR "machine learning"[Title/Abstract] OR predict*[Title/Abstract] OR classif*[Title/Abstract]) NOT review[Title/Abstract] NOT "meta-analysis"[Title/Abstract] NOT synthesis[Title/Abstract] NOT experimental[Title/Abstract])	4,475,807	5-Jul-2024
#38	Pubmed	Pubmed	Title-Abstract-Keywords	No	#34 AND #35	3,419	5-Jul-2024
#39	Pubmed	Pubmed	Title-Abstract-Keywords	No	#34 AND #36	3,340	5-Jul-2024
#40	Pubmed	Pubmed	Title-Abstract-Keywords	No	#34 AND #37	1,208	5-Jul-2024
#41	Pubmed	Pubmed	Title-Abstract-Keywords	No	#34 AND #35 AND #36	3,150	5-Jul-2024
#42	Pubmed	Pubmed	Title-Abstract-Keywords	No	#34 AND #35 AND #37	1,149	5-Jul-2024
#43	Pubmed	Pubmed	Title-Abstract-Keywords	No	#34 AND #36 AND #37	1,169	5-Jul-2024
#44	Pubmed	Pubmed	Title-Abstract-Keywords	No	#34 AND #35 AND #36 AND #37	1,118	5-Jul-2024
#45	Pubmed	Pubmed	Combined	No	#12 AND #35 AND #36 AND #37	807	5-Jul-2024
#46	Pubmed	Pubmed	Combined	No	#12 AND #35 AND #14 AND #37	259	5-Jul-2024
#1	WoS	Web of Science Core Collection;Current Contents Connect;Derwent Innovations Index;Grants Index;KCI-Korean Journal Database;MEDLINE;ProQuest;SciELO	Title	No	TI=((soccer OR football) NOT "Australian Rules Football" NOT "Australian Football League" NOT "American Football" NOT "National Football League" NOT "Gaelic Football" NOT rugby NOT basketball NOT handball NOT volleyball NOT indoor)	41,922	6-Jul-2024
#2	WoS	Web of Science Core Collection;Current Contents Connect;Derwent Innovations Index;Grants Index;KCI-Korean Journal Database;MEDLINE;ProQuest;SciELO	Title	No	TI=((elite* OR professional* OR association) NOT former NOT retired* NOT referee* NOT amateur* NOT academ*)	917,131	6-Jul-2024
#3	WoS	Web of Science Core Collection;Current Contents Connect;Derwent Innovations Index;Grants Index;KCI-Korean Journal Database;MEDLINE;ProQuest;SciELO	Title	No	TI=(male* NOT female* NOT wom?n)	274,233	6-Jul-2024
#4	WoS	Web of Science Core Collection;Current Contents Connect;Derwent Innovations Index;Grants Index;KCI-Korean Journal Database;MEDLINE;ProQuest;SciELO	Title	No	TI=(adult* NOT academ* NOT youth NOT junior* NOT young* NOT colleg* NOT adolescent* NOT universit* NOT under-1? NOT female* NOT wom?n)	694,451	6-Jul-2024
#5	WoS	Web of Science Core Collection;Current Contents Connect;Derwent Innovations Index;Grants Index;KCI-Korean Journal Database;MEDLINE;ProQuest;SciELO	Title	No	#1 AND #2	6,474	6-Jul-2024
#6	WoS	Web of Science Core Collection;Current Contents Connect;Derwent Innovations Index;Grants Index;KCI-Korean Journal Database;MEDLINE;ProQuest;SciELO	Title	No	#1 AND #3	1,278	6-Jul-2024
#7	WoS	Web of Science Core Collection;Current Contents Connect;Derwent Innovations Index;Grants Index;KCI-Korean Journal Database;MEDLINE;ProQuest;SciELO	Title	No	#1 AND #4	138	6-Jul-2024
#8	WoS	Web of Science Core Collection;Current Contents Connect;Derwent Innovations Index;Grants Index;KCI-Korean Journal Database;MEDLINE;ProQuest;SciELO	Title	No	#1 AND #2 AND #3	414	6-Jul-2024
#9	WoS	Web of Science Core Collection;Current Contents Connect;Derwent Innovations Index;Grants Index;KCI-Korean Journal Database;MEDLINE;ProQuest;SciELO	Title	No	#1 AND #2 AND #4	28	6-Jul-2024
#10	WoS	Web of Science Core Collection;Current Contents Connect;Derwent Innovations Index;Grants Index;KCI-Korean Journal Database;MEDLINE;ProQuest;SciELO	Title	No	#1 AND #3 AND #4	26	6-Jul-2024

Supplementary File S6
Preliminary queries and search results

Number	Database	Collections	All/TAK	Duplicates	Query_Pubmed_All	Results	Date
#11	WoS	Web of Science Core Collection;Current Contents Connect;Derwent Innovations Index;Grants Index;KCI-Korean Journal Database;MEDLINE;ProQuest;SciELO	Title	No	#1 AND #2 AND #3 AND #4	7	6-Jul-2024
#12(Population)	WoS	Web of Science Core Collection;Current Contents Connect;Derwent Innovations Index;Grants Index;KCI-Korean Journal Database;MEDLINE;ProQuest;SciELO	Title	No	TI=((soccer OR football) AND (elite* OR professional* OR association) NOT "Australian Rules Football" NOT "Australian Football League" NOT "American Football" NOT "National Football League" NOT "Gaelic Football" NOT rugby NOT basketball NOT handball NOT volleyball NOT indoor NOT former NOT retired* NOT referee* NOT amateur* NOT academ* NOT youth NOT junior* NOT young* NOT colleg* NOT adolescent* NOT universit* NOT under-1? NOT female* NOT wom?n)	4,910	6-Jul-2024
#13(Intervention, Comparison)	WoS	Web of Science Core Collection;Current Contents Connect;Derwent Innovations Index;Grants Index;KCI-Korean Journal Database;MEDLINE;ProQuest;SciELO	Title	No	TI=((intervention* OR decision* OR instruction* OR formation* OR strateg* OR substitut* OR program* OR change* OR constraint* OR method* OR practice* OR training OR coach* OR adjust* OR condition* OR protocol* OR load* OR warm-up* OR exercise* OR position* OR prevention* OR preparation* OR context* OR situation* OR half* OR halves OR match* OR game*))	16,289,276	6-Jul-2024
#14(Outcome)	WoS	Web of Science Core Collection;Current Contents Connect;Derwent Innovations Index;Grants Index;KCI-Korean Journal Database;MEDLINE;ProQuest;SciELO	Title	No	TI=((outcome* OR winn* OR win OR won OR lose OR loss OR losing OR victor* OR odds OR expect* OR probabili* OR result* OR success OR discriminat* OR score* OR action* OR metric* OR indicator* OR statistic* OR factor* OR rank* OR stand* OR goal* OR points OR performance* OR effect*))	14,406,602	6-Jul-2024
#15(Study Design)	WoS	Web of Science Core Collection;Current Contents Connect;Derwent Innovations Index;Grants Index;KCI-Korean Journal Database;MEDLINE;ProQuest;SciELO	Title	No	TI=(("notational analysis" OR "performance analysis" OR "match analysis" OR "game analysis" OR observation* OR cross-sectional OR cohort OR case-control OR longitudinal* OR analytics OR "machine learning" OR predict* OR classif*) NOT review NOT "meta-analysis" NOT synthesis NOT experimental)	2,998,759	6-Jul-2024
#16	WoS	Web of Science Core Collection;Current Contents Connect;Derwent Innovations Index;Grants Index;KCI-Korean Journal Database;MEDLINE;ProQuest;SciELO	Title	No	#12 AND #13	1,788	6-Jul-2024
#17	WoS	Web of Science Core Collection;Current Contents Connect;Derwent Innovations Index;Grants Index;KCI-Korean Journal Database;MEDLINE;ProQuest;SciELO	Title	No	#12 AND #14	1,365	6-Jul-2024
#18	WoS	Web of Science Core Collection;Current Contents Connect;Derwent Innovations Index;Grants Index;KCI-Korean Journal Database;MEDLINE;ProQuest;SciELO	Title	No	#12 AND #15	262	6-Jul-2024
#19	WoS	Web of Science Core Collection;Current Contents Connect;Derwent Innovations Index;Grants Index;KCI-Korean Journal Database;MEDLINE;ProQuest;SciELO	Title	No	#12 AND #13 AND #14	625	6-Jul-2024
#20	WoS	Web of Science Core Collection;Current Contents Connect;Derwent Innovations Index;Grants Index;KCI-Korean Journal Database;MEDLINE;ProQuest;SciELO	Title	No	#12 AND #13 AND #15	82	6-Jul-2024
#21	WoS	Web of Science Core Collection;Current Contents Connect;Derwent Innovations Index;Grants Index;KCI-Korean Journal Database;MEDLINE;ProQuest;SciELO	Title	No	#12 AND #14 AND #15	75	6-Jul-2024
#22	WoS	Web of Science Core Collection;Current Contents Connect;Derwent Innovations Index;Grants Index;KCI-Korean Journal Database;MEDLINE;ProQuest;SciELO	Title	No	#12 AND #13 AND #14 AND #15	27	6-Jul-2024
#23	WoS	Web of Science Core Collection;Current Contents Connect;Derwent Innovations Index;Grants Index;KCI-Korean Journal Database;MEDLINE;ProQuest;SciELO	Title-Abstract-Keywords	No	TS=((soccer OR football) NOT "Australian Rules Football" NOT "Australian Football League" NOT "American Football" NOT "National Football League" NOT "Gaelic Football" NOT rugby NOT basketball NOT handball NOT volleyball NOT indoor)	72,957	6-Jul-2024
#24	WoS	Web of Science Core Collection;Current Contents Connect;Derwent Innovations Index;Grants Index;KCI-Korean Journal Database;MEDLINE;ProQuest;SciELO	Title-Abstract-Keywords	No	TS=((elite* OR professional* OR association) NOT former NOT retired* NOT referee* NOT amateur* NOT academ*)	4,714,789	6-Jul-2024
#25	WoS	Web of Science Core Collection;Current Contents Connect;Derwent Innovations Index;Grants Index;KCI-Korean Journal Database;MEDLINE;ProQuest;SciELO	Title-Abstract-Keywords	No	TS=(male* NOT female* NOT wom?n)	3,924,149	6-Jul-2024
#26	WoS	Web of Science Core Collection;Current Contents Connect;Derwent Innovations Index;Grants Index;KCI-Korean Journal Database;MEDLINE;ProQuest;SciELO	Title-Abstract-Keywords	No	TS=(adult* NOT academ* NOT youth NOT junior* NOT young* NOT colleg* NOT adolescent* NOT universit* NOT under-1? NOT female* NOT wom?n)	2,286,494	6-Jul-2024
#27	WoS	Web of Science Core Collection;Current Contents Connect;Derwent Innovations Index;Grants Index;KCI-Korean Journal Database;MEDLINE;ProQuest;SciELO	Title-Abstract-Keywords	No	#23 AND #24	18,097	6-Jul-2024
#28	WoS	Web of Science Core Collection;Current Contents Connect;Derwent Innovations Index;Grants Index;KCI-Korean Journal Database;MEDLINE;ProQuest;SciELO	Title-Abstract-Keywords	No	#23 AND #25	12,475	6-Jul-2024
#29	WoS	Web of Science Core Collection;Current Contents Connect;Derwent Innovations Index;Grants Index;KCI-Korean Journal Database;MEDLINE;ProQuest;SciELO	Title-Abstract-Keywords	No	#23 AND #26	2,813	6-Jul-2024
#30	WoS	Web of Science Core Collection;Current Contents Connect;Derwent Innovations Index;Grants Index;KCI-Korean Journal Database;MEDLINE;ProQuest;SciELO	Title-Abstract-Keywords	No	#23 AND #24 AND #25	4,317	6-Jul-2024
#31	WoS	Web of Science Core Collection;Current Contents Connect;Derwent Innovations Index;Grants Index;KCI-Korean Journal Database;MEDLINE;ProQuest;SciELO	Title-Abstract-Keywords	No	#23 AND #24 AND #26	854	6-Jul-2024
#32	WoS	Web of Science Core Collection;Current Contents Connect;Derwent Innovations Index;Grants Index;KCI-Korean Journal Database;MEDLINE;ProQuest;SciELO	Title-Abstract-Keywords	No	#23 AND #25 AND #26	2,083	6-Jul-2024
#33	WoS	Web of Science Core Collection;Current Contents Connect;Derwent Innovations Index;Grants Index;KCI-Korean Journal Database;MEDLINE;ProQuest;SciELO	Title-Abstract-Keywords	No	#23 AND #24 AND #25 AND #26	648	6-Jul-2024
#34(Population)	WoS	Web of Science Core Collection;Current Contents Connect;Derwent Innovations Index;Grants Index;KCI-Korean Journal Database;MEDLINE;ProQuest;SciELO	Title-Abstract-Keywords	No	TS=((soccer OR football) AND (elite* OR professional* OR association) NOT "Australian Rules Football" NOT "Australian Football League" NOT "American Football" NOT "National Football League" NOT "Gaelic Football" NOT rugby NOT basketball NOT handball NOT volleyball NOT indoor NOT former NOT retired* NOT referee* NOT amateur* NOT academ* NOT youth NOT junior* NOT young* NOT colleg* NOT adolescent* NOT universit* NOT under-1? NOT female* NOT wom?n)	9,329	6-Jul-2024

Supplementary File S6
Preliminary queries and search results

Number	Database	Collections	All/TAK	Duplicates	Query_Pubmed_All	Results	Date
#35(Intervention, Comparison)	WoS	Web of Science Core Collection;Current Contents Connect;Derwent Innovations Index;Grants Index;KCI-Korean Journal Database;MEDLINE;ProQuest;SciELO	Title-Abstract-Keywords	No	TS=((intervention* OR decision* OR instruction* OR formation* OR strateg* OR substitut* OR program* OR change* OR constraint* OR method* OR practice* OR training OR coach* OR adjust* OR condition* OR protocol* OR load* OR warm-up* OR exercise* OR position* OR prevention* OR preparation* OR context* OR situation* OR half* OR halves OR match* OR game*))	69,609,890	6-Jul-2024
#36(Outcome)	WoS	Web of Science Core Collection;Current Contents Connect;Derwent Innovations Index;Grants Index;KCI-Korean Journal Database;MEDLINE;ProQuest;SciELO	Title-Abstract-Keywords	No	TS=((outcome* OR winn* OR win OR won OR lose OR loss OR losing OR victor* OR odds OR expect* OR probabili* OR result* OR success OR discriminat* OR score* OR action* OR metric* OR indicator* OR statistic* OR factor* OR rank* OR stand* OR goal* OR points OR performance* OR effect*))	66,349,094	6-Jul-2024
#37(Study Design)	WoS	Web of Science Core Collection;Current Contents Connect;Derwent Innovations Index;Grants Index;KCI-Korean Journal Database;MEDLINE;ProQuest;SciELO	Title-Abstract-Keywords	No	TS=(("notational analysis" OR "performance analysis" OR "match analysis" OR "game analysis" OR observation* OR cross-sectional OR cohort OR case-control OR longitudinal* OR analytics OR "machine learning" OR predict* OR classif*) NOT review NOT "meta-analysis" NOT synthesis NOT experimental)	12,858,678	6-Jul-2024
#38	WoS	Web of Science Core Collection;Current Contents Connect;Derwent Innovations Index;Grants Index;KCI-Korean Journal Database;MEDLINE;ProQuest;SciELO	Title-Abstract-Keywords	No	#34 AND #35	8,143	6-Jul-2024
#39	WoS	Web of Science Core Collection;Current Contents Connect;Derwent Innovations Index;Grants Index;KCI-Korean Journal Database;MEDLINE;ProQuest;SciELO	Title-Abstract-Keywords	No	#34 AND #36	7,716	6-Jul-2024
#40	WoS	Web of Science Core Collection;Current Contents Connect;Derwent Innovations Index;Grants Index;KCI-Korean Journal Database;MEDLINE;ProQuest;SciELO	Title-Abstract-Keywords	No	#34 AND #37	2,239	6-Jul-2024
#41	WoS	Web of Science Core Collection;Current Contents Connect;Derwent Innovations Index;Grants Index;KCI-Korean Journal Database;MEDLINE;ProQuest;SciELO	Title-Abstract-Keywords	No	#34 AND #35 AND #36	7,117	6-Jul-2024
#42	WoS	Web of Science Core Collection;Current Contents Connect;Derwent Innovations Index;Grants Index;KCI-Korean Journal Database;MEDLINE;ProQuest;SciELO	Title-Abstract-Keywords	No	#34 AND #35 AND #37	2,114	6-Jul-2024
#43	WoS	Web of Science Core Collection;Current Contents Connect;Derwent Innovations Index;Grants Index;KCI-Korean Journal Database;MEDLINE;ProQuest;SciELO	Title-Abstract-Keywords	No	#34 AND #36 AND #37	2,129	6-Jul-2024
#44	WoS	Web of Science Core Collection;Current Contents Connect;Derwent Innovations Index;Grants Index;KCI-Korean Journal Database;MEDLINE;ProQuest;SciELO	Title-Abstract-Keywords	No	#34 AND #35 AND #36 AND #37	2,034	6-Jul-2024
#45	WoS	Web of Science Core Collection;Current Contents Connect;Derwent Innovations Index;Grants Index;KCI-Korean Journal Database;MEDLINE;ProQuest;SciELO	Combined	No	#12 AND #35 AND #36 AND #37	1,341	6-Jul-2024
#46	WoS	Web of Science Core Collection;Current Contents Connect;Derwent Innovations Index;Grants Index;KCI-Korean Journal Database;MEDLINE;ProQuest;SciELO	Combined	No	#12 AND #35 AND #14 AND #37	466	6-Jul-2024
S1	Ebscohost	MLA International Bibliography with Full Text, Library, Information Science & Technology Abstracts, CINAHL Plus, GreenFILE, Teacher Reference Center, eBook Collection (EBSCOhost), MathSciNet via EBSCOhost, MLA Directory of Periodicals, PSICODOC, eBook Open Access (OA) Collection (EBSCOhost)	Title	No	TI((soccer OR football) NOT "Australian Rules Football" NOT "Australian Football League" NOT "American Football" NOT "National Football League" NOT "Gaelic Football" NOT rugby NOT basketball NOT handball NOT volleyball NOT indoor)	13,614	6-Jul-2024
S2	Ebscohost	MLA International Bibliography with Full Text, Library, Information Science & Technology Abstracts, CINAHL Plus, GreenFILE, Teacher Reference Center, eBook Collection (EBSCOhost), MathSciNet via EBSCOhost, MLA Directory of Periodicals, PSICODOC, eBook Open Access (OA) Collection (EBSCOhost)	Title	No	TI((elite* OR professional* OR association) NOT former NOT retired* NOT referee* NOT amateur* NOT academ*)	269,019	6-Jul-2024
S3	Ebscohost	MLA International Bibliography with Full Text, Library, Information Science & Technology Abstracts, CINAHL Plus, GreenFILE, Teacher Reference Center, eBook Collection (EBSCOhost), MathSciNet via EBSCOhost, MLA Directory of Periodicals, PSICODOC, eBook Open Access (OA) Collection (EBSCOhost)	Title	No	TI(male* NOT female* NOT wom?n)	34,277	6-Jul-2024
S4	Ebscohost	MLA International Bibliography with Full Text, Library, Information Science & Technology Abstracts, CINAHL Plus, GreenFILE, Teacher Reference Center, eBook Collection (EBSCOhost), MathSciNet via EBSCOhost, MLA Directory of Periodicals, PSICODOC, eBook Open Access (OA) Collection (EBSCOhost)	Title	No	TI(adult* NOT academ* NOT youth NOT junior* NOT young* NOT colleg* NOT adolescent* NOT universit* NOT under-1? NOT female* NOT wom?n)	214,739	6-Jul-2024
S5	Ebscohost	MLA International Bibliography with Full Text, Library, Information Science & Technology Abstracts, CINAHL Plus, GreenFILE, Teacher Reference Center, eBook Collection (EBSCOhost), MathSciNet via EBSCOhost, MLA Directory of Periodicals, PSICODOC, eBook Open Access (OA) Collection (EBSCOhost)	Title	No	S1 AND S2	2,248	6-Jul-2024
S6	Ebscohost	MLA International Bibliography with Full Text, Library, Information Science & Technology Abstracts, CINAHL Plus, GreenFILE, Teacher Reference Center, eBook Collection (EBSCOhost), MathSciNet via EBSCOhost, MLA Directory of Periodicals, PSICODOC, eBook Open Access (OA) Collection (EBSCOhost)	Title	No	S1 AND S3	543	6-Jul-2024
S7	Ebscohost	MLA International Bibliography with Full Text, Library, Information Science & Technology Abstracts, CINAHL Plus, GreenFILE, Teacher Reference Center, eBook Collection (EBSCOhost), MathSciNet via EBSCOhost, MLA Directory of Periodicals, PSICODOC, eBook Open Access (OA) Collection (EBSCOhost)	Title	No	S1 AND S4	43	6-Jul-2024
S8	Ebscohost	MLA International Bibliography with Full Text, Library, Information Science & Technology Abstracts, CINAHL Plus, GreenFILE, Teacher Reference Center, eBook Collection (EBSCOhost), MathSciNet via EBSCOhost, MLA Directory of Periodicals, PSICODOC, eBook Open Access (OA) Collection (EBSCOhost)	Title	No	S1 AND S2 AND S3	190	6-Jul-2024

Supplementary File S6
Preliminary queries and search results

Number	Database	Collections	All/TAK	Duplicates	Query_Pubmed_All	Results	Date
S9	Ebscohost	MLA International Bibliography with Full Text, Library, Information Science & Technology Abstracts, CINAHL Plus, GreenFILE, Teacher Reference Center, eBook Collection (EBSCOhost), MathSciNet via EBSCOhost, MLA Directory of Periodicals, PSICODOC, eBook Open Access (OA) Collection (EBSCOhost)	Title	No	S1 AND S2 AND S4	10	6-Jul-2024
S10	Ebscohost	MLA International Bibliography with Full Text, Library, Information Science & Technology Abstracts, CINAHL Plus, GreenFILE, Teacher Reference Center, eBook Collection (EBSCOhost), MathSciNet via EBSCOhost, MLA Directory of Periodicals, PSICODOC, eBook Open Access (OA) Collection (EBSCOhost)	Title	No	S1 AND S3 AND S4	13	6-Jul-2024
S11	Ebscohost	MLA International Bibliography with Full Text, Library, Information Science & Technology Abstracts, CINAHL Plus, GreenFILE, Teacher Reference Center, eBook Collection (EBSCOhost), MathSciNet via EBSCOhost, MLA Directory of Periodicals, PSICODOC, eBook Open Access (OA) Collection (EBSCOhost)	Title	No	S1 AND S2 AND S3 AND S5	3	6-Jul-2024
S12(Population)	Ebscohost	MLA International Bibliography with Full Text, Library, Information Science & Technology Abstracts, CINAHL Plus, GreenFILE, Teacher Reference Center, eBook Collection (EBSCOhost), MathSciNet via EBSCOhost, MLA Directory of Periodicals, PSICODOC, eBook Open Access (OA) Collection (EBSCOhost)	Title	No	TI((soccer OR football) AND (elite* OR professional* OR association) NOT "Australian Rules Football" NOT “Australian Football League” NOT “American Football” NOT "National Football League" NOT "Gaelic Football" NOT rugby NOT basketball NOT handball NOT volleyball NOT indoor NOT former NOT retired* NOT referee* NOT amateur* NOT academ* NOT youth NOT junior* NOT young* NOT colleg* NOT adolescent* NOT universit* NOT under-1? NOT female* NOT wom?n)	1,644	6-Jul-2024
S13(Intervention, Comparison)	Ebscohost	MLA International Bibliography with Full Text, Library, Information Science & Technology Abstracts, CINAHL Plus, GreenFILE, Teacher Reference Center, eBook Collection (EBSCOhost), MathSciNet via EBSCOhost, MLA Directory of Periodicals, PSICODOC, eBook Open Access (OA) Collection (EBSCOhost)	Title	No	TI((intervention* OR decision* OR instruction* OR formation* OR strateg* OR substitut* OR program* OR change* OR constraint* OR method* OR practice* OR training OR coach* OR adjust* OR condition* OR protocol* OR load* OR warm-up* OR exercise* OR position* OR prevention* OR preparation* OR context* OR situation* OR half* OR halves OR match* OR game*)	2,068,249	6-Jul-2024
S14(Outcome)	Ebscohost	MLA International Bibliography with Full Text, Library, Information Science & Technology Abstracts, CINAHL Plus, GreenFILE, Teacher Reference Center, eBook Collection (EBSCOhost), MathSciNet via EBSCOhost, MLA Directory of Periodicals, PSICODOC, eBook Open Access (OA) Collection (EBSCOhost)	Title	No	TI((outcome* OR winn* OR win OR won OR lose OR loss OR losing OR victor* OR odds OR expect* OR probabili* OR result* OR success OR discriminat* OR score* OR action* OR metric* OR indicator* OR statistic* OR factor* OR rank* OR stand* OR goal* OR points OR performance* OR effect*)	1,993,188	6-Jul-2024
S15(Study Design)	Ebscohost	MLA International Bibliography with Full Text, Library, Information Science & Technology Abstracts, CINAHL Plus, GreenFILE, Teacher Reference Center, eBook Collection (EBSCOhost), MathSciNet via EBSCOhost, MLA Directory of Periodicals, PSICODOC, eBook Open Access (OA) Collection (EBSCOhost)	Title	No	TI(("notational analysis" OR "performance analysis" OR “match analysis” OR "game analysis" OR observation* OR cross-sectional OR cohort OR case-control OR longitudinal* OR analytics OR “machine learning” OR predict* OR classif*) NOT review NOT “meta-analysis” NOT synthesis NOT experimental)	503,870	6-Jul-2024
S16	Ebscohost	MLA International Bibliography with Full Text, Library, Information Science & Technology Abstracts, CINAHL Plus, GreenFILE, Teacher Reference Center, eBook Collection (EBSCOhost), MathSciNet via EBSCOhost, MLA Directory of Periodicals, PSICODOC, eBook Open Access (OA) Collection (EBSCOhost)	Title	No	S12 AND S13	641	6-Jul-2024
S17	Ebscohost	MLA International Bibliography with Full Text, Library, Information Science & Technology Abstracts, CINAHL Plus, GreenFILE, Teacher Reference Center, eBook Collection (EBSCOhost), MathSciNet via EBSCOhost, MLA Directory of Periodicals, PSICODOC, eBook Open Access (OA) Collection (EBSCOhost)	Title	No	S12 AND S14	474	6-Jul-2024
S18	Ebscohost	MLA International Bibliography with Full Text, Library, Information Science & Technology Abstracts, CINAHL Plus, GreenFILE, Teacher Reference Center, eBook Collection (EBSCOhost), MathSciNet via EBSCOhost, MLA Directory of Periodicals, PSICODOC, eBook Open Access (OA) Collection (EBSCOhost)	Title	No	S12 AND S15	111	6-Jul-2024
S19	Ebscohost	MLA International Bibliography with Full Text, Library, Information Science & Technology Abstracts, CINAHL Plus, GreenFILE, Teacher Reference Center, eBook Collection (EBSCOhost), MathSciNet via EBSCOhost, MLA Directory of Periodicals, PSICODOC, eBook Open Access (OA) Collection (EBSCOhost)	Title	No	S12 AND S13 AND S14	242	6-Jul-2024
S20	Ebscohost	MLA International Bibliography with Full Text, Library, Information Science & Technology Abstracts, CINAHL Plus, GreenFILE, Teacher Reference Center, eBook Collection (EBSCOhost), MathSciNet via EBSCOhost, MLA Directory of Periodicals, PSICODOC, eBook Open Access (OA) Collection (EBSCOhost)	Title	No	S12 AND S13 AND S15	32	6-Jul-2024
S21	Ebscohost	MLA International Bibliography with Full Text, Library, Information Science & Technology Abstracts, CINAHL Plus, GreenFILE, Teacher Reference Center, eBook Collection (EBSCOhost), MathSciNet via EBSCOhost, MLA Directory of Periodicals, PSICODOC, eBook Open Access (OA) Collection (EBSCOhost)	Title	No	S12 AND S14 AND S15	27	6-Jul-2024
S22	Ebscohost	MLA International Bibliography with Full Text, Library, Information Science & Technology Abstracts, CINAHL Plus, GreenFILE, Teacher Reference Center, eBook Collection (EBSCOhost), MathSciNet via EBSCOhost, MLA Directory of Periodicals, PSICODOC, eBook Open Access (OA) Collection (EBSCOhost)	Title	No	S12 AND S13 AND S14 AND S15	10	6-Jul-2024
S23	Ebscohost	MLA International Bibliography with Full Text, Library, Information Science & Technology Abstracts, CINAHL Plus, GreenFILE, Teacher Reference Center, eBook Collection (EBSCOhost), MathSciNet via EBSCOhost, MLA Directory of Periodicals, PSICODOC, eBook Open Access (OA) Collection (EBSCOhost)	Title-Abstract-Keywords	No	(soccer OR football) NOT "Australian Rules Football" NOT "Australian Football League" NOT "American Football" NOT "National Football League" NOT "Gaelic Football" NOT rugby NOT basketball NOT handball NOT volleyball NOT indoor	24,550	6-Jul-2024
S24	Ebscohost	MLA International Bibliography with Full Text, Library, Information Science & Technology Abstracts, CINAHL Plus, GreenFILE, Teacher Reference Center, eBook Collection (EBSCOhost), MathSciNet via EBSCOhost, MLA Directory of Periodicals, PSICODOC, eBook Open Access (OA) Collection (EBSCOhost)	Title-Abstract-Keywords	No	(elite* OR professional* OR association) NOT former NOT retired* NOT referee* NOT amateur* NOT academ*	1,654,135	6-Jul-2024

Supplementary File S6
Preliminary queries and search results

Number	Database	Collections	All/TAK	Duplicates	Query_Pubmed_All	Results	Date
S25	Ebscohost	MLA International Bibliography with Full Text, Library, Information Science & Technology Abstracts, CINAHL Plus, GreenFILE, Teacher Reference Center, eBook Collection (EBSCOhost), MathSciNet via EBSCOhost, MLA Directory of Periodicals, PSICODOC, eBook Open Access (OA) Collection (EBSCOhost)	Title-Abstract-Keywords	No	male* NOT female* NOT wom?n	460,188	6-Jul-2024
S26	Ebscohost	MLA International Bibliography with Full Text, Library, Information Science & Technology Abstracts, CINAHL Plus, GreenFILE, Teacher Reference Center, eBook Collection (EBSCOhost), MathSciNet via EBSCOhost, MLA Directory of Periodicals, PSICODOC, eBook Open Access (OA) Collection (EBSCOhost)	Title-Abstract-Keywords	No	adult* NOT academ* NOT youth NOT junior* NOT young* NOT colleg* NOT adolescent* NOT universit* NOT under-1? NOT female* NOT wom?n	408,148	6-Jul-2024
S27	Ebscohost	MLA International Bibliography with Full Text, Library, Information Science & Technology Abstracts, CINAHL Plus, GreenFILE, Teacher Reference Center, eBook Collection (EBSCOhost), MathSciNet via EBSCOhost, MLA Directory of Periodicals, PSICODOC, eBook Open Access (OA) Collection (EBSCOhost)	Title-Abstract-Keywords	No	S23 AND S24	5,499	6-Jul-2024
S28	Ebscohost	MLA International Bibliography with Full Text, Library, Information Science & Technology Abstracts, CINAHL Plus, GreenFILE, Teacher Reference Center, eBook Collection (EBSCOhost), MathSciNet via EBSCOhost, MLA Directory of Periodicals, PSICODOC, eBook Open Access (OA) Collection (EBSCOhost)	Title-Abstract-Keywords	No	S23 AND S25	4,198	6-Jul-2024
S29	Ebscohost	MLA International Bibliography with Full Text, Library, Information Science & Technology Abstracts, CINAHL Plus, GreenFILE, Teacher Reference Center, eBook Collection (EBSCOhost), MathSciNet via EBSCOhost, MLA Directory of Periodicals, PSICODOC, eBook Open Access (OA) Collection (EBSCOhost)	Title-Abstract-Keywords	No	S23 AND S26	1,341	6-Jul-2024
S30	Ebscohost	MLA International Bibliography with Full Text, Library, Information Science & Technology Abstracts, CINAHL Plus, GreenFILE, Teacher Reference Center, eBook Collection (EBSCOhost), MathSciNet via EBSCOhost, MLA Directory of Periodicals, PSICODOC, eBook Open Access (OA) Collection (EBSCOhost)	Title-Abstract-Keywords	No	S23 AND S24 AND S25	1,562	6-Jul-2024
S31	Ebscohost	MLA International Bibliography with Full Text, Library, Information Science & Technology Abstracts, CINAHL Plus, GreenFILE, Teacher Reference Center, eBook Collection (EBSCOhost), MathSciNet via EBSCOhost, MLA Directory of Periodicals, PSICODOC, eBook Open Access (OA) Collection (EBSCOhost)	Title-Abstract-Keywords	No	S23 AND S24 AND S26	575	6-Jul-2024
S32	Ebscohost	MLA International Bibliography with Full Text, Library, Information Science & Technology Abstracts, CINAHL Plus, GreenFILE, Teacher Reference Center, eBook Collection (EBSCOhost), MathSciNet via EBSCOhost, MLA Directory of Periodicals, PSICODOC, eBook Open Access (OA) Collection (EBSCOhost)	Title-Abstract-Keywords	No	S23 AND S25 AND S26	978	6-Jul-2024
S33	Ebscohost	MLA International Bibliography with Full Text, Library, Information Science & Technology Abstracts, CINAHL Plus, GreenFILE, Teacher Reference Center, eBook Collection (EBSCOhost), MathSciNet via EBSCOhost, MLA Directory of Periodicals, PSICODOC, eBook Open Access (OA) Collection (EBSCOhost)	Title-Abstract-Keywords	No	S23 AND S24 AND S25 AND S26	445	6-Jul-2024
S34(Population)	Ebscohost	MLA International Bibliography with Full Text, Library, Information Science & Technology Abstracts, CINAHL Plus, GreenFILE, Teacher Reference Center, eBook Collection (EBSCOhost), MathSciNet via EBSCOhost, MLA Directory of Periodicals, PSICODOC, eBook Open Access (OA) Collection (EBSCOhost)	Title-Abstract-Keywords	No	(soccer OR football) AND (elite* OR professional* OR association) NOT "Australian Rules Football" NOT “Australian Football League” NOT “American Football” NOT "National Football League" NOT "Gaelic Football" NOT rugby NOT basketball NOT handball NOT volleyball NOT indoor NOT former NOT retired* NOT referee* NOT amateur* NOT academ* NOT youth NOT junior* NOT young* NOT colleg* NOT adolescent* NOT universit* NOT under-1? NOT female* NOT wom?n	2,582	6-Jul-2024
S35(Intervention, Comparison)	Ebscohost	MLA International Bibliography with Full Text, Library, Information Science & Technology Abstracts, CINAHL Plus, GreenFILE, Teacher Reference Center, eBook Collection (EBSCOhost), MathSciNet via EBSCOhost, MLA Directory of Periodicals, PSICODOC, eBook Open Access (OA) Collection (EBSCOhost)	Title-Abstract-Keywords	No	intervention* OR decision* OR instruction* OR formation* OR strateg* OR substitut* OR program* OR change* OR constraint* OR method* OR practice* OR training OR coach* OR adjust* OR condition* OR protocol* OR load* OR warm-up* OR exercise* OR position* OR prevention* OR preparation* OR context* OR situation* OR half* OR halves OR match* OR game*	9,016,073	6-Jul-2024
S36(Outcome)	Ebscohost	MLA International Bibliography with Full Text, Library, Information Science & Technology Abstracts, CINAHL Plus, GreenFILE, Teacher Reference Center, eBook Collection (EBSCOhost), MathSciNet via EBSCOhost, MLA Directory of Periodicals, PSICODOC, eBook Open Access (OA) Collection (EBSCOhost)	Title-Abstract-Keywords	No	outcome* OR winn* OR win OR won OR lose OR loss OR losing OR victor* OR odds OR expect* OR probabili* OR result* OR success OR discriminat* OR score* OR action* OR metric* OR indicator* OR statistic* OR factor* OR rank* OR stand* OR goal* OR points OR performance* OR effect*	9,427,376	6-Jul-2024
S37(Study Design)	Ebscohost	MLA International Bibliography with Full Text, Library, Information Science & Technology Abstracts, CINAHL Plus, GreenFILE, Teacher Reference Center, eBook Collection (EBSCOhost), MathSciNet via EBSCOhost, MLA Directory of Periodicals, PSICODOC, eBook Open Access (OA) Collection (EBSCOhost)	Title-Abstract-Keywords	No	("notational analysis" OR "performance analysis" OR “match analysis” OR "game analysis" OR observation* OR cross-sectional OR cohort OR case-control OR longitudinal* OR analytics OR “machine learning” OR predict* OR classif*) NOT review NOT “meta-analysis” NOT synthesis NOT experimental	2,010,501	6-Jul-2024
S38	Ebscohost	MLA International Bibliography with Full Text, Library, Information Science & Technology Abstracts, CINAHL Plus, GreenFILE, Teacher Reference Center, eBook Collection (EBSCOhost), MathSciNet via EBSCOhost, MLA Directory of Periodicals, PSICODOC, eBook Open Access (OA) Collection (EBSCOhost)	Title-Abstract-Keywords	No	S34 AND S35	1,977	6-Jul-2024
S39	Ebscohost	MLA International Bibliography with Full Text, Library, Information Science & Technology Abstracts, CINAHL Plus, GreenFILE, Teacher Reference Center, eBook Collection (EBSCOhost), MathSciNet via EBSCOhost, MLA Directory of Periodicals, PSICODOC, eBook Open Access (OA) Collection (EBSCOhost)	Title-Abstract-Keywords	No	S34 AND S36	1,820	6-Jul-2024
S40	Ebscohost	MLA International Bibliography with Full Text, Library, Information Science & Technology Abstracts, CINAHL Plus, GreenFILE, Teacher Reference Center, eBook Collection (EBSCOhost), MathSciNet via EBSCOhost, MLA Directory of Periodicals, PSICODOC, eBook Open Access (OA) Collection (EBSCOhost)	Title-Abstract-Keywords	No	S34 AND S37	500	6-Jul-2024

Number	Database	Collections	All/TAK	Duplicates	Query_Pubmed_All	Results	Date
S41	Ebscohost	MLA International Bibliography with Full Text, Library, Information Science & Technology Abstracts, CINAHL Plus, GreenFILE, Teacher Reference Center, eBook Collection (EBSCOhost), MathSciNet via EBSCOhost, MLA Directory of Periodicals, PSICODOC, eBook Open Access (OA) Collection (EBSCOhost)	Title-Abstract-Keywords	No	S34 AND S35 AND S36	1,618	6-Jul-2024
S42	Ebscohost	MLA International Bibliography with Full Text, Library, Information Science & Technology Abstracts, CINAHL Plus, GreenFILE, Teacher Reference Center, eBook Collection (EBSCOhost), MathSciNet via EBSCOhost, MLA Directory of Periodicals, PSICODOC, eBook Open Access (OA) Collection (EBSCOhost)	Title-Abstract-Keywords	No	S34 AND S35 AND S37	454	6-Jul-2024
S43	Ebscohost	MLA International Bibliography with Full Text, Library, Information Science & Technology Abstracts, CINAHL Plus, GreenFILE, Teacher Reference Center, eBook Collection (EBSCOhost), MathSciNet via EBSCOhost, MLA Directory of Periodicals, PSICODOC, eBook Open Access (OA) Collection (EBSCOhost)	Title-Abstract-Keywords	No	S34 AND S36 AND S37	475	6-Jul-2024
S44	Ebscohost	MLA International Bibliography with Full Text, Library, Information Science & Technology Abstracts, CINAHL Plus, GreenFILE, Teacher Reference Center, eBook Collection (EBSCOhost), MathSciNet via EBSCOhost, MLA Directory of Periodicals, PSICODOC, eBook Open Access (OA) Collection (EBSCOhost)	Title-Abstract-Keywords	No	S34 AND S35 AND S36 AND S37	445	6-Jul-2024
S45	Ebscohost	MLA International Bibliography with Full Text, Library, Information Science & Technology Abstracts, CINAHL Plus, GreenFILE, Teacher Reference Center, eBook Collection (EBSCOhost), MathSciNet via EBSCOhost, MLA Directory of Periodicals, PSICODOC, eBook Open Access (OA) Collection (EBSCOhost)	Combined	No	S12 AND S35 AND S36 AND S37	448	6-Jul-2024
S45	Ebscohost	MLA International Bibliography with Full Text, Library, Information Science & Technology Abstracts, CINAHL Plus, GreenFILE, Teacher Reference Center, eBook Collection (EBSCOhost), MathSciNet via EBSCOhost, MLA Directory of Periodicals, PSICODOC, eBook Open Access (OA) Collection (EBSCOhost)	Combined	No	S12 AND S35 AND S14 AND S37	140	6-Jul-2024
#1	Scopus	Scopus	Title	No	TITLE((soccer OR football) AND NOT "Australian Rules Football" AND NOT "Australian Football League" AND NOT "American Football" AND NOT "National Football League" AND NOT "Gaelic Football" AND NOT rugby AND NOT basketball AND NOT handball AND NOT volleyball AND NOT indoor)	33,211	6-Jul-2024
#2	Scopus	Scopus	Title	No	TITLE((elite* OR professional* OR association) AND NOT former AND NOT retired* AND NOT referee* AND NOT amateur* AND NOT academ*)	723,056	6-Jul-2024
#3	Scopus	Scopus	Title	No	TITLE(male* AND NOT female* AND NOT wom?n)	198,264	6-Jul-2024
#4	Scopus	Scopus	Title	No	TITLE(adult* AND NOT academ* AND NOT youth AND NOT junior* AND NOT young* AND NOT colleg* AND NOT adolescent* AND NOT universit* AND NOT under-1? AND NOT female* AND NOT wom?n)	513,754	6-Jul-2024
#5	Scopus	Scopus	Title	No	#1 AND #2	5,796	6-Jul-2024
#6	Scopus	Scopus	Title	No	#1 AND #3	1,132	6-Jul-2024
#7	Scopus	Scopus	Title	No	#1 AND #4	103	6-Jul-2024
#8	Scopus	Scopus	Title	No	#1 AND #2 AND #3	392	6-Jul-2024
#9	Scopus	Scopus	Title	No	#1 AND #2 AND #4	26	6-Jul-2024
#10	Scopus	Scopus	Title	No	#1 AND #3 AND #4	22	6-Jul-2024
#11	Scopus	Scopus	Title	No	#1 AND #2 AND #3 AND #4	7	6-Jul-2024
#12(Population)	Scopus	Scopus	Title	No	TITLE((soccer OR football) AND (elite* OR professional* OR association) AND NOT "Australian Rules Football" AND NOT “Australian Football League” AND NOT “American Football” AND NOT "National Football League" AND NOT "Gaelic Football" AND NOT rugby AND NOT basketball AND NOT handball AND NOT volleyball AND NOT indoor AND NOT former AND NOT retired* AND NOT referee* AND NOT amateur* AND NOT academ* AND NOT youth AND NOT junior* AND NOT young* AND NOT colleg* AND NOT adolescent* AND NOT universit* AND NOT under-1? AND NOT female* AND NOT wom?n)	4,396	6-Jul-2024
#13(Intervention, Comparison)	Scopus	Scopus	Title	No	TITLE(intervention* OR decision* OR instruction* OR formation* OR strateg* OR substitut* OR program* OR change* OR constraint* OR method* OR practice* OR training OR coach* OR adjust* OR condition* OR protocol* OR load* OR warm-up* OR exercise* OR position* OR prevention* OR preparation* OR context* OR situation* OR half* OR halves OR match* OR game*)	10,366,723	6-Jul-2024
#14(Outcome)	Scopus	Scopus	Title	No	TITLE(outcome* OR winn* OR win OR won OR lose OR loss OR losing OR victor* OR odds OR expect* OR probabili* OR result* OR success OR discriminat* OR score* OR action* OR metric* OR indicator* OR statistic* OR factor* OR rank* OR stand* OR goal* OR points OR performance* OR effect*)	11,312,218	6-Jul-2024
#15(Study Design)	Scopus	Scopus	Title	No	TITLE(("notational analysis" OR "performance analysis" OR “match analysis” OR "game analysis" OR observation* OR cross-sectional OR cohort OR case-control OR longitudinal* OR analytics OR “machine learning” OR predict* OR classif*) AND NOT review AND NOT “meta-analysis” AND NOT synthesis AND NOT experimental)	2,556,172	6-Jul-2024
#16	Scopus	Scopus	Title	No	#12 AND #13	1,634	6-Jul-2024
#17	Scopus	Scopus	Title	No	#12 AND #14	1,212	6-Jul-2024
#18	Scopus	Scopus	Title	No	#12 AND #15	253	6-Jul-2024
#19	Scopus	Scopus	Title	No	#12 AND #13 AND #14	579	6-Jul-2024
#20	Scopus	Scopus	Title	No	#12 AND #13 AND #15	83	6-Jul-2024
#21	Scopus	Scopus	Title	No	#12 AND #14 AND #15	70	6-Jul-2024
#22	Scopus	Scopus	Title	No	#12 AND #13 AND #14 AND #15	29	6-Jul-2024
#23	Scopus	Scopus	Title-Abstract-Keywords	No	TITLE-ABS-KEY((soccer OR football) AND NOT "Australian Rules Football" AND NOT "Australian Football League" AND NOT "American Football" AND NOT "National Football League" AND NOT "Gaelic Football" AND NOT rugby AND NOT basketball AND NOT handball AND NOT volleyball AND NOT indoor)	55,369	6-Jul-2024
#24	Scopus	Scopus	Title-Abstract-Keywords	No	TITLE-ABS-KEY((elite* OR professional* OR association) AND NOT former AND NOT retired* AND NOT referee* AND NOT amateur* AND NOT academ*)	4,960,480	6-Jul-2024
#25	Scopus	Scopus	Title-Abstract-Keywords	No	TITLE-ABS-KEY(male* AND NOT female* AND NOT wom?n)	3,749,844	6-Jul-2024
#26	Scopus	Scopus	Title-Abstract-Keywords	No	TITLE-ABS-KEY(adult* AND NOT academ* AND NOT youth AND NOT junior* AND NOT young* AND NOT colleg* AND NOT adolescent* AND NOT universit* AND NOT under-1? AND NOT female* AND NOT wom?n)	2,258,314	6-Jul-2024
#27	Scopus	Scopus	Title-Abstract-Keywords	No	#23 AND #24	14,245	6-Jul-2024
#28	Scopus	Scopus	Title-Abstract-Keywords	No	#23 AND #25	11,360	6-Jul-2024
#29	Scopus	Scopus	Title-Abstract-Keywords	No	#23 AND #26	2,958	6-Jul-2024
#30	Scopus	Scopus	Title-Abstract-Keywords	No	#23 AND #24 AND #25	3,987	6-Jul-2024

Supplementary File S6
Preliminary queries and search results

Number	Database	Collections	All/TAK	Duplicates	Query_Pubmed_All	Results	Date
#31	Scopus	Scopus	Title-Abstract-Keywords	No	#23 AND #24 AND #26	1,009	6-Jul-2024
#32	Scopus	Scopus	Title-Abstract-Keywords	No	#23 AND #25 AND #26	2,368	6-Jul-2024
#33	Scopus	Scopus	Title-Abstract-Keywords	No	#23 AND #24 AND #25 AND #26	825	6-Jul-2024
#34(Population)	Scopus	Scopus	Title-Abstract-Keywords	No	TITLE-ABS-KEY((soccer OR football) AND (elite* OR professional* OR association) AND NOT "Australian Rules Football" AND NOT “Australian Football League” AND NOT “American Football” AND NOT "National Football League" AND NOT "Gaelic Football" AND NOT rugby AND NOT basketball AND NOT handball AND NOT volleyball AND NOT indoor AND NOT former AND NOT retired* AND NOT referee* AND NOT amateur* AND NOT academ* AND NOT youth AND NOT junior* AND NOT young* AND NOT colleg* AND NOT adolescent* AND NOT universit* AND NOT under-1? AND NOT female* AND NOT wom?n)	7,727	6-Jul-2024
#35(Intervention, Comparison)	Scopus	Scopus	Title-Abstract-Keywords	No	TITLE-ABS-KEY(intervention* OR decision* OR instruction* OR formation* OR strateg* OR substitut* OR program* OR change* OR constraint* OR method* OR practice* OR training OR coach* OR adjust* OR condition* OR protocol* OR load* OR warm-up* OR exercise* OR position* OR prevention* OR preparation* OR context* OR situation* OR half* OR halves OR match* OR game*)	53,210,869	6-Jul-2024
#36(Outcome)	Scopus	Scopus	Title-Abstract-Keywords	No	TITLE-ABS-KEY(outcome* OR winn* OR win OR won OR lose OR loss OR losing OR victor* OR odds OR expect* OR probabili* OR result* OR success OR discriminat* OR score* OR action* OR metric* OR indicator* OR statistic* OR factor* OR rank* OR stand* OR goal* OR points OR performance* OR effect*)	58,936,474	6-Jul-2024
#37(Study Design)	Scopus	Scopus	Title-Abstract-Keywords	No	TITLE-ABS-KEY(("notational analysis" OR "performance analysis" OR “match analysis” OR "game analysis" OR observation* OR cross-sectional OR cohort OR case-control OR longitudinal* OR analytics OR “machine learning” OR predict* OR classif*) AND NOT review AND NOT “meta-analysis” AND NOT synthesis AND NOT experimental)	10,975,327	6-Jul-2024
#38	Scopus	Scopus	Title-Abstract-Keywords	No	#34 AND #35	6,546	6-Jul-2024
#39	Scopus	Scopus	Title-Abstract-Keywords	No	#34 AND #36	6,221	6-Jul-2024
#40	Scopus	Scopus	Title-Abstract-Keywords	No	#34 AND #37	1,851	6-Jul-2024
#41	Scopus	Scopus	Title-Abstract-Keywords	No	#34 AND #35 AND #36	5,560	6-Jul-2024
#42	Scopus	Scopus	Title-Abstract-Keywords	No	#34 AND #35 AND #37	1,718	6-Jul-2024
#43	Scopus	Scopus	Title-Abstract-Keywords	No	#34 AND #36 AND #37	1,751	6-Jul-2024
#44	Scopus	Scopus	Title-Abstract-Keywords	No	#34 AND #35 AND #36 AND #37	1,634	6-Jul-2024
#45	Scopus	Scopus	Combined	No	#12 AND #35 AND #36 AND #37	1,246	6-Jul-2024
#46	Scopus	Scopus	Combined	No	#12 AND #35 AND #14 AND #37	440	6-Jul-2024
