# Supplementary material for: Ranking sports science and medicine interventions impacting team performance: a protocol for a systematic review and meta-analysis of observational studies in elite football
Source: BMJ Open Sport Exerc Med. 2024 Sep 13;10(3):e002196. doi: 10.1136/bmjsem-2024-002196 (PMC11404162; doi:10.1136/bmjsem-2024-002196)
Supplement: online supplemental file 7 [file bmjsem-10-3-s007.pdf]

Supplementary Table S7. Final queries for EBSCOhost, Pubmed, Scopus, and Web of Science databases.

Database	Area	Query
EBSCOhost	Population	TI((soccer OR football) AND (elite* OR professional* OR association) NOT "Australian Rules Football" NOT “Australian Football League” NOT “American Football” NOT "National Football League" NOT "Gaelic Football" NOT rugby NOT basketball NOT handball NOT volleyball NOT indoor NOT former NOT retired* NOT referee* NOT amateur* NOT academ* NOT youth NOT junior* NOT young* NOT colleg* NOT adolescent* NOT universit* NOT under-1? NOT female* NOT wom?n) AND
	Intervention/Comparison	(intervention* OR decision* OR instruction* OR formation* OR strateg* OR substitut* OR program* OR change* OR constraint* OR method* OR practice* OR training OR coach* OR adjust* OR condition* OR protocol* OR load* OR warm-up* OR exercise* OR position* OR prevention* OR preparation* OR context* OR situation* OR half* OR halves OR match* OR game*) AND
	Outcome	(outcome* OR winn* OR win OR won OR lose OR loss OR losing OR victor* OR odds OR expect* OR probabili* OR result* OR success OR discriminat* OR score* OR action* OR metric* OR indicator* OR statistic* OR factor* OR rank* OR stand* OR goal* OR points OR performance* OR effect*) AND
	Study Design	("notational analysis" OR "performance analysis" OR “match analysis” OR "game analysis" OR observation* OR cross-sectional OR cohort OR case-control OR longitudinal* OR analytics OR “machine learning” OR predict* OR classif*) NOT review NOT “meta-analysis” NOT synthesis NOT experimental)

Note. Supplementary Table S7 continued on the next page.

Supplementary Table S7. (Continued)

Database	Area	Query
Pubmed	Population	((soccer[Title] OR football[Title]) AND (elite*[Title] OR professional*[Title] OR association[Title]) NOT "Australian Rules Football"[Title] NOT "Australian Football League"[Title] NOT "American Football"[Title] NOT "National Football League"[Title] NOT "Gaelic Football"[Title] NOT rugby[Title] NOT basketball[Title] NOT handball[Title] NOT volleyball[Title] NOT indoor[Title] NOT former[Title] NOT retired*[Title] NOT referee*[Title] NOT amateur*[Title] NOT academ*[Title] NOT youth[Title] NOT junior*[Title] NOT young*[Title] NOT colleg*[Title] NOT adolescent*[Title] NOT universit*[Title] NOT under-1?[Title] NOT female*[Title] NOT wom?n[Title]) AND
	Intervention/Comparison	(intervention*[Title/Abstract] OR decision*[Title/Abstract] OR instruction*[Title/Abstract] OR formation*[Title/Abstract] OR strateg*[Title/Abstract] OR substitut*[Title/Abstract] OR program*[Title/Abstract] OR change*[Title/Abstract] OR constraint*[Title/Abstract] OR method*[Title/Abstract] OR practice*[Title/Abstract] OR training[Title/Abstract] OR coach*[Title/Abstract] OR adjust*[Title/Abstract] OR condition*[Title/Abstract] OR protocol*[Title/Abstract] OR load*[Title/Abstract] OR warm-up*[Title/Abstract] OR exercise*[Title/Abstract] OR position*[Title/Abstract] OR prevention*[Title/Abstract] OR preparation*[Title/Abstract] OR context*[Title/Abstract] OR situation*[Title/Abstract] OR half*[Title/Abstract] OR halves[Title/Abstract] OR match*[Title/Abstract] OR game*[Title/Abstract]) AND
	Outcome	(outcome*[Title/Abstract] OR winn*[Title/Abstract] OR win[Title/Abstract] OR won[Title/Abstract] OR lose[Title/Abstract] OR loss[Title/Abstract] OR losing[Title/Abstract] OR victor*[Title/Abstract] OR odds[Title/Abstract] OR expect*[Title/Abstract] OR probabili*[Title/Abstract] OR result*[Title/Abstract] OR success[Title/Abstract] OR discriminat*[Title/Abstract] OR score*[Title/Abstract] OR action*[Title/Abstract] OR metric*[Title/Abstract] OR indicator*[Title/Abstract] OR statistic*[Title/Abstract] OR factor*[Title/Abstract] OR rank*[Title/Abstract] OR stand*[Title/Abstract] OR goal*[Title/Abstract] OR points[Title/Abstract] OR performance*[Title/Abstract] OR effect*[Title/Abstract]) AND
	Study Design	(("notational analysis"[Title/Abstract] OR "performance analysis"[Title/Abstract] OR "match analysis"[Title/Abstract] OR "game analysis"[Title/Abstract] OR observation*[Title/Abstract] OR cross-sectional[Title/Abstract] OR cohort[Title/Abstract] OR case-control[Title/Abstract] OR longitudinal*[Title/Abstract] OR analytics[Title/Abstract] OR "machine learning"[Title/Abstract] OR predict*[Title/Abstract] OR classif*[Title/Abstract]) NOT review[Title/Abstract] NOT "meta-analysis"[Title/Abstract] NOT synthesis[Title/Abstract] NOT experimental[Title/Abstract])

Note. Supplementary Table S7 continued on the next page.

Supplementary Table S7. (Continued)

Database	Area	Query
Scopus	Population	TITLE((soccer OR football) AND (elite* OR professional* OR association) AND NOT "Australian Rules Football" AND NOT “Australian Football League” AND NOT “American Football” AND NOT "National Football League" AND NOT "Gaelic Football" AND NOT rugby AND NOT basketball AND NOT handball AND NOT volleyball AND NOT indoor AND NOT former AND NOT retired* AND NOT referee* AND NOT amateur* AND NOT academ* AND NOT youth AND NOT junior* AND NOT young* AND NOT colleg* AND NOT adolescent* AND NOT universit* AND NOT under-1? AND NOT female* AND NOT wom?n) AND
	Intervention/Comparison	TITLE-ABS-KEY(intervention* OR decision* OR instruction* OR formation* OR strateg* OR substitut* OR program* OR change* OR constraint* OR method* OR practice* OR training OR coach* OR adjust* OR condition* OR protocol* OR load* OR warm-up* OR exercise* OR position* OR prevention* OR preparation* OR context* OR situation* OR half* OR halves OR match* OR game*) AND
	Outcome	TITLE-ABS-KEY(outcome* OR winn* OR win OR won OR lose OR loss OR losing OR victor* OR odds OR expect* OR probabili* OR result* OR success OR discriminat* OR score* OR action* OR metric* OR indicator* OR statistic* OR factor* OR rank* OR stand* OR goal* OR points OR performance* OR effect*) AND
	Study Design	TITLE-ABS-KEY("notational analysis" OR "performance analysis" OR “match analysis” OR "game analysis" OR observation* OR cross-sectional OR cohort OR case-control OR longitudinal* OR analytics OR “machine learning” OR predict* OR classif*) AND NOT review AND NOT “meta-analysis” AND NOT synthesis AND NOT experimental)

Note. Supplementary Table S7 continued on the next page.

Supplementary Table S7. (Continued)

Database	Area	Query
Web of Science	Population	TI=((soccer OR football) AND (elite* OR professional* OR association) NOT "Australian Rules Football" NOT “Australian Football League” NOT “American Football” NOT "National Football League" NOT "Gaelic Football" NOT rugby NOT basketball NOT handball NOT volleyball NOT indoor NOT former NOT retired* NOT referee* NOT amateur* NOT academ* NOT youth NOT junior* NOT young* NOT colleg* NOT adolescent* NOT universit* NOT under-1? NOT female* NOT wom?n) AND
	Intervention/Comparison	TS=(intervention* OR decision* OR instruction* OR formation* OR strateg* OR substitut* OR program* OR change* OR constraint* OR method* OR practice* OR training OR coach* OR adjust* OR condition* OR protocol* OR load* OR warm-up* OR exercise* OR position* OR prevention* OR preparation* OR context* OR situation* OR half* OR halves OR match* OR game*) AND
	Outcome	TS=(outcome* OR winn* OR win OR won OR lose OR loss OR losing OR victor* OR odds OR expect* OR probabili* OR result* OR success OR discriminat* OR score* OR action* OR metric* OR indicator* OR statistic* OR factor* OR rank* OR stand* OR goal* OR points OR performance* OR effect*) AND
	Study Design	TS=(("notational analysis" OR "performance analysis" OR “match analysis” OR "game analysis" OR observation* OR cross-sectional OR cohort OR case-control OR longitudinal* OR analytics OR “machine learning” OR predict* OR classif*) NOT review NOT “meta-analysis” NOT synthesis NOT experimental)
