# Supplementary material for: Ranking sports science and medicine interventions impacting team performance: a protocol for a systematic review and meta-analysis of observational studies in elite football
Source: BMJ Open Sport Exerc Med. 2024 Sep 13;10(3):e002196. doi: 10.1136/bmjsem-2024-002196 (PMC11404162; doi:10.1136/bmjsem-2024-002196)
Supplement: online supplemental file 8 [file bmjsem-10-3-s008.pdf]

Supplementary Table S8. Data form's fields, scale, descriptions and codes

Group	Field	Data structure or type (scale)	Description	Limited answers and codes[a]
Metadata	Year	String (nominal)	The year the study was published, e.g., 2015.	N/A
	Journal	String (nominal)	The source's name where the study was published, e.g., Apunts Educación Física y Deportes.	The codes are assigned after collection.
Population (P) [b]	Country	String (nominal)	The country where the domestic competition or tournament is played, e.g., England for Premier League.	The codes are assigned after collection.
	Competition	String (nominal)	Tournaments or leagues from which the study sample was collected, e.g., Premier League.	The codes are assigned after collection.
	Year of competition	Integer (cont.)	The year the season of the sample began, e.g., 2015 for the 2015-2016 season.	N/A
	Number of teams' match observations (n)	Integer (cont.)	The number of matches included in the sample, played by one or more teams under study.	N/A
	Number of teams' match observations excluded (n)	Integer (cont.)	The number of matches played by one or more teams excluded from the sample.	N/A
	Reason for exclusion	List (nominal)	Describe the reasons for excluding matches from the sample.	An exclusive label and code are assigned for each after collection.
	Number of teams (n)	Integer (cont.)	The number of teams that generate the match observations.	N/A
	Number of players (n)	Integer (cont.)	The total number of players that generated the match observations, e.g., a player who played one or more times in a match, is counted as one.	N/A
	Number of players observations (n)	Integer (cont.)	The total number of player observations that generated the match observations, e.g., a player who played ten times, is counted as 10.	N/A

Note. Supplementary Table S8 continued on the next page. N/A denotes 'not applicable', and cont. denotes to continuous.

Supplementary Table S8. (Continued)

Group	Field	Data structure or type (scale)	Description	Limited answers and codes[a]
Intervention (I) [b]	Theme	String (nominal)	It consists of the group the term belongs to, e.g., playing style.	An exclusive label and code are assigned for each after collection.
	Term	String (nominal)	It consists of the name of the intervention (or independent variable) used in the study, e.g., attacking style.	An exclusive label and code are assigned for each after collection.
	Definition	String (nominal)	The details that constitute the intervention.	N/A
	Type	String (nominal)	It determines whether an intervention aligns more with sports science (focuses on performance, training, and biomechanics) or sports medicine (concentrates on injury prevention, treatment, and rehabilitation).	1 = Sports Science<br>2 = Sports Medicine<br>3 = Other
	Method	String (nominal)	The area in which the intervention is included.	1 = Conditioning/training programs<br>2 = Nutritional/hydration strategies<br>3 = Psychological techniques<br>4 = Strategical/tactical decisions<br>5 = Training periodisation<br>6 = Injury-related<br>7 = Equipment-related<br>8 = Other
	Frequency	Integer (cont.)	How often was the intervention administered, e.g., weekly, monthly.	N/A
	Duration	Integer (cont.)	The total duration that the intervention was applied, e.g., two weeks.	N/A
	Intensity	Integer (cont.)	The level of effort or load in which the intervention is applied.	1 = Low intensity<br>2 = Moderate intensity<br>3 = Vigorous intensity<br>4 = High intensity<br>5 = Submaximal intensity<br>6 = Maximal intensity

Note. Supplementary Table S8 continued on the next page. N/A denotes ‘not applicable’, and cont. denotes to continuous.

Supplementary Table S8. (Continued)

Group	Field	Data structure or type (scale)	Description	Limited answers and codes[a]
	Match halves	String (nominal)	It consists of the pre-and in-moment phases of the intervention, based on the two equal periods of play in football matches.	1 = Pre-first half<br>2 = In-first half<br>3 = Pre-second half<br>4 = In-second half
	Match period	String (dicho.)	It consists of the moment of the intervention based on the full football matches.	1 = Pre-match<br>2 = In-match<br>3 = Pos-match
	Phase of the game	String (nominal)	It consists of the moments of the game where the intervention was applied, based on moments of play. The offensive phase begins with the gain of ball possession, and the defensive phase begins with the loss of ball possession, which could be by interruption of the game, and ends with ball recovery and, consequently, ball possession [1].	1 = Defensive<br>2 = Offensive<br>3 = Transition attack-defense<br>4 = Transition defense-attack<br>5 = Set-pieces<br>6 = Globally
Outcome (O) [b]	Term	String (nominal)	It consists of the name of the outcome (or dependent variable) used in the study.	An exclusive label and code are assigned for each after collection.
	Definition	String (nominal)	The details that constitute the outcome.	N/A
	Unit	String (nominal)	The metric used to measure the outcome.	An exclusive label and code are assigned for each after collection.
	Type	String (nominal)	It corresponds to the variables that describe success, including goals scored, goals conceded, goal difference, number of wins, number of losses, the difference between wins and losses, categorical and dichotomous match outcomes, team final ranking, number of points, and point difference. Additionally, it includes variables that describe team match effects, such as physical and technical match statistics.	1 = Team success<br>2 = Team match performance<br>3 = Team match effects
	Phase of the game	String (nominal)	Similar to the phase of the game of intervention, but applied to outcomes, e.g., goals scored from corner kicks correspond to set pieces.	1 = Defensive<br>2 = Offensive<br>3 = Transition Attack-Defense<br>4 = Transition Defense-Attack<br>5 = Set-Pieces<br>6 = Globally

Note. Supplementary Table S8 continued on the next page. N/A denotes 'not applicable', and dicho. denotes dichotomous.

Supplementary Table S8. (Continued)

Group	Field	Data structure or type (scale)	Description	Limited answers and codes[a]
Comparisons (C) [b,c]	Intervention 1	String (nominal)	The specific name intervention being studied compared with Intervention 2 for a particular outcome and subgroup.	An exclusive label and code are assigned for each after collection.
	Intervention 2	String (nominal)	The specific name intervention being studied compared with Intervention 1 for a particular outcome and subgroup.	An exclusive label and code are assigned for each after collection.
	Outcome	String (nominal)	The measured result was used to compare Interventions 1 and 2, which were defined similarly to the term in the Outcome group.	An exclusive label and code are assigned for each after collection.
	Subgroup	String (nominal)	A sample stratification of the comparison between Intervention 1 and 2 for a specific outcome, e.g., matches against top and bottom teams.	An exclusive label and code are assigned for each after collection.
	Control variables	List (nominal)	It consists of all constant categorical factors that increase the probability of accurately measuring the independent variable, e.g., contextual factors, team characteristics, or player characteristics.	An exclusive label and code are assigned for each after collection.
	Covariates	List (nominal)	It consists of continuous variables in the analysis to account for variability influencing the outcome.	An exclusive label and code are assigned for each after collection.
	Mean	Integer (cont.)	It is the sum of all data point values divided by their total number.	N/A
	SD	Integer (cont.)	It measures the amount of variation or dispersion in a set of values.	N/A
	Sample size	Integer (cont.)	It refers to the total number of individual observations or data points collected in a study.	N/A
	Proportions	Integer (cont.)	It is a part of the whole represented by fraction or percentage, e.g., matches won out of the total matches played.	N/A
	Total in Proportions	Integer (cont.)	The total number of observations or data points used when calculating proportions, e.g., the proportions of matches won, is the total number of matches played.	N/A
	P-value	Integer (cont.)	The smallest level of significance that rejects the null hypothesis [2].	N/A
	ES value	Integer (cont.)	The quantitative measure of magnitude between interventions [3].	N/A

Note. Supplementary Table S8 continued on the next page. ES = effect size, SD = standard deviation. N/A denotes ‘not applicable’, and cont. denotes to continuous.

Supplementary Table S8. (Continued)

Group	Field	Data structure or type (scale)	Description	Limited answers and codes[a]
	ES 95% CI	Integer (cont.)	Lower- and upper-confidence limits of the values likely to contain the actual value of effect size [2].	N/A
	ES SE	Integer (cont.)	It is a precision measure of the effect size, indicating the variability from the true population value [2].	N/A
	ES reported	String (nominal)	The name of the quantitative measure of magnitude between interventions. The following effect sizes are considered [3]. It includes odds ratio, log odds ratio, Cohen's d, Hedges' g, standardised coefficients, and others.	1 = Odds ratio<br>2 = Log odds ratio<br>3 = Cohen's d<br>4 = Hedges' g<br>5 = Standardised coefficients<br>6 = Other<br>7 = None
	ES type	Integer (cont.)	It categorises the modification of the effect size to account for potential confounders and is limited to crude, i.e., does not account, and adjusted, i.e., accounts [4].	1 = Crude<br>2 = Adjusted
Study Design (S) [b]	Study design type	String (nominal)	The research framework of the study was conducted, and it includes case-control (comparing teams with and without intervention), cross-sectional (analysing data from a team at a one-time point), cohort (following a team over time), and longitudinal (repeated observations over time) Thomas, Nelson [5].	1 = Case-control<br>2 = Cross-sectional<br>3 = Cohort<br>4 = Longitudinal<br>5 = Other
	Instrument name	String (nominal)	The name of the device that researchers used to collect the study data.	N/A
	Instrument type	String (nominal)	A mutually exclusive category for the data collection tool, limited to automatic tracking systems, global positioning systems, databases/websites, notational or observational instruments, surveys, and others.	1 = Semi- or full-automatic tracking systems<br>2 = Global positions systems<br>3 = Databases/websites<br>4 = Notational or observational instruments<br>5 = Survey<br>6 = Other

Note. Supplementary Table S8 continued on the next page. CI = confidence interval, ES = effect size, SE = standard error. N/A denotes 'not applicable', and cont. denotes to continuous.

Supplementary Table S8. (Continued)

Group	Field	Data structure or type (scale)	Description	Limited answers and codes[a]
	Instrument validity	String (nominal)	The degree to which a test is supposed to be measured and the researchers' interpretation and use of that measure [6]. All types of validity are considered, such as content, concurrent, criterion and others [7, 8]. The validity of an instrument is assessed as stated (validity cited or measured in at least one study of the included studies), not stated (validity was not cited or measured in at least one study of the included studies), and unclear (when the previous conditions are not met).	1 = Not stated<br>2 = Unclear<br>3 = Stated
	Instrument inter-reliability	String (nominal)	Determines whether two observers/instruments are consistent, or contrarily, the degree of variation between observers measures of the same group of subjects [9, 10]. The reliability of measures between different instruments or observers is assessed as stated (inter-reliability cited or measured in at least one study of the included studies), not stated (inter-reliability was not cited or measured in at least one study of the included studies), and unclear (when the previous conditions are not met).	1 = Not stated<br>2 = Unclear<br>3 = Stated
	Instrument intra-reliability	String (nominal)	Determines the degree of consistency of measurements performed by the same observer or instrument at different points in time [9, 10]. The reliability of the measures collected by instruments or observers from the same sample in different periods is assessed as stated (intra-reliability cited or measured in at least one study of the included studies), not stated (intra-reliability was not cited or measured in at least one study of the included studies), and unclear (when the previous conditions are not met).	1 = Not stated<br>2 = Unclear<br>3 = Stated
	Data analysis approaches	String (dicho.)	Methods used for analysing data are categorised as either utilising machine learning, e.g., 80-20% training and testing data or statistical modelling techniques, e.g., ANOVA [11].	1 = Statistical modelling<br>2 = Machine learning modelling
	Inferential paradigm	String (dicho.)	The framework for analysing data using either frequentist methods, i.e., do not involve prior distributions, or Bayesian methods, which incorporate prior and posterior distributions [2, 12].	1 = Frequentist<br>2 = Bayesian

Note. Supplementary Table S8 continued on the next page and dicho. denotes dichotomous.

Supplementary Table S8. (Continued)

Group	Field	Data structure or type (scale)	Description	Limited answers and codes[a]
	Statistical tests/ machine learning techniques' name	String (nominal)	The name of the test or technique used in the study to perform the statistical or machine learning analysis, e.g., linear regression [2].	An exclusive label and code are assigned for each after collection.
	Preprocessing/data cleaning description	List (nominal)	It consists of the problems identified and the solutions or tests applied by researchers, including but not limited to issues like distribution, homogeneity, and missing values. For example, [normality test: Kolmogorov-Smirnov test, ...].	An exclusive label and code are assigned for each after collection.
	Model evaluation description	List (nominal)	It comprises the techniques, measures, and values used to assess the model. For example, [cross-validation: accuracy: 80%, …].	An exclusive label and code are assigned for each after collection.
	Machine learning problem type	String (nominal)	It categorises the machine learning task used by studies as either prediction (regression continuous outcomes), classification (assigning data to predefined classes), clustering (grouping data by a measure), or other outside these categories.	1 = Prediction<br>2 = Classification<br>3 = Clustering<br>4 = Other
	Statistical/machine learning analysis type	String (nominal)	The test used in the study is classified into three categories [2]: univariate (data analysis is performed by one variable without causes or relationships, e.g., t-test), bivariate (data analysis is performed to find relationships between two variables, e.g., correlation coefficient), and multivariate (data analysis is performed more than two variables or have more than a single response, e.g. multiple regression).	1 = Univariate<br>2 = Bivariate<br>3 = Multivariate
	Statistical tests/ machine learning techniques methods	String (nominal)	The types of approaches used to perform statistical analyses in a study are categorised by assessing differences (comparisons between groups), associations (relationships between variables), or regression (modelling the relationship between dependent and independent variables) [2].	1 = Differences<br>2 = Associations and correlations<br>3 = Regression

Note.

[a] In each answer, there is the standard option 'not applicable/no information', which marks when there is no information or the information provided cannot be related to the question of not being integrated into one of the categories.

[b] A study may contain multiple rows of data if more than one entity is recorded or collected for at least one field.

[c] The fields are used for each relationship between outcome, interventions and their subgroup in studies, where applicable. For example, 4-4-2 and 4-4-3 interventions were studied with outcome goals, wins, and subgroups of top and bottom opponents. Thus, the mean or odds ratio of goals and wins has to be collected for intervention 4-4-2, 4-4-2 against top opponents, 4-4-2 against bottom opponents, 4-4-3, 4-4-3 against top opponents, and 4-4-3 against bottom opponents.

[1] Barreira D, Garganta J, Prudente J, Anguera MT. Desenvolvimento e validação de um sistema de observação aplicado à fase ofensiva em Futebol: SoccerEye. Rev Port Cienc Desporto. 2012;12(3):32-57.
[2] Montgomery DC, Runger GC. Applied statistics and probability for engineers. New York: John Wiley & Sons, Inc.; 2003.
[3] Ellis PD. The essential guide to effect sizes: statistical power, meta-analysis, and the interpretation of research results. Cambridge: Cambridge University Press; 2010.
[4] Mickey RM. The magnitude of the difference of crude and adjusted log odds ratios. Commun Stat Theory Methods. 1987;16(11):3403-15. https://doi.org/10.1080/03610928708829579
[5] Thomas JR, Nelson JK, Silverman SJ. Research methods in physical activity. 6th ed. Champaign, IL: Human Kinetics; 2011.
[6] Furr RM. Psychometrics : An Introduction. Thousand Oaks: SAGE Publications, Inc; 2021.
[7] Bolarinwa OA. Principles and methods of validity and reliability testing of questionnaires used in social and health science researches. Niger Postgrad Med J. 2015;22(4):195-201. https://doi.org/10.4103/1117-1936.173959
[8] Mokkink LB, Terwee CB, Patrick DL, Alonso J, Stratford PW, Knol DL, et al. The COSMIN study reached international consensus on taxonomy, terminology, and definitions of measurement properties for health-related patient-reported outcomes. J Clin Epidemiol. 2010;63(7):737-45. https://doi.org/10.1016/j.jclinepi.2010.02.006
[9] Koo TK, Li MY. A guideline of selecting and reporting intraclass correlation coefficients for reliability research. J Chiropr Med. 2016;15(2):155-63. https://doi.org/10.1016/j.jcm.2016.02.012
[10] Bruton A, Conway JH, Holgate ST. Reliability: What is it, and how is it measured? Physiother. 2000;86(2):94-9. https://doi.org/https://doi.org/10.1016/S0031-9406(05)61211-4
[11] Bzdok D, Altman N, Krzywinski M. Statistics versus machine learning. Nat Methods. 2018;15(4):233-4. https://doi.org/10.1038/nmeth.4642
[12] Seide SE, Jensen K, Kieser M. A comparison of Bayesian and frequentist methods in random-effects network meta-analysis of binary data. Res Synth Methods. 2020;11(3):363-78. https://doi.org/10.1002/jrsm.1397
