# Supplementary material for: Ranking sports science and medicine interventions impacting team performance: a protocol for a systematic review and meta-analysis of observational studies in elite football
Source: BMJ Open Sport Exerc Med. 2024 Sep 13;10(3):e002196. doi: 10.1136/bmjsem-2024-002196 (PMC11404162; doi:10.1136/bmjsem-2024-002196)
Supplement: online supplemental file 9 [file bmjsem-10-3-s009.pdf]

Supplementary Table S9. Template for characteristics of included studies and summary of findings.

Reference	Sample	Instruments[a]	Intervention[a]	Outcome[a]	Control variables[a]	Statistical tests and key findings[b]
[study citation or ID]	[sample size]<br>[number of teams] ([total of number of observations])<br>[number of players] ([total of number of observations])<br>[competition and season]	[instrument name] ([type])<br><br>[validity and reliability information]	[intervention term] ([metric])	[outcome term] ([metric])	[control variable term] ([metric])	[statistical test] ([method of statistical test], [type of statistical test])<br>[description of key findings with specific formats]

Note. [] denotes the fields to be filled out by the reviewer.

[a]
— The field can be repeated several times.

[b]
— The field is limited to three findings with the greatest Hedges' g (g) or odds ratio (OR) effect size within the same statistical test. If there are more than three statistical tests, only three will be selected based on the greatest effect sizes.

— The following symbols will be used for comparison between interventions' effect size values:
  > denotes greater values between interventions.
  < denotes lower values between interventions.
  ≈ denotes similar values between interventions.
  ↓ denotes a negative relationship between intervention and outcome.
  ↑ denotes a positive relationship between intervention and outcome.

— Bold values for representing statistically significant values ($p<.05$).

— Letters in parentheses to denote the magnitude of effects interpretation: s = small (OR < 1.68; g < 0.2), m = moderate (1.68 ≤ OR < 3.47; 0.2 ≤ g < 0.5), l = large (OR ≥ 6.71; g ≥ 0.8) (1,2)

— The findings are represented in the following formats (inside quotation marks only, and [] denotes the fields to be filled by the reviewer):
  (i) Differences: "[intervention] [symbol of comparison] [intervention]: [outcome] ([magnitude of effects interpretation])", e.g. intervention1 > intervention2: outcome1 (l)
  (ii) Associations and regressions: "[intervention] [symbol of comparison] [outcome]", e.g., intervention1 ↓ outcome1 (s)

— The statistical test method consists of a categorical variable or multiple-choice task with values such as associations, differences, or regression.

— The type of statistical test consists of a categorical variable or multiple-choice task with univariate, bivariate and multivariate values.

(1) Chen H, Cohen P, Chen S. How Big is a Big Odds Ratio? Interpreting the Magnitudes of Odds Ratios in Epidemiological Studies. Communications in Statistics - Simulation and Computation. 2010;39(4):860-4. https://doi.org/10.1080/03610911003650383
(2) Cohen J. Statistical power analysis for the behavioral sciences. 2nd ed. New York: Lawrence Erlbaum Associates; 1988.
