# Supplementary material for: Ranking sports science and medicine interventions impacting team performance: a protocol for a systematic review and meta-analysis of observational studies in elite football
Source: BMJ Open Sport Exerc Med. 2024 Sep 13;10(3):e002196. doi: 10.1136/bmjsem-2024-002196 (PMC11404162; doi:10.1136/bmjsem-2024-002196)
Supplement: online supplemental file 10 [file bmjsem-10-3-s010.pdf]

Supplementary Table S10. Template for interventions' effects on outcomes of included studies.

**Outcome**	**Study** [a]	**[Interventions]**[b]	**[Comparisons]**[c]
[outcome term] ([metric])	[study citation or ID]	[descriptive statistics according to the format]	[effect size] ([%95 confidence of intervals])

Note. [] denotes the fields to be filled out by the reviewer.

a

— The field can be repeated several times.

b

— The header is the name of the interventions and can be repeated a number of times according to the interventions and subgroups studied.
— The descriptives are represented in the following formats (inside quotation marks only, and [] denotes the fields to be filled by the reviewer):
  (i) Differences: "[mean]±[standard deviation](n=[size])"
  (ii) Associations and regressions: "[number of events]/[total number of events]"

c

— The header is the name of two interventions that represent two-arm comparisons and can be repeated a number of times according to the interventions and subgroups studied.
— The effects sizes are odds ratio for associations and regressions and Hedges' g for differences.
— Bold values for representing statistically significant values (p<.05).
