# Supplementary material for: Ranking sports science and medicine interventions impacting team performance: a protocol for a systematic review and meta-analysis of observational studies in elite football
Source: BMJ Open Sport Exerc Med. 2024 Sep 13;10(3):e002196. doi: 10.1136/bmjsem-2024-002196 (PMC11404162; doi:10.1136/bmjsem-2024-002196)
Supplement: online supplemental file 11 [file bmjsem-10-3-s011.pdf]

```
#
# Supplementary File S11 - Script base to run frequentist and Bayesian meta-regression,
# pairwise, network, and hierarchical meta-analysis.
#
#
#
# INDEX
#
#
# 1. Import data file
# 2. Pairwise
# 3. Meta-regression
# 4. Hierarchical meta-regression
# 5. Network meta-analysis
# 6. Network meta-regression
# 7. Hierarchical network meta-regression
# 8. Bayesian Hierarchical meta-regression
# 9. Bayesian hierarchical network meta-regression
#
#
# Note: The script can be run all at once or one at time.
#
#
#
##################################################
# 1 IMPORT DATA EXCEL FILE
##################################################
#
# if (!require("openxlsx")) install.packages("openxlsx")
# if(!require("Matrix")) install.packages("Matrix")
# if (!require("reshape2")) install.packages("reshape2")
# Function to get the directory of the currently running script
# get_script_dir <- function() {
#   if (rstudioapi::isAvailable()) {
#     return(dirname(rstudioapi::getSourceEditorContext()$path))
#   } else {
#     cmd_args <- commandArgs(trailingOnly = FALSE)
#     file_arg <- "--file="
#     match <- grep(file_arg, cmd_args)
#     if (length(match) > 0) {
#       return(dirname(normalizePath(sub(file_arg, "", cmd_args[match]))))
#     } else {
#       stop("Cannot determine the script's directory.")
#     }
#   }
# }

# Get the script's directory
#script_dir <- get_script_dir()

# Define the path to read the Excel file using the script's location
#file_path <- file.path(script_dir, "data.xlsx")
#cat(file_path)

# Read the data
#meta_data <- read.xlsx(file_path)

###################################################
# 2. PAIRWISE
##################################################
if (!require("meta")) install.packages("meta")
library(meta)

#synthetic data
```

```
set.seed(1)
meta_data <- data.frame(
  study = paste("Study", 1:10),
  outcome = rep("Outcome1", 10),
  treatment1 = rep("Intervention1", 10),
  treatment2 = rep("Intervention2", 10),
  hedgesg = c(5.51, 5.36, 5.77, 5.73, 2.55, 4.46, 4.91, 5.79, 5.28, 3.33),
  var_hedgesg = c(0.35, 0.29, 0.39, 0.45, 0.45, 0.32, 0.14, 0.42, 0.11, 0.42),
  bias = c(1,2,1,2,4,1,2,1,1,5)
)

# meta-analysis function
meta_analysis <- metagen(
  TE = hedgesg,
  seTE = sqrt(var_hedgesg),
  data = meta_data,
  studlab = study,
  comb.fixed = TRUE,
  comb.random = TRUE,
  method.tau = "DL"
)

print(meta_analysis)

# Forest plot
# To save in png
# png(file = "forestplot.png", width = 3800, height = 2400, res = 300)

forest(meta_analysis,
       leftlabs = c("Study", "g", "SE"),
       #layout = "RevMan5"
       )

#dev.off()

# Funnel plot for the meta-analysis
funnel(meta_analysis)

#Subgroup analysis
update(meta_analysis,
       subgroup= bias,
       tau.common = TRUE)

###################################################
# 3. META-REGRESSION
###################################################

if (!require("ggplot2")) install.packages("ggplot2")
if (!require("metafor")) install.packages("metafor")
library(metafor)
library(ggplot2)

# Data
set.seed(1)
meta_reg_data <- data.frame(
  study = paste("Study", 1:10),
  outcome = rep(c("Outcome1", "Outcome2"), each = 5),
  treatment1 = rep("Intervention1", 10),
  treatment2 = rep("Intervention2", 10),
  hedgesg = c(5.51, 5.36, 5.77, 5.73, 2.55, 4.46, 4.91, 5.79, 5.28, 3.33),
  var_hedgesg = c(0.35, 0.29, 0.39, 0.45, 0.45, 0.32, 0.14, 0.42, 0.11, 0.42),
  bias = c(1,2,1,2,4,1,2,1,1,5)
)

meta_reg_data$seTE <- sqrt(meta_reg_data$var_hedgesg)
```

```
meta_reg_data$covariate1 <- c(7, 6, 6, 5, 5, 1, 1, 1, 2, 3)
meta_reg_data$covariate2 <- c(1990, 1998, 1982, 1959, 2003, 2003, 2003, 2004, 2000, 1999)

# Meta-regression function
meta_regression <- rma(yi = hedgesg, sei = seTE, mods = ~ covariate1 + covariate2, data =
meta_reg_data)

#year <-c(1990, 1998, 1982, 1959, 2003, 2003, 2003, 2004, 2000, 1999)
#meta_regression <- metareg(meta_analysis, year)

print(meta_regression)
forest(meta_regression)
funnel(meta_regression)

# Bubble plot only for metareg
#bubble(meta_regression, studlab = TRUE)

# Weights for bubble plot
meta_reg_data$weights <- 1 / sqrt(meta_reg_data$var_hedgesg)

# Bubble plot
ggplot(meta_reg_data, aes(x = covariate2, y = hedgesg, size = weights, label = study)) +
  geom_point(shape = 1, alpha = 0.7) +
  #regression line
  geom_abline(intercept = coef(meta_regression)[1], slope = coef(meta_regression)[2],
linetype = "dashed", color = "blue") +
  theme_bw() +
  theme(legend.position = "none") +
  geom_text(hjust = 1, vjust = 2) +
  labs(title = "Bubble Plot of Meta-Regression Coefficients",
       x = "Covariate 2",
       y = "Effect Size (Hedges' g)") +
  scale_size_continuous(range = c(2, 10))

####################################################
# 4. HIERARCHICAL META-REGRESSION
####################################################

if (!require("ggplot2")) install.packages("ggplot2")
if (!require("metafor")) install.packages("metafor")
library(metafor)
library(ggplot2)

# Data
set.seed(1)
meta_hreg_data <- data.frame(
  study = paste("Study", 1:10),
  outcome = rep(c("Outcome1", "Outcome2"), each = 5),
  treatment1 = rep("Intervention1", 10),
  treatment2 = rep("Intervention2", 10),
  hedgesg = c(5.51, 5.36, 5.77, 5.73, 2.55, 4.46, 4.91, 5.79, 5.28, 3.33),
  var_hedgesg = c(0.35, 0.29, 0.39, 0.45, 0.45, 0.32, 0.14, 0.42, 0.11, 0.42),
  bias = c(1,2,1,2,4,1,2,1,1,5)
)

meta_hreg_data$seTE <- sqrt(meta_hreg_data$var_hedgesg)
meta_hreg_data$covariate1 <- c(7, 6, 6, 5, 5, 1, 1, 1, 2, 3)
meta_hreg_data$covariate2 <- c(1990, 1998, 1982, 1959, 2003, 2003, 2003, 2004, 2000, 1999)

# Hierarchical meta-regression function
meta_hregression <- rma.mv(yi = hedgesg, V = seTE, mods = ~ covariate1 + covariate2,
random = ~ 1 | outcome, data = meta_hreg_data)

summary(meta_hregression)
forest(meta_hregression)
```

```
# Prediction intervals
predict(meta_hregression)

# Effect sizes and variances
yi <- meta_hreg_data$hedgesg
vi <- meta_hreg_data$var_hedgesg

# Weighted mean effect size
wi <- 1 / vi
yi_bar <- sum(wi * yi) / sum(wi)

# Q-statistic
Q <- sum(wi * (yi - yi_bar)^2)
Q

# Residuals
residuals(meta_hregression)

# Weights
meta_hreg_data$weights <- 1 / sqrt(meta_hreg_data$var_hedgesg)

# Extract coefficients for regression line
coef_intercept <- coef(meta_hregression)[1]
coef_slope <- coef(meta_hregression)[2]

# Bubble plot
ggplot(meta_hreg_data, aes(x = covariate1, y = hedgesg, size = weights, label = study)) +
  geom_point(shape = 1, alpha = 0.7) +
  geom_abline(intercept = coef_intercept, slope = coef_slope, linetype = "dashed", color =
"blue") +
  theme_bw() +
  theme(legend.position = "none") +
  geom_text(hjust = 0, vjust = 0) +
  labs(title = "Bubble Plot of Meta-Regression Coefficients",
       x = "Covariate 1",
       y = "Effect Size (Hedges' g)") +
  scale_size_continuous(range = c(2, 10))

####################################################
# 5. NETWORK META-ANALYSIS
####################################################


if (!require("ggplot2")) install.packages("ggplot2")
if (!require("netmeta")) install.packages("netmeta")
if (!require("dplyr")) install.packages("dplyr")
if (!require("metafor")) install.packages("metafor")
library(metafor)
library(dplyr)
library(netmeta)
library(ggplot2)

# data
set.seed(1)
num_studies <- 20
treatments <- c("Intervention1", "Intervention2", "Intervention3", "Intervention4",
"Intervention5")
netmeta_data <- data.frame()
for (i in 1:num_studies) {
  treat_pair <- sample(treatments, 2, replace = FALSE)
  hedgesg <- rnorm(1, 0.5, 0.1)
  var_hedgesg <- runif(1, 0.01, 0.03)
  netmeta_data <- rbind(netmeta_data, data.frame(
    study = paste("Study", i),
```

```
    outcome = "Outcome1",
    treatment1 = treat_pair[1],
    treatment2 = treat_pair[2],
    hedgesg = hedgesg,
    var_hedgesg = var_hedgesg
  ))
}

# Network meta-analysis function
network_meta_analysis <- netmeta(
  TE = hedgesg,
  seTE = sqrt(var_hedgesg),
  treat1 = treatment1,
  treat2 = treatment2,
  studlab = study,
  data = netmeta_data,
  tol.multiarm = 1e-1,
  sm = "SMD",
  details.chkmultiarm = TRUE,
  common = FALSE,
  ref = 'Intervention1'
)

# Graph
netgraph(
  network_meta_analysis,
  plastic = TRUE,
  thickness = "number.of.studies",
  col = "darkblue",                 # Edge color
  lwd = 2,                          # Base edge width
  cex = 1.5,                        # Base node size
  pch.points = 21,                  # Node shape
  col.points = "red",               # Node border color
  bg.points = "red",                # Node fill color
  points = TRUE,                    # Show points
  cex.points = 1 + log(table(netmeta_data$treatment1)),
  lwd.max = 5,                      # Maximum edge width
  lwd.min = 1                       # Minimum edge width
)

# Treatment rankings
treatment_rankings <- netrank(network_meta_analysis)
league_table <- netleague(network_meta_analysis)

plot(
  netrank(network_meta_analysis, small.values = "undesirable"),
  digits = 2
)

print(network_meta_analysis)
forest(network_meta_analysis)
funnel(network_meta_analysis, order = "Intervention1")

# Node splitting
netsplit(network_meta_analysis)
netsplit(network_meta_analysis) %>% forest()

# Heat plot
netheat(network_meta_analysis)
netheat(network_meta_analysis, random = TRUE)

####################################################
# 6. NETWORK META-REGRESSION
####################################################
if (!require("netmeta")) install.packages("netmeta")
```

```
if (!require("metafor")) install.packages("metafor")
library(netmeta)
library(metafor)

#Data
num_studies <- 20
treatments <- c("Intervention1", "Intervention2", "Intervention3", "Intervention4",
"Intervention5")
netmetareg_data <- data.frame()
for (i in 1:num_studies) {
  treat_pair <- sample(treatments, 2, replace = FALSE)
  hedgesg <- rnorm(1, 0.5, 0.1)
  var_hedgesg <- runif(1, 0.01, 0.03)
  netmetareg_data <- rbind(netmetareg_data, data.frame(
    study = paste("Study", i),
    treatment1 = treat_pair[1],
    treatment2 = treat_pair[2],
    hedgesg = hedgesg,
    var_hedgesg = var_hedgesg,
    covariate1 <- sample(1:10, 1),
    covariate2 <- sample(1990:2020, 1)
  ))
}


# Network meta-analysis function
network_meta_analysis <- netmeta(
  TE = hedgesg,
  seTE = sqrt(var_hedgesg),
  treat1 = treatment1,
  treat2 = treatment2,
  studlab = study,
  data = netmetareg_data,
  tol.multiarm = 1e-1,
  sm = "SMD",
  details.chkmultiarm = TRUE,
  common = FALSE,
  ref = 'Intervention1'
)

effect_sizes <- network_meta_analysis$TE
variances <- (network_meta_analysis$seTE)^2

metafor_data <- data.frame(
  TE = effect_sizes,
  varTE = variances,
  covariate1 = rep(netmetareg_data$covariate1, each = 1),
  covariate2 = rep(netmetareg_data$covariate2, each = 1)
)

# Meta-regression
meta_regression <- rma(
  yi = TE,
  vi = varTE,
  mods = ~ covariate1 + covariate2,
  data = metafor_data,
  method = "REML"
)

summary(meta_regression)
forest(meta_regression)
funnel(meta_regression)

# Network graph
netgraph(
```

```
  network_meta_analysis,
  plastic = TRUE,
  thickness = "number.of.studies",
  col = "darkblue",                 # Edge color
  lwd = 2,                          # Base edge width
  cex = 1.5,                        # Base node size
  pch.points = 21,                  # Node shape
  col.points = "red",               # Node border color
  bg.points = "red",                # Node fill color
  points = TRUE,                    # Points nodes
  cex.points = 1 + log(table(netmetareg_data$treatment1)),
  lwd.max = 5,                      # Maximum edge width
  lwd.min = 1                       # Minimum edge width
)

####################################################
#  7. HIERARCHICAL NETWORK META-REGRESSION
####################################################

if (!require("netmeta")) install.packages("netmeta")
if (!require("metafor")) install.packages("metafor")
library(netmeta)
library(metafor)

# Generate synthetic data
set.seed(1)
num_studies <- 20
treatments <- c("Intervention1", "Intervention2", "Intervention3", "Intervention4",
"Intervention5")
hnetmetareg_data <- data.frame()

for (i in 1:num_studies) {
  treat_pair <- sample(treatments, 2, replace = FALSE)
  hedgesg <- rnorm(1, 0.5, 0.1)
  var_hedgesg <- runif(1, 0.01, 0.03)
  hnetmetareg_data <- rbind(hnetmetareg_data, data.frame(
    study = paste("Study", i),
    treatment1 = treat_pair[1],
    treatment2 = treat_pair[2],
    hedgesg = hedgesg,
    var_hedgesg = var_hedgesg,
    covariate1 = sample(1:10, 1),
    covariate2 = sample(1990:2020, 1)
  ))
}

# Define the variance-covariance matrix V as a list of matrices
V_list <- lapply(1:nrow(hnetmetareg_data), function(i)
matrix(hnetmetareg_data$var_hedgesg[i], 1, 1))

# Hierarchical meta-regression model
meta_hnmr <- rma.mv(
  yi = hedgesg,
  V = V_list,
  mods = ~ covariate1 + covariate2,
  random = ~ 1 | study/treatment1/treatment2,
  data = hnetmetareg_data
)

summary(meta_hnmr)

predict(meta_hnmr)

hnetmetareg_data$residuals <- residuals(meta_hnmr)
```

```
# Network meta-analysis with adjustments for residuals from hierarchical model
network_meta_analysis <- netmeta(
  TE = hedgesg - hnetmetareg_data$residuals,
  seTE = sqrt(var_hedgesg),
  treat1 = treatment1,
  treat2 = treatment2,
  studlab = study,
  data = hnetmetareg_data,
  sm = "SMD",
  common = FALSE,
  ref = 'Intervention1'
)

# Plot the network graph
netgraph(
  network_meta_analysis,
  plastic = TRUE,
  thickness = "number.of.studies",
  col = "darkblue",                # Edge color
  lwd = 2,                         # Base edge width
  cex = 1.5,                       # Base node size
  pch.points = 21,                 # Node shape
  col.points = "red",              # Node border color
  bg.points = "red",               # Node fill color
  points = TRUE,                   # Show points
  cex.points = 1 + log(table(hnetmetareg_data$treatment1)),
  lwd.max = 5,                     # Maximum edge width
  lwd.min = 1                      # Minimum edge width
)

# Treatment rankings and league table
treatment_rankings <- netrank(network_meta_analysis)
league_table <- netleague(network_meta_analysis)

# Plot treatment rankings
plot(
  netrank(network_meta_analysis, small.values = "undesirable"),
  digits = 2
)

print(network_meta_analysis)
forest(network_meta_analysis)
funnel(network_meta_analysis, order = "Intervention1")

print(summary(meta_hnmr))

####################################################
# 8. BAYESIAN HIERARCHICAL META-REGRESSION
####################################################

# #download bugs
# #https://drive.google.com/drive/folders/1XzG681AaZpRtPtynkuGUt2BspMTvhYoP
# #Linux
# #cd openbugs
# #unzip OpenBUGS323.zip
# #sudo mv OpenBUGS323 /usr/local/OpenBUGS
# #If linux install wine
# #sudo apt-get update
# #sudo apt-get install wine
# # echo 'export PATH=$PATH:/usr/local/OpenBUGS/bin' >> ~/.bashrc
# # source ~/.bashrc
# # sudo chmod +x /usr/local/OpenBUGS/OpenBUGS.exe
# #echo 'export PATH=$PATH:/usr/bin/wine' >> ~/.bashrc
# #source ~/.bashrc
# # Set environment variables for OpenBUGS and Wine
```

```
# Sys.setenv(WINE = "/usr/bin/wine")
# Sys.setenv(WINEPATH = "/usr/bin/winepath")
# Sys.setenv(OpenBUGS_PGM = "/usr/local/OpenBUGS/OpenBUGS.exe")
# #Windows
# #download and install OpenBugs
https://openbugs.software.informer.com/download/#downloading
Sys.setenv(OpenBUGS_PGM = "C:/Program Files (x86)/OpenBUGS/OpenBUGS323/OpenBUGS.exe")
#Sys.setenv(WINBUGS_PGM = "C:/Program Files (x86)/WinBUGS14/WinBUGS14.exe")


if (!require("R2OpenBUGS")) install.packages("R2OpenBUGS")
if (!require("metafor")) install.packages("metafor")
library(metafor)
library(R2OpenBUGS)


# Define the model as a string
model_string <- "
model {
  for (i in 1:N) {
    y[i] ~ dnorm(mu[i], tau[i])
    mu[i] <- b0 + b1 * covariate1[i] + b2 * covariate2[i] + u[outcome[i]]
    tau[i] <- 1 / (se[i] * se[i])
    y_rep[i] ~ dnorm(mu[i], tau[i])  # Posterior predictive replication
  }

  # Priors
  b0 ~ dnorm(0, 0.001)
  b1 ~ dnorm(0, 0.001)
  b2 ~ dnorm(0, 0.001)

  for (j in 1:K) {
    u[j] ~ dnorm(0, tau_u)
  }
  tau_u <- 1 / (sigma_u * sigma_u)
  sigma_u ~ dunif(0, 10)
}
"
writeLines(model_string, con = "model.txt")

#Data
set.seed(123)
meta_reg_data <- data.frame(
  hedgesg = rnorm(10),
  seTE = runif(10, 0.1, 0.3),
  covariate1 = rnorm(10),
  covariate2 = rnorm(10),
  outcome = factor(rep(1:5, each = 2))
)

meta_regression <- rma.mv(yi = hedgesg, V = seTE^2, mods = ~ covariate1 + covariate2,
random = ~ 1 | outcome, data = meta_reg_data)

# Residuals to the data
meta_reg_data$residuals <- residuals(meta_regression)

# Prepare data for BUGS
data_list <- list(
  y = meta_reg_data$hedgesg,
  se = meta_reg_data$seTE,
  covariate1 = meta_reg_data$covariate1,
  covariate2 = meta_reg_data$covariate2,
  outcome = as.numeric(meta_reg_data$outcome),
  N = nrow(meta_reg_data),
  K = length(unique(meta_reg_data$outcome))
```

```
)

# Initial values
inits <- function() {
  list(b0 = 0, b1 = 0, b2 = 0, sigma_u = 1)
}

# Parameters
params <- c("b0", "b1", "b2", "sigma_u", "y_rep")

# Number of chains, iterations, burn-in period, and thinning
n.chains <- 4
n.iter <- 10000
n.burnin <- 5000
n.thin <- 10

# BUGS model to run in Linux and Windows
bugs_model <- bugs(
  data = data_list,
  inits = inits,
  parameters.to.save = params,
  model.file = "model.txt",
  n.chains = n.chains,
  n.iter = n.iter,
  n.burnin = n.burnin,
  n.thin = n.thin,
  DIC = TRUE,
  OpenBUGS.pgm = Sys.getenv("OpenBUGS_PGM"),
  useWINE = FALSE, #For Linux TRUE
  #WINE = Sys.getenv("WINE"), #Linux
  newWINE = TRUE,
  #bugs.directory = "C:/Program Files (x86)/WinBUGS14", #windows winbugs
  #WINEPATH = Sys.getenv("WINEPATH"), #Linux
  debug = TRUE
)

print(bugs_model)
plot(bugs_model)

# Extract replicated data for posterior predictive checks
if ("y_rep" %in% names(bugs_model$sims.list)) {
  y_rep_samples <- bugs_model$sims.list$y_rep
  y_rep_mean <- apply(y_rep_samples, 2, mean, na.rm = TRUE)
  y_rep_sd <- apply(y_rep_samples, 2, sd, na.rm = TRUE)
  observed_length <- length(meta_reg_data$hedgesg)
  predicted_length <- length(y_rep_mean)
  if (observed_length == predicted_length) {
    plot(meta_reg_data$hedgesg, y_rep_mean, xlab = "Observed", ylab = "Predicted", main =
"Posterior Predictive Check")
    abline(0, 1, col = "red")
  } else {
    warning("Lengths of observed and predicted data do not match.")
  }
} else {
  warning("'y_rep' not found in bugs_model$sims.list. Check saved parameters.")
}

####################################################
#  9. BAYESIAN HIERARHICAL NETWORK META-REGRESSION
####################################################

if (!require("R2OpenBUGS")) install.packages("R2OpenBUGS")
if (!require("metafor")) install.packages("metafor")
if (!require("netmeta")) install.packages("netmeta")
if (!require("coda")) install.packages("coda")
```

```
library(metafor)
library(R2OpenBUGS)
library(netmeta)
library(coda)

# Set OpenBUGS
# Sys.setenv(WINE = "/usr/bin/wine")
# Sys.setenv(WINEPATH = "/usr/bin/winepath")
# Sys.setenv(OpenBUGS_PGM = "/usr/local/OpenBUGS/OpenBUGS.exe")
# #Windows
# #download and install OpenBugs
https://openbugs.software.informer.com/download/#downloading
Sys.setenv(OpenBUGS_PGM = "C:/Program Files (x86)/OpenBUGS/OpenBUGS323/OpenBUGS.exe")
#Sys.setenv(WINBUGS_PGM = "C:/Program Files (x86)/WinBUGS14/WinBUGS14.exe")

# Data
set.seed(123)
bayesnetreg_data <- data.frame(
  hedgesg = c(0.2, -0.1, 0.3, -0.2, 0.4, -0.3, 0.5, -0.4, 0.6, -0.5),
  var_hedgesg = rep(0.05, 10),
  covariate1 = rnorm(10),
  covariate2 = rnorm(10),
  treatment1 = factor(rep(1:5, each = 2)),
  treatment2 = factor(rep(2:6, each = 2)),
  study = factor(rep(1:5, each = 2))
)

hierarchical_meta <- rma.uni(yi = hedgesg, sei = sqrt(var_hedgesg), data =
bayesnetreg_data)
# Residuals from the hierarchical meta-analysis
bayesnetreg_data$residuals <- residuals(hierarchical_meta)

# Bayesian model file
model_file <- "hierar_network_meta_model.txt"
writeLines(con = model_file, text =
             "model {
  # Level 1: Within-study model
  for (i in 1:N) {
    hedgesg[i] ~ dnorm(mu[i], tau)
    mu[i] <- study_effect[study[i]] + beta[1] * covariate1[i] + beta[2] * covariate2[i] +
delta[treatment1[i], treatment2[i]]
    # Replicated data
    y_rep[i] ~ dnorm(mu[i], tau)
  }

  # Level 2: Between-study model
  for (s in 1:S) {
    study_effect[s] ~ dnorm(0, tau_study)
  }

  # Initialize the delta matrix
  for (j in 1:(n_treatments-1)) {
    for (k in (j+1):n_treatments) {
      delta[j, k] ~ dnorm(0, tau_delta)
      delta[k, j] <- -delta[j, k]
    }
  }
  for (j in 1:n_treatments) {
    delta[j, j] <- 0
  }

  # Priors for regression coefficients
  beta[1] ~ dnorm(0, 0.001)
  beta[2] ~ dnorm(0, 0.001)
```

```
  # Priors for precisions (inverse variances)
  tau ~ dgamma(0.5, 0.1)
  tau_delta ~ dgamma(0.5, 0.1)
  tau_study ~ dgamma(0.5, 0.1)

  # Initial values for precision parameters
  #tau_study <- 1
  #tau_delta <- 1
  #tau <- 1
}")



# Data for OpenBUGS
data_list <- list(
  hedgesg = bayesnetreg_data$hedgesg,
  covariate1 = bayesnetreg_data$covariate1,
  covariate2 = bayesnetreg_data$covariate2,
  treatment1 = as.numeric(as.character(bayesnetreg_data$treatment1)),
  treatment2 = as.numeric(as.character(bayesnetreg_data$treatment2)),
  residuals = bayesnetreg_data$residuals,
  study = as.numeric(as.character(bayesnetreg_data$study)),
  N = nrow(bayesnetreg_data),
  S = length(unique(bayesnetreg_data$study)),
  n_treatments = length(unique(c(bayesnetreg_data$treatment1,
bayesnetreg_data$treatment2)))
)


# Initial values
inits <- function() {
  delta_matrix <- matrix(rnorm(data_list$n_treatments, 0, 0.1),
                         nrow = data_list$n_treatments,
                         ncol = data_list$n_treatments)

  # Set the diagonal elements to zero
  diag(delta_matrix) <- 0

  # Return the list of initial values
  list(
    beta = rnorm(2, 0, 0.1),  # Smaller standard deviation for better convergence
    tau = rgamma(1, 0.5, 0.1),  # Ensure a positive initial value
    tau_delta = rgamma(1, 0.5, 0.1),  # Ensure a positive initial value
    tau_study = rgamma(1, 0.5, 0.1),  # Ensure a positive initial value
    delta = delta_matrix,
    study_effect = rnorm(data_list$S, 0, 0.1)  # Initial values for study effects
  )
}

# Run the OpenBUGS model
bugs_model <- bugs(
  data = data_list,
  inits = NULL,
  parameters.to.save = c("beta", "tau", "tau_delta", "tau_study", "delta", "study_effect",
"y_rep"),
  model.file = model_file,
  n.chains = 3,
  n.iter = 10000,
  n.burnin = 5000,
  n.thin = 10,
  DIC = TRUE,
  OpenBUGS.pgm = Sys.getenv("OpenBUGS_PGM"),
  useWINE = FALSE, # For Linux TRUE
  #WINE = "/usr/bin/wine",
  #newWINE = TRUE,
```

```
  #WINEPATH = "/usr/bin/winepath",
  debug=TRUE,
  codaPkg =TRUE
)


print(bugs_model)
coda_files <- bugs_model  # Assumes that bugs_model contains the paths to CODA files
file1_lines <- readLines(coda_files[1], n = 10)
file2_lines <- readLines(coda_files[2], n = 10)
index_file <- sub("chain\\d+", "index", coda_files[1])  # Construct index file path
chain1 <- read.coda(coda_files[1], index_file)
chain2 <- read.coda(coda_files[2], index_file)
chain3 <- read.coda(coda_files[3], index_file)

mcmc_list <- mcmc.list(chain1, chain2, chain3)
str(mcmc_list)
plot(mcmc_list)

beta_samples <- as.matrix(mcmc_list[, grep("^beta", varnames(mcmc_list))])
beta_mean <- apply(beta_samples, 2, mean)
beta_sd <- apply(beta_samples, 2, sd)

cat("Beta Mean:\n", beta_mean, "\n")
cat("Beta SD:\n", beta_sd, "\n")

delta_samples <- as.matrix(mcmc_list[, grep("^delta", varnames(mcmc_list))])
delta_mean <- apply(delta_samples, 2, mean)
delta_sd <- apply(delta_samples, 2, sd)


cat("Delta Mean:\n", delta_mean, "\n")
cat("Delta SD:\n", delta_sd, "\n")

# Plot for beta parameters
plot(beta_mean, ylab="Mean", xlab="Beta Parameters", main="Beta Parameter Means")
abline(h=0, col="red")

# Plot for delta parameters
plot(delta_mean, ylab="Mean", xlab="Delta Parameters", main="Delta Parameter Means")
abline(h=0, col="red")

# Treatments
mcmc_list <- mcmc.list(mcmc_list[[1]], mcmc_list[[2]], mcmc_list[[3]])
delta_samples <- as.matrix(mcmc_list[, grep("^delta", varnames(mcmc_list))])

# Dimensions of delta_samples
n_samples <- nrow(delta_samples)
n_deltas <- ncol(delta_samples)

n_treatments <- length(unique(c(bayesnetreg_data$treatment1,
bayesnetreg_data$treatment2)))
delta_array <- array(NA, dim = c(n_samples, n_treatments, n_treatments))
delta_names <- varnames(mcmc_list)[grep("^delta", varnames(mcmc_list))]
for (k in 1:n_deltas) {
  indices <- as.numeric(unlist(strsplit(gsub("delta\\[|\\]", "", delta_names[k]), ",")))
  delta_array[, indices[1], indices[2]] <- delta_samples[, k]
  delta_array[, indices[2], indices[1]] <- -delta_samples[, k]  # Ensure symmetry
}

delta_mean <- apply(delta_array, c(2, 3), mean, na.rm = TRUE)

treatment_rankings <- rank(rowMeans(delta_mean, na.rm = TRUE), ties.method = "average")

league_table <- matrix(NA, nrow = n_treatments, ncol = n_treatments)
```

```
for (i in 1:n_treatments) {
  for (j in 1:n_treatments) {
    if (i != j) {
      league_table[i, j] <- delta_mean[i, j]
    } else {
      league_table[i, j] <- NA
    }
  }
}

print(treatment_rankings)

print(league_table)

plot(treatment_rankings, type = "b", main = "Treatment Rankings", xlab = "Treatment", ylab
= "Rank", pch = 19)
```
